# Author Correction: Tanc2-mediated mTOR inhibition balances mTORC1/2 signaling in the developing mouse brain and human neurons

**DOI:** 10.1038/s41467-022-33972-9

**Published:** 2022-11-01

**Authors:** Sun-Gyun Kim, Suho Lee, Yangsik Kim, Jieun Park, Doyeon Woo, Dayeon Kim, Yan Li, Wangyong Shin, Hyunjeong Kang, Chaehyun Yook, Minji Lee, Kyungdeok Kim, Junyeop Daniel Roh, Jeseung Ryu, Hwajin Jung, Seung Min Um, Esther Yang, Hyun Kim, Jinju Han, Won Do Heo, Eunjoon Kim

**Affiliations:** 1 Center for Synaptic Brain Dysfunctions, Institute for Basic Science (IBS), Daejeon, Korea; 2 Graduate School of Medical Science and Engineering, Korea Advanced Institute for Science and Technology (KAIST), Daejeon, Korea; 3 Department of Biological Sciences, KAIST, Daejeon, Korea; 4 Center for Cognition and Sociality, Institute for Basic Science (IBS), Daejeon, Korea; 5 Department of Anatomy and Division of Brain Korea 21, Biomedical Science, College of Medicine, Korea University, Seoul, Korea

**Keywords:** Cell signalling, Cellular neuroscience, Neuronal development, Molecular neuroscience

Correction to: *Nature Communications* 10.1038/s41467-021-22908-4, published online 11 May 2021

The original version of this Article contained an error in a sentence in the Methods section, under the subheading ‘Human neuron culture and lentivirus infection’, in which an incorrect IRB number was used. The sentence incorrectly read ‘Protocols describing the use of human ESCs were approved in accordance with the ethical requirements and regulations of the Institutional Review Board of KAIST (IRB #KA2018-61).’ The correct version states ‘KH2017-109’ in place of ‘KA2018-61’. This has been corrected in both the PDF and HTML versions of the Article.

