# Supplementary material for: Tanc2-mediated mTOR inhibition balances mTORC1/2 signaling in the developing mouse brain and human neurons
Source: Nat Commun. 2021 May 11;12:2695. doi: 10.1038/s41467-021-22908-4 (PMC8113471; doi:10.1038/s41467-021-22908-4)
Supplement: Supplementary file 1 — Supplementary Information [file 41467_2021_22908_MOESM1_ESM.pdf]

Supplementary Information

Tanc2-mediated mTOR inhibition balances mTORC1/2 signaling in the developing mouse brain and human neurons

Sun-Gyun Kim,[1,*] Suho Lee,[1,*] Yangsik Kim,[2] Jieun Park,[3] Doyeon Woo,[4] Dayeon Kim,[5] Yan Li,[1] Wangyong Shin,[1] Hyunjeong Kang,[3] Chaehyun Yook,[3] Minji Lee,[4] Kyungdeok Kim,[1] Junyeop Daniel Roh,[3] Jeseung Ryu,[3] Hwajin Jung,[1] Seung Min Um,[3] Esther Yang,[6] Hyun Kim,[6] Jinju Han,[5] Won Do Heo,[3,4] and Eunjoon Kim[1,3,#]

[1]Center for Synaptic Brain Dysfunctions, Institute for Basic Science (IBS), Daejeon 34141, Korea; [2]Graduate School of Medical Science and Engineering, Korea Advanced Institute for Science and Technology (KAIST); [3]Department of Biological Sciences, KAIST, Daejeon 34141, Korea; [4]Center for Cognition and Sociality, Institute for Basic Science (IBS), Daejeon 34141, Korea; [5]Graduate School of Medical Science and Engineering, KAIST, Daejeon 34141, Korea; [6]Department of Anatomy and Division of Brain Korea 21, Biomedical Science, College of Medicine, Korea University, Seoul, Korea; *These authors contributed equally to the work; [#]Correspondence: kime@kaist.ac.kr.

Supplementary figures and legends

Supplementary Fig. 1

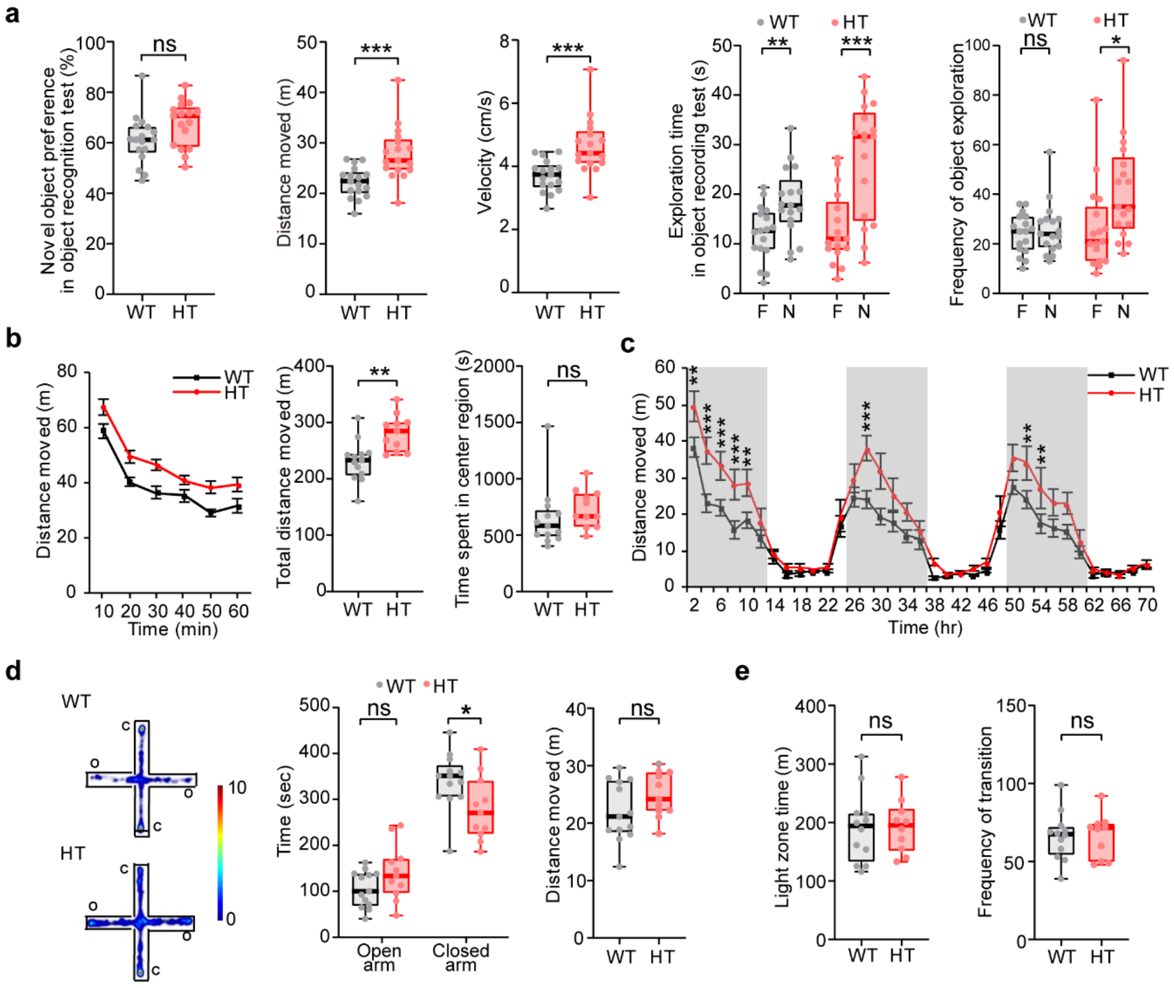

Supplementary Fig. 1. Hyperactivity and moderate anxiolytic-like behavior in male *Tanc2*$^{+/-}$ mice.

(a) Normal novel object-recognition memory in *Tanc2*$^{+/-}$ mice (2–5 months; male) in the novel object-recognition test, as shown by novel object preference (% time spent in exploring a novel object relative to the total time spent exploring both familiar and novel objects). F, familiar object; N, new object. Note that these mice also showed hyperactivity in the novel object arena, as supported by the distance moved and movement velocity. The increased frequency of novel object exploration in the mutant mice might be attributable to the increased hyperactivity. Data: minimal, maximal, median, 25%, and 75% values. (n = 17 [WT, HT], *P < 0.05, **P < 0.01, ***P < 0.001, ns, not significant, Student's t-test).

(b) Hyperactivity of *Tanc2*$^{+/-}$ mice (2–5 months; male) in the open-field test, as shown by distance moved over 60 minutes and total distance moved. Note also that the

time spent in the center region of the open field arena was normal in *Tanc2*$^{+/–}$ mice, indicative of normal anxiety-like behavior. Data: mean ± SEM (line graphs), minimal, maximal, median, 25%, and 75% values. (n = 13 [WT], 11 [HT], **P < 0.01, ns, not significant, two-way RM ANOVA and Student's t-test).

(c) Hyperactivity of *Tanc2*$^{+/–}$ mice (2–5 months; male) in the Laboras test, as shown by distance moved over 72 hours. The shaded areas represent light-off periods. Data: mean ± SEM. (n = 16 [WT, HT], **P < 0.01, ***P < 0.001, two-way RM ANOVA [genotype effect P < 0.05]).

(d) Moderate anxiolytic-like behavior of *Tanc2*$^{+/–}$ mice (2–5 months; male) in the elevated plus-maze test, as shown by time spent in open and closed arms. There was no genotype difference in the total distance moved. Data: minimal, maximal, median, 25%, and 75% values. (n = 13 [WT], 11 [HT], *P < 0.05, ns, not significant, two-way RM ANOVA with Bonferroni test and Student's t-test).

(e) Normal anxiety-like behavior of *Tanc2*$^{+/–}$ mice (2–5 months; male) in the light-dark test, as shown by time spent in the light chamber/zone and frequency of transitions between light and dark chambers. Data: minimal, maximal, median, 25%, and 75% values. (n = 13 [WT], 10 [HT], ns, not significant, Student's t-test).

See Source Data 1 for raw data values and Supplementary Table 1 for statistical details.

Supplementary Fig. 2

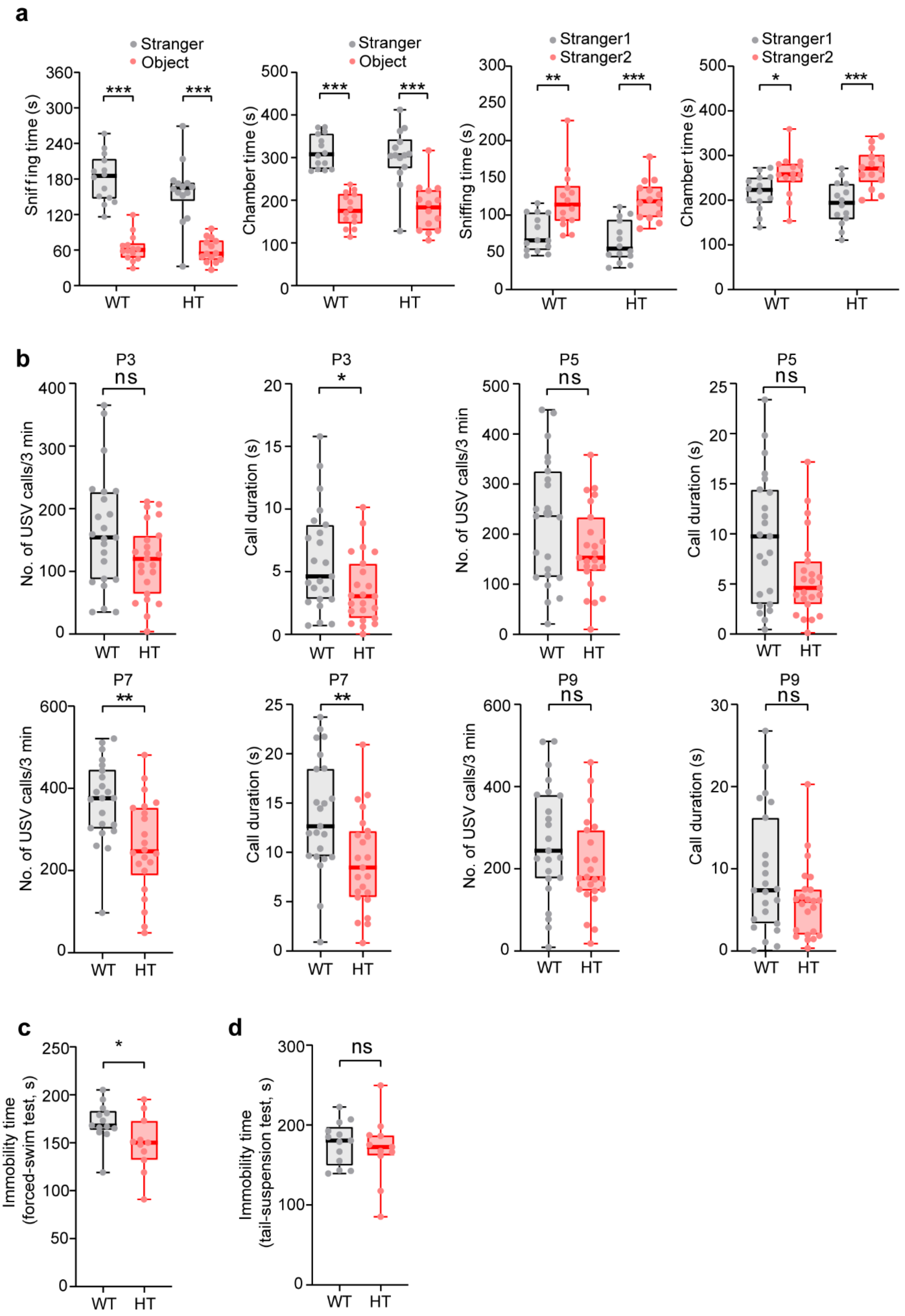

Supplementary Fig. 2. Suppressed pup ultrasonic vocalization but normal social interaction, social novelty recognition, and depression-like behavior in male *Tanc2*$^{+/-}$ mice.

(a) Normal social interaction and social-novelty recognition in *Tanc2*$^{+/-}$ mice (2–5 months; male) in the three-chamber social-interaction test, as shown by time spent in the chamber and in sniffing the target (stranger/object). Stranger 1, social stranger mouse; Object, inanimate object; Stranger 2, new social stranger mouse. Data: minimal, maximal, median, 25%, and 75% values. (n = 14 mice [WT, HT], ***P < 0.001, Student's t-test).

(b) Modestly suppressed ultrasonic vocalizations (USVs) in *Tanc2*$^{+/-}$ pups (P3–9; male) separated from their mothers, as shown by the total number of USV calls and the duration of each call. Data: minimal, maximal, median, 25%, and 75% values. n = 23 pups [WT, HT], *P < 0.05, **P < 0.01, ns, not significant, Student's t-test).

(c) Anti-depression-like behavior of *Tanc2*$^{+/-}$ mice (2–5 months; male) in the forced-swim test, as shown by immobility time. Data: minimal, maximal, median, 25%, and 75% values. (n = 13 [WT], 11 [HT], *P < 0.05, Student's t-test).

(d) Normal depression-like behavior of *Tanc2*$^{+/-}$ mice (2–5 months; male) in the tail-suspension test, as shown by immobility time. Data: minimal, maximal, median, 25%, and 75% values. (n = 13 [WT], 11 [HT], ns, not significant, Student's t-test).

See Source Data 1 for raw data values and Supplementary Table 1 for statistical details.

Supplementary Fig. 3

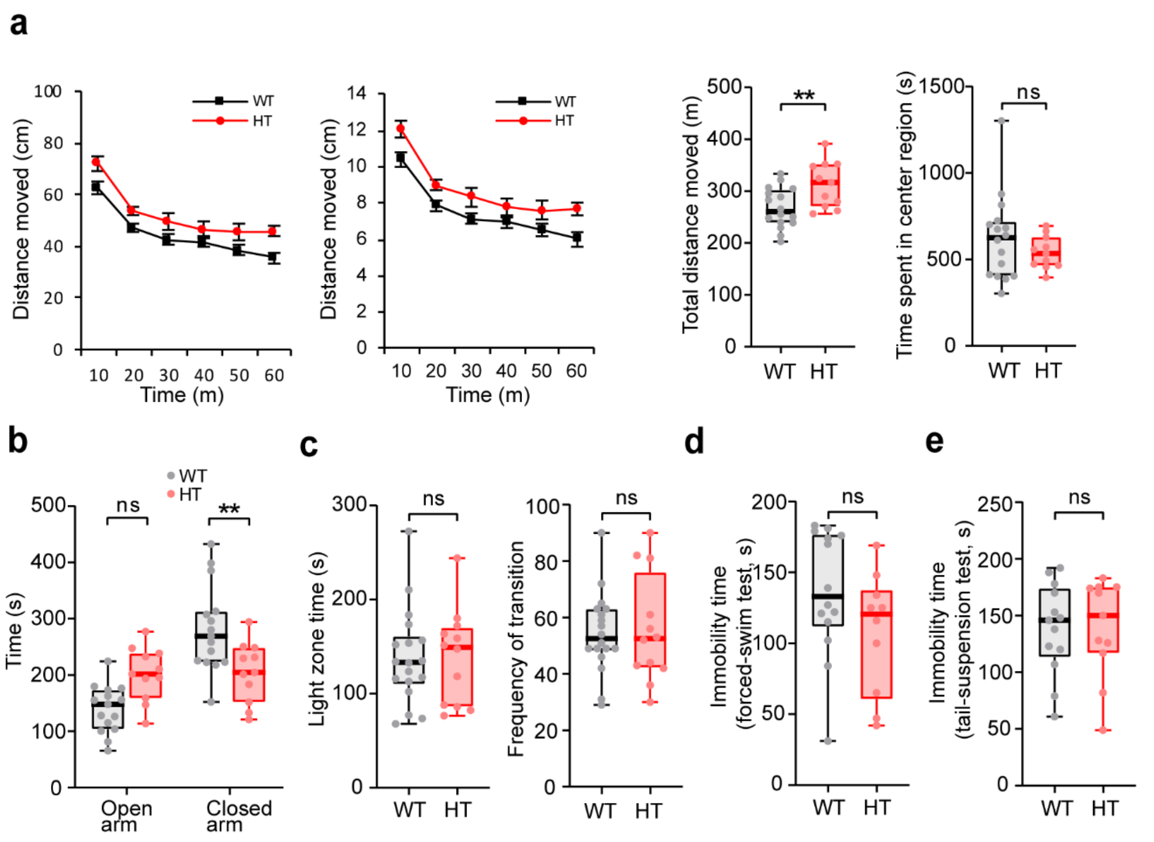

Supplementary Fig. 3. Female *Tanc2*$^{+/-}$ mice display hyperactivity and anxiolytic-like behavior, but normal depression-like behavior.

(a) Hyperactivity of female *Tanc2*$^{+/-}$ mice (2–5 months) in the open-field test, as shown by distance moved over 60 minutes and total distance moved. Note also that the time spent in the center region of the open field arena was normal in female *Tanc2*$^{+/-}$ mice, indicative of normal anxiety-like behavior. Data: mean ± SEM (line gr aphs), minimal, maximal, median, 25%, and 75% values. (n = 16 mice [WT], and 11 mice [HT], **P < 0.01, ns, not significant, two-way RM ANOVA and Student's t-test).

(b) Moderate anxiolytic-like behavior of female *Tanc2*$^{+/-}$ mice (2–5 months) in the elevated plus-maze test, as shown by time spent in open and closed arms. Data: minimal, maximal, median, 25%, and 75% values. (n = 15 mice [WT], and 11 mice [HT], **P < 0.01, ns, not significant, two-way RM ANOVA with Bonferroni test).

(c) Normal anxiety-like behavior of female *Tanc2*$^{+/-}$ mice (2–5 months) in the light-dark test, as shown by time spent in the light chamber and frequency of transitions between light and dark chambers. Data: minimal, maximal, median, 25%, and 75% values. (n = 18 mice [WT], and 12 mice [HT], ns, not significant, Student's t-test).

(d) Normal depression-like behavior of female *Tanc2*$^{+/-}$ mice (2–5 months) in the

forced-swim test, as shown by immobility time. Data: minimal, maximal, median, 25%, and 75% values. (n = 14 mice [WT], and 10 mice [HT], ns, not significant, Student's t-test).

(e) Normal depression-like behavior of *Tanc2*$^{+/-}$ mice (2–5 months; female) in the tail-suspension test, as shown by immobility time. Data: minimal, maximal, median, 25%, and 75% values. (n = 13 mice [WT], and 10 mice [HT], ns, not significant, Student's t-test).

See Source Data 1 for raw data values and Supplementary Table 1 for statistical details.

Supplementary Fig. 4

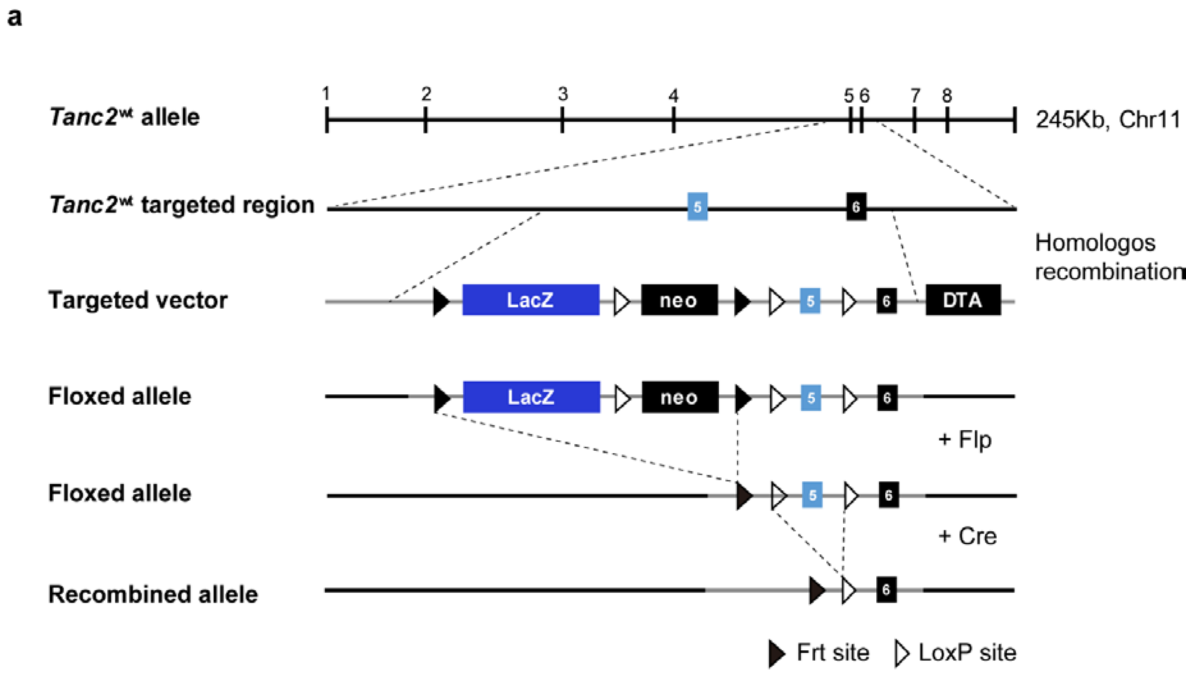

Supplementary Fig. 4. Conditional *Tanc2* KO strategy.

(a) Scheme for the generation of *Tanc2*$^{fl/fl}$ mice carrying floxed exon 5 of the *Tanc2* gene. LacZ, β-galactosidase gene; neo, neomycin resistance cassette; DTA, diphtheria toxin gene; Flp, flippase; Cre, Cre recombinase; Frt site, site for flippase; LoxP site, cleavage site for cre recombinase action.

Supplementary Fig. 5

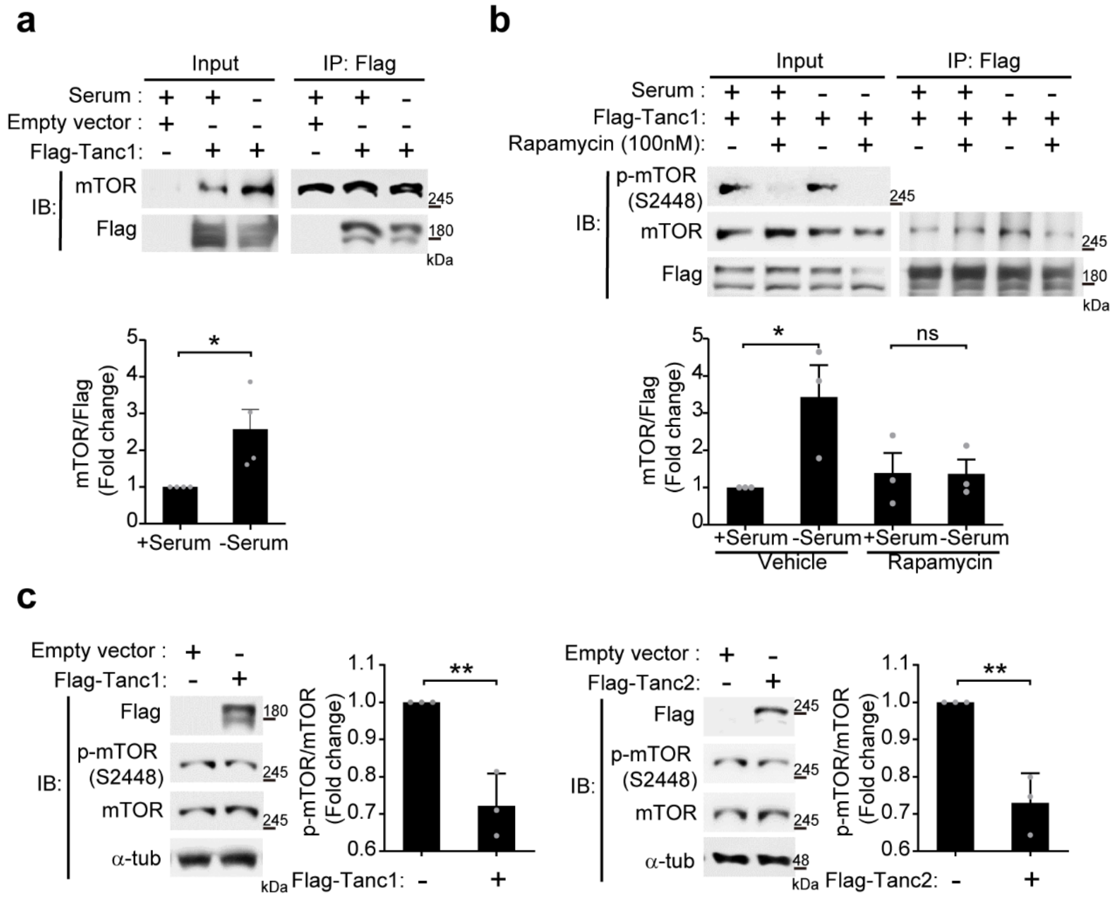

Supplementary Fig. 5. Tanc1 interacts with mTOR in a serum- and rapamycin-dependent manner and inhibits mTOR activity in heterologous Cells.

(a) Serum starvation (4 hours) promotes the interaction between Tanc1 and mTOR in heterologous cells, as demonstrated by coimmunoprecipitation. HEK293T cells expressing Flag-Tanc1 (human) were incubated with serum (or no serum; control) for 4 hours prior to coimmunoprecipitation and immunoblot experiments. mTOR signals were normalized to Tanc1 signals for quantification. Data: mean ± SD. (n = 4 independent experiments, *P < 0.05, Student's t-test).

(b) Rapamycin inhibits serum starvation-induced increases in the interaction between Tanc1 and mTOR in heterologous cells. Flag-Tanc1 was expressed in HEK293T cells in the presence of serum. Pretreatment of cells with rapamycin for 2 hours was followed by serum starvation for 4 hours; cell lysates were subjected to coIP and immunoblot experiments. mTOR signals were normalized to Tanc1 signals for quantification. Data: mean ± SD. (n = 3 independent experiments, ***P < 0.05, ns, not significant, Student's t-test).

(c) Overexpression of Tanc1 in HEK293T cells suppresses mTOR activity, as shown

by the ratio of total to phosphorylated (active) mTOR. Tanc2 (human) was also used as a control. Flag-tagged Tanc1/Tanc2 were overexpressed in HEK293T cells, followed by immunoblotting for total and phosphorylated (Ser-2448) mTOR. mTOR signals were normalized to α-tubulin signals. Data: mean $\pm$ SD. (n = 3 independent experiments, $^{**}P < 0.01$, Student's t-test).

See Source Data 1 for raw data values and Supplementary Table 1 for statistical details.

Supplementary Fig. 6

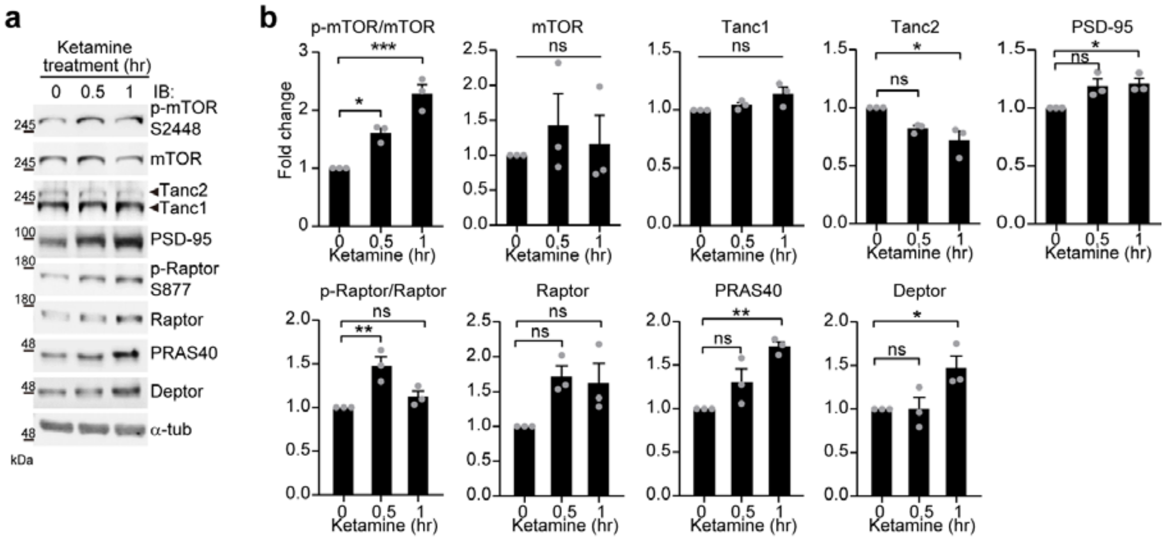

Supplementary Fig. 6. Ketamine induces changes in mTOR activity and synaptic levels of Tanc2-associated and mTORC proteins in the mouse brain.

(a and b) Ketamine induces mTOR activation in the mouse brain (P13–14; 10 mg/kg; i.p.), as shown by the time-dependent increases in the phosphorylation of mTOR and the mTORC1 protein Raptor in crude synaptosomes. Note that synaptic levels of other mTORC1/2 proteins (PRAS40 and Deptor) and PSD-95 also increased, whereas those of Tanc2 (but not Tanc1) were modestly decreased. Data: mean ± S D. (n = 3 independent experiments, *P < 0.05, **P < 0.01, ***P < 0.001, ns, not significant, one-way ANOVA with Bonferroni test).

See Source Data 1 for raw data values and Supplementary Table 1 for statistical details.

Supplementary Fig. 7

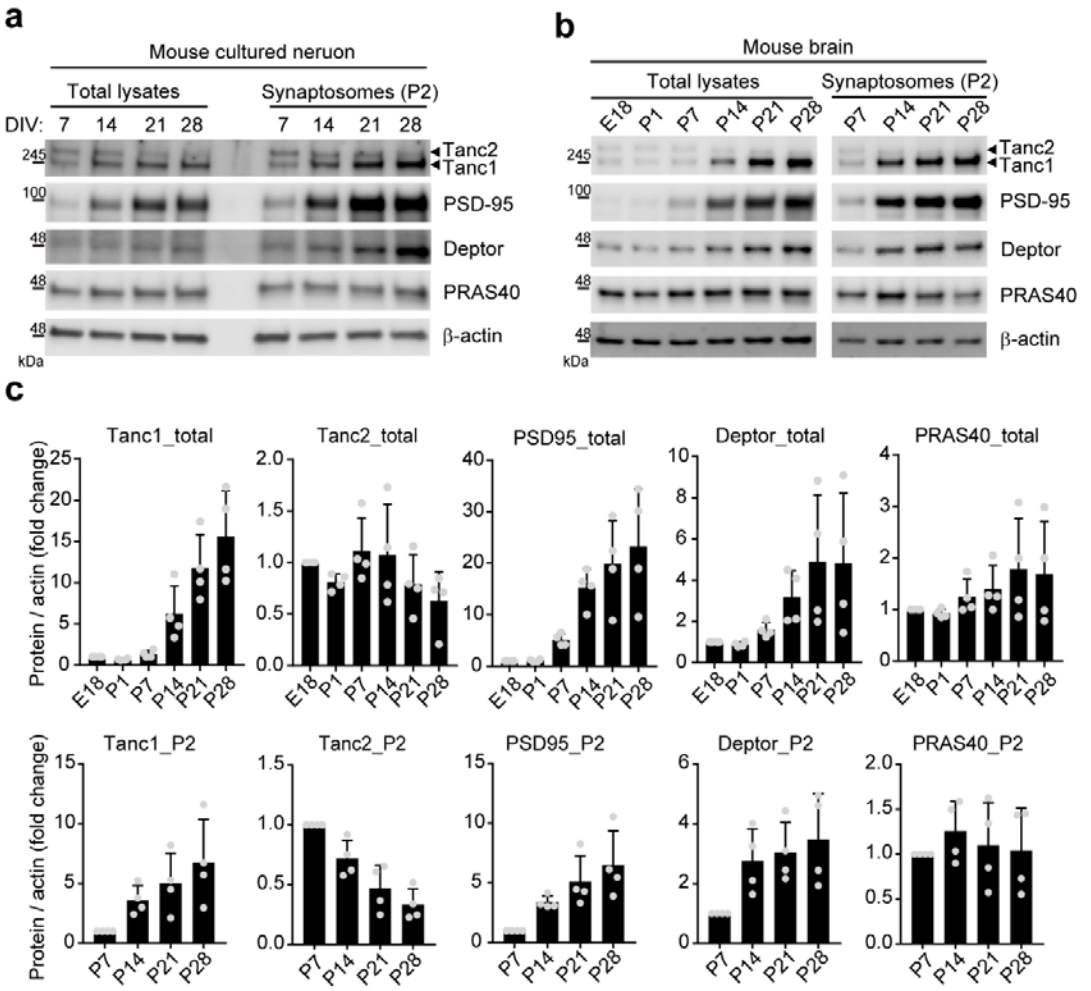

Supplementary Fig. 7. Temporal changes in total levels and synaptic enrichment of Tanc2, Deptor, and Tanc1 in cultured mouse hippocampal neurons and the mouse brain.

(a) Western blot analyses of whole lysates from cultured mouse hippocampal neurons show Tanc2 protein levels that are largely stable across developmental stages (DIV/days in vitro 7–28) and stronger relative to Tanc1 at early stages, while Deptor and Tanc1 protein levels gradually increase. Western blot analyses of crude synaptosomes from cultured mouse hippocampal neurons show largely unchanged synaptic levels of Tanc2 proteins across the developmental stages, which contrasts with the increasing synaptic levels of Deptor and Tanc1 proteins. Total and synaptic levels of PRAS40 (a component of mTORC1) were largely unchanged across developmental stages. PSD-95 was used as a positive control for stage-dependent increases in total expression and synaptic enrichment, and β-actin was used as a

loading control.

(b and c) Western blot analyses of whole lysates from the mouse brain (in vivo results) show Tanc2 protein levels that are largely stable across developmental stages (E/embryonic day 18–P/postnatal day 28) and higher relative to Tanc1 at early stages, while Deptor and Tanc1 protein levels gradually increase. Western blot analyses of crude synaptosomes from the mouse brain show decreasing synaptic levels of Tanc2 proteins across the developmental stages, which contrasts with the increasing synaptic levels of Deptor and Tanc1 proteins. Total and synaptic levels of PRAS40 were largely unchanged across developmental stages. PSD-95 was used as a positive control for stage-dependent increases in total expression and synaptic enrichment, and β-actin was used as a loading control. Data: mean ± SD. (n = 4 independent experiments).

See Source Data 1 for raw data values and Supplementary Table 1 for statistical details.

Supplementary Fig. 8

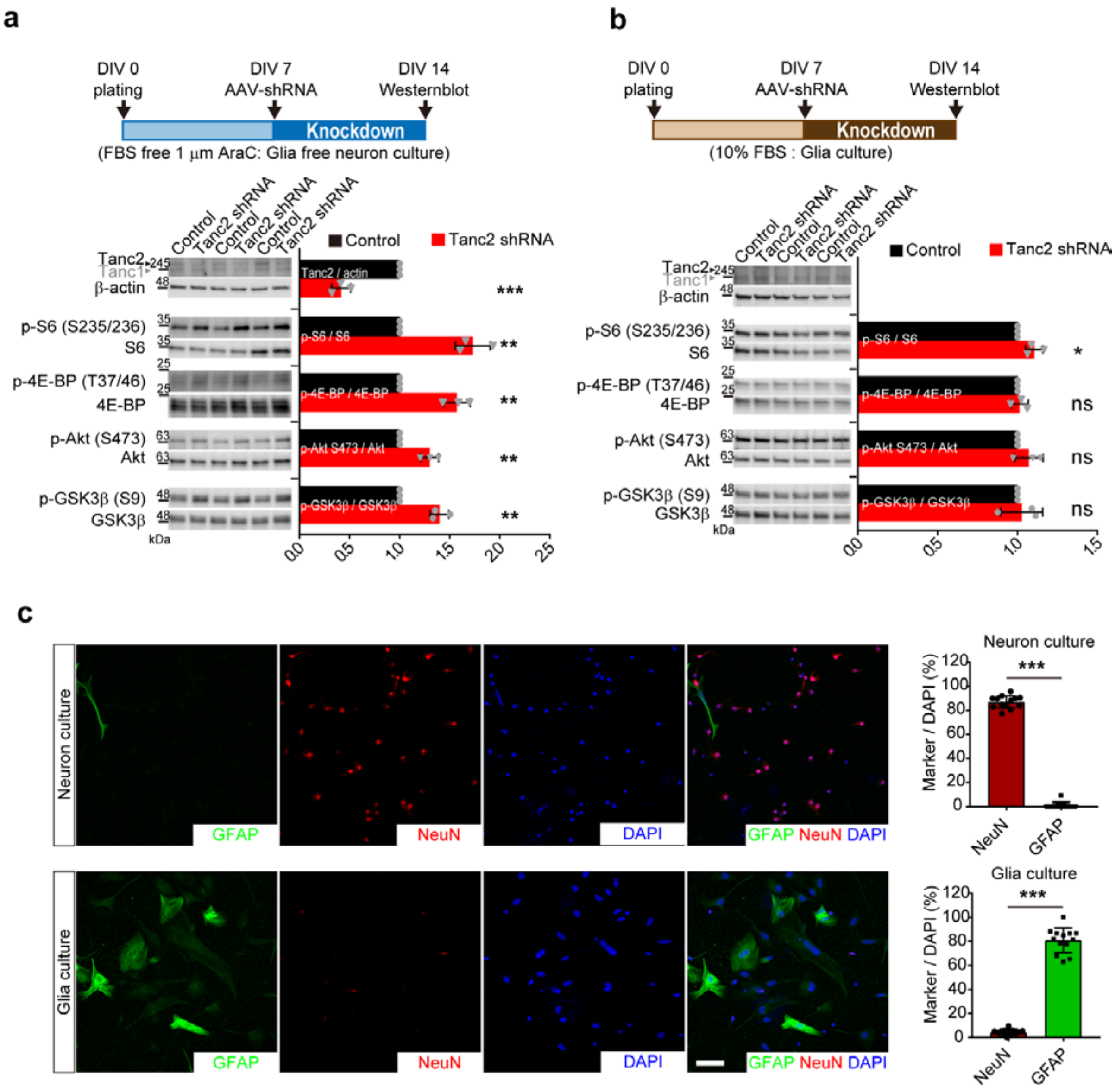

Supplementary Fig. 8. Acute Tanc2 knockdown in cultured mouse neurons, but not glial cells, leads to mTORC1/2 hyperactivity.

(a) Acute knockdown of Tanc2 in cultured mouse neurons by infection with AAV-shRNA (DIV7–14) leads to increased phosphorylation levels of S6 (S235/236), 4E-BP (T37/46), Akt (S473), and GSK3β (S9), indicative of mTORC1 and mTORC2 hyperactivity. Data: mean ± SD. (n = 3 independent experiments, $^{**}P < 0.01$, $^{***}P < 0.001$, Student's t-test).

(b) Acute knockdown of Tanc2 in cultured mouse glial cells by infection with AAV-shRNA (DIV7–14) does not affect the phosphorylation levels of S6 (S235/236), 4E-

BP (T37/46), Akt (S473) or GSK3β (S9), except for a small increase in p-S6, suggesting that Tanc2 mainly suppresses mTORC1/2 signaling in neurons rather than glial cells. Note that Tanc2 expression is much weaker relative to Tanc1. Data: mean ± SD. (n = 3 independent experiments, *P < 0.05, ns, not significant, Student's t-test).

(c) Control co-immunofluorescence staining showing that cultured neurons are positive for NeuN (neuronal marker) but not for GFAP (astrocytic marker) and that cultured glia are positive for GFAP but not for NeuN. DAPI was used for nuclear staining. Scale bar, 50 μm. Data: mean ± SEM. (n = 13 images for neuron and glial cultures, ***P < 0.001, Student's t-test).

See Source Data 1 for raw data values and Supplementary Table 1 for statistical details.

Supplementary Fig. 9

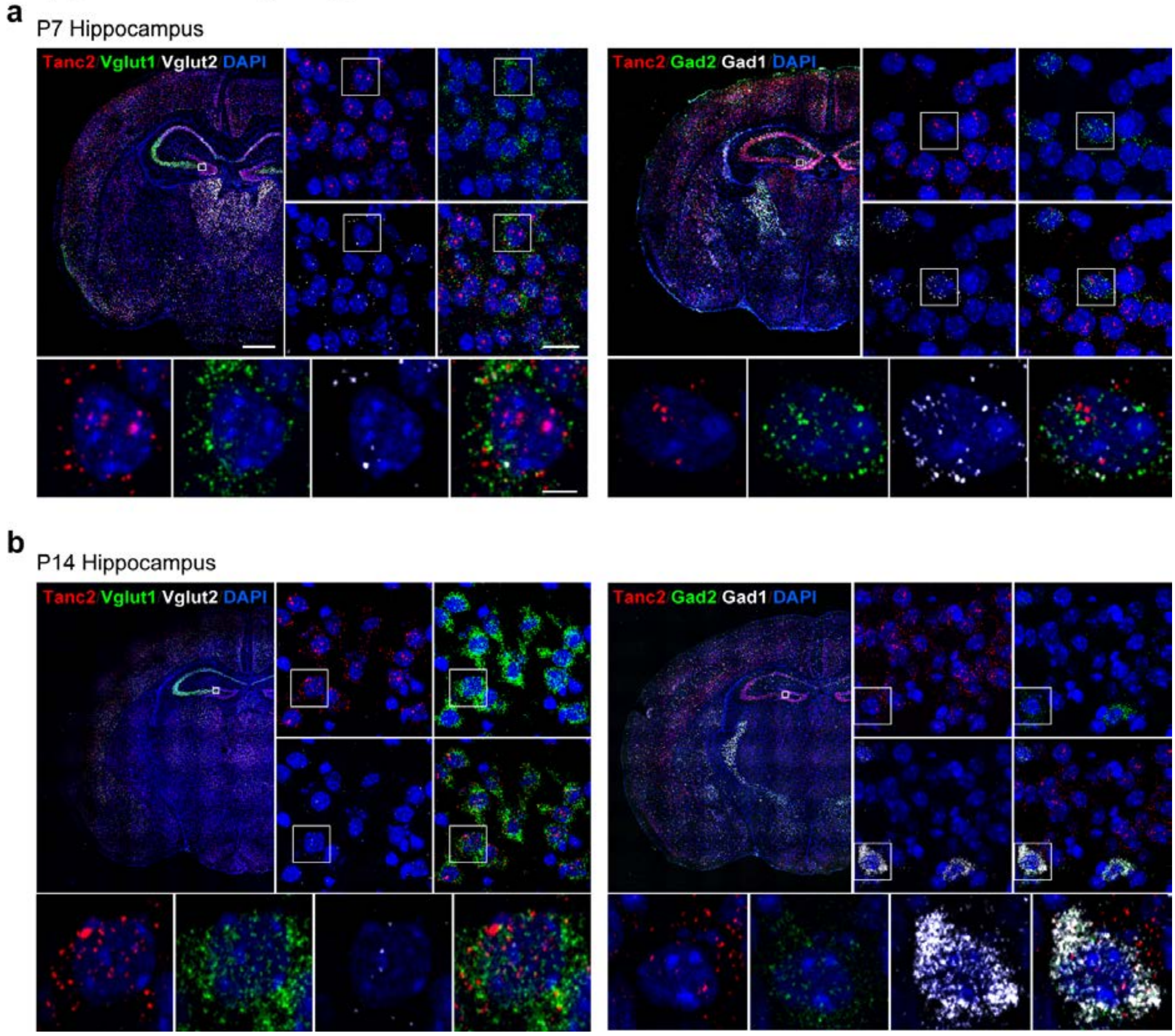

Supplementary Fig. 9. Tanc2 mRNA expression in both glutamatergic and GABAergic neurons in the mouse brain.

(a and b) Tanc2 expression is observed in both Vglut1/2-positive glutamatergic neurons and Gad1/2-positive GABAergic neurons in the cortical and hippocampal regions of the mouse brain at P7 and P14 (hippocampal examples are shown), as revealed by fluorescent in situ hybridization (FISH). Images in the bottom lines of each figure panel indicate enlarged single cells to clearly show the co-expression of Tanc2 with the indicated markers in the same cell. Scale bars, 500 µm (left), 20 µm (right), and 5 µm (bottom). Three independent experiments yielded similar results.

Supplementary Fig. 10

a

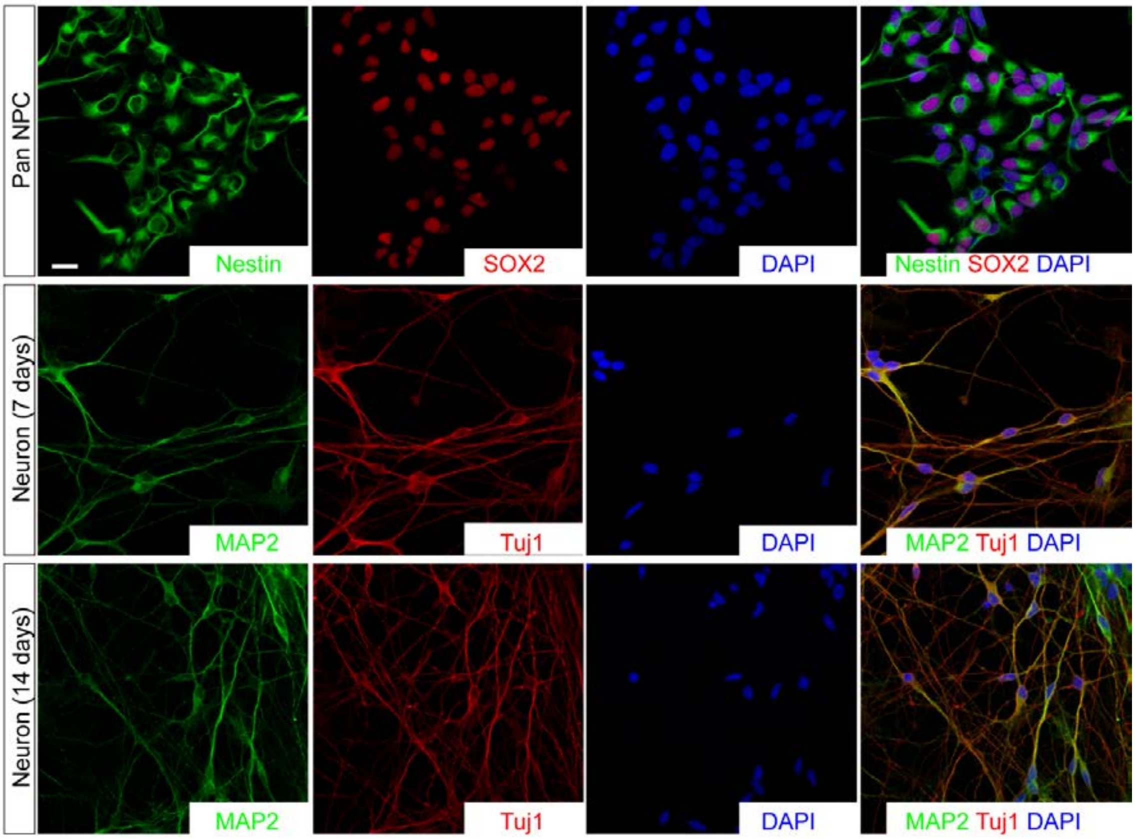

Supplementary Fig. 10. Characterization of human NPCs and neurons by immunofluorescence staining.

(a) Morphological characterization of pan-NPCs by immunofluorescence staining for Nestin and SOX2 (NPC markers) and pan-neurons at DIV7 and DIV14 by staining for MAP2 and Tuj1 (neuronal markers). Scale bar, 20 µm. Three independent experiments yielded similar results.

Supplementary table 1. Statictical analyis

Figure		Assay Performed	Comparison (unless stated otherwise, WT vs HT)	Sex	Age	n	Parameter (Unit)	Variable		Descriptive statistics (Average ± SE or SD)	Statistical test and significance			
											Statistical test	Significance		
1	a	Morris water maze		Male	2-5 months	WT = 20<br>HT = 20	Latency to escape (s)	Training	Day 1	WT = 52.20 ± 1.92<br>HT = 54.78 ± 2.44	Two-way ANOVA repeated measures Bonferroni's multiple comparison test	p = 0.9999, ns	Interaction: F(4, 76) = 2.39, p = 0.0581<br>Tanc2: F(1, 19) = 11.37, p = 0.0032<br>Time: F(4, 76) = 45.54, p < 0.0001	
									Day 2	WT = 38.55 ± 3.08<br>HT = 49.10 ± 2.28		p = 0.0180, *		
									Day 3	WT = 31.90 ± 3.80<br>HT = 46.33 ± 2.88		p = 0.0005, ***		
									Day 4	WT = 21.7 ± 3.01<br>HT = 38.21 ± 3.96		p = 0.0001, ***		
									Day 5	WT = 20.95 ± 2.58<br>HT = 29.56 ± 3.40		p = 0.0821, ns		
							Time spent in quadrant (s)	Probe test	Target	WT = 30.62 ± 2.42<br>HT = 19.86 ± 2.21	Two-way ANOVA repeated measures Bonferroni's multiple comparison test	p = 0.0001, ***	Interaction: F(3, 152) = 8.959, p < 0.0001<br>Tanc2: F(1, 152) = 2.607, p = 0.9986<br>Quadrant: F(3, 152) = 29.7, p < 0.0001	
									A Left	WT = 11.10 ± 1.30<br>HT = 13.13 ± 1.77		p = 0.9999, ns		
									A Right	WT = 10.54 ± 1.26<br>HT = 17.60 ± 1.97		p = 0.0278, *		
									Opposite	WT = 7.95 ± 1.38<br>HT = 9.86 ± 1.93		p = 0.9999, ns		
							Number of crossing (count)			WT = 3.90 ± 0.47<br>HT = 2.35 ± 0.50	Mann-Whitney U	p = 0.0128, *	U = 110	Two-tailed
							Swimming speed (cm/s)			WT = 14.29 ± 0.72<br>HT = 12.27 ± 0.89	Student's t-test	p = 0.0896, ns	t(38) = 1.745	Two-tailed
	b	Electrophysiology		Male	7-8 weeks	WT = 12 slices/ 5 mice<br>HT = 10 slices/ 5 mice	Hippocampus, HFS, 5 min , LTP (%)			WT = 152.3 ± 4.96<br>HT = 132.1 ± 5.02	Student's t-test	p = 0.0100, *	t(20) = 2.8440	Two-tailed
	c	Electrophysiology		Male	7-8 weeks	WT = 10 slices/ 3 mice<br>HT = 10 slices/ 3 mice	Input-output ratio		0.5	WT = 0.1508 ± 0.0151<br>HT = 0.1264 ± 0.0270	Two-way ANOVA repeated measures Bonferroni's multiple comparison test	p > 0.9999, ns	Interaction: F(10, 180) = 0.07672, p > 0.9999<br>Tanc2: F(1, 18) = 0.06271, p = 0.8051<br>Fiber Volley: F(10, 180) = 269.9, p < 0.0001	
									0.75	WT = 0.2782 ± 0.0145<br>HT = 0.2636 ± 0.0204		p > 0.9999, ns		
									1	WT = 0.3647 ± 0.0122<br>HT = 0.3648 ± 0.0143		p > 0.9999, ns		
									1.25	WT = 0.4580 ± 0.0113<br>HT = 0.4524 ± 0.0122		p > 0.9999, ns		
									1.5	WT = 0.5269 ± 0.0130<br>HT = 0.5303 ± 0.0151		p > 0.9999, ns		
									1.75	WT = 0.5905 ± 0.0195<br>HT = 0.5912 ± 0.0176		p > 0.9999, ns		
									2	WT = 0.6551 ± 0.0191<br>HT = 0.6542 ± 0.0226		p > 0.9999, ns		
									2.25	WT = 0.7085 ± 0.0242<br>HT = 0.7045 ± 0.0286		p > 0.9999, ns		
									2.5	WT = 0.7745 ± 0.0233<br>HT = 0.7660 ± 0.0332		p > 0.9999, ns		
									2.75	WT = 0.8250 ± 0.0284<br>HT = 0.8160 ± 0.0395		p > 0.9999, ns		
									3	WT = 0.8761 ± 0.0333<br>HT = 0.8695 ± 0.0411		p > 0.9999, ns		
	d					WT = 10 slices/ 3 mice<br>HT = 10 slices/ 3 mice	Hippocampus, PPR	Inter-pulse interval (ms)	25 msec	WT = 167.8 ± 3.77<br>HT = 159.8 ± 6.43	Two-way ANOVA repeated measures Bonferroni's multiple comparison test	p > 0.9999, ns	Interaction: F(5, 90) = 1.408, p = 0.2291<br>Tanc2: F(1, 18) = 0.01536, p = 0.9027<br>Time: F(5, 90) = 164.7, p < 0.0001	
									50 msec	WT = 173.2 ± 2.23<br>HT = 174.8 ± 3.93		p > 0.9999, ns		
									100 msec	WT = 141.3 ± 4.43<br>HT = 136.9 ± 2.46		p > 0.9999, ns		
									200 msec	WT = 110.7 ± 1.57<br>HT = 109.7 ± 2.41		p > 0.9999, ns		
									300 msec	WT = 110.7 ± 1.57<br>HT = 109.7 ± 2.41		p > 0.9999, ns		
	e	Electrophysiology		Male	4-5 weeks	WT = 12 slices/ 6 mice<br>HT = 12 slices/ 5 mice	Hippocampus, HFS, 5 min , LTP (%)			WT = 142.8 ± 4.52<br>HT = 132.4 ± 3.21	Student's t-test	p = 0.1041, ns	t(22) = 1.6950	Two-tailed
	f	Electrophysiology		Male	3-4 weeks	WT = 10 slices/ 4 mice<br>HT = 13 slices/ 5 mice	Input-output ratio		0.5	WT = 0.0620 ± 0.0062<br>HT = 0.0746 ± 0.0105	Two-way ANOVA repeated measures Bonferroni's multiple comparison test	p > 0.9999, ns	Interaction: F(6, 126) = 2.029, p = 0.0665<br>Tanc2: F(1, 21) = 1.551, p = 0.2267<br>Fiber Volley: F(6, 126) = 157, p < 0.0001	
									1	WT = 0.2561 ± 0.0695<br>HT = 0.2006 ± 0.0494		p > 0.9999, ns		
									1.5	WT = 0.4417 ± 0.0802<br>HT = 0.3295 ± 0.0654		p > 0.9999, ns		
									2	WT = 0.5944 ± 0.0933<br>HT = 0.4473 ± 0.0693		p > 0.9999, ns		
									3	WT = 0.8305 ± 0.107<br>HT = 0.6588 ± 0.0829		p > 0.9999, ns		
									4	WT = 1.0239 ± 0.1167<br>HT = 0.8215 ± 0.0871		p = 0.6209, ns		
									5	WT = 1.2235 ± 0.1273<br>HT = 0.9914 ± 0.0960		p = 0.3592, ns		
	g					WT = 11 slices/ 4 mice<br>HT = 13 slices/ 5 mice	Hippocampus, PPR	Inter-pulse interval (ms)	20 msec	WT = 167.8 ± 3.77<br>HT = 159.8 ± 6.43	Two-way ANOVA repeated measures Bonferroni's multiple comparison test	p = 0.6200, ns	Interaction: F(4, 88) = 1.388, p = 0.2447<br>Tanc2: F(1, 22) = 0.1803, p = 0.6752<br>Time: F(4, 88) = 149.3, p < 0.0001	
									50 msec	WT = 173.2 ± 2.23<br>HT = 174.8 ± 3.93		p > 0.9999, ns		
									100 msec	WT = 155.2 ± 2.08<br>HT = 159.4 ± 3.42		p > 0.9999, ns		
									200 msec	WT = 141.3 ± 4.43<br>HT = 136.9 ± 2.46		p > 0.9999, ns		
									500 msec	WT = 110.7 ± 1.57<br>HT = 109.7 ± 2.41		p > 0.9999, ns		
	h	Electrophysiology		Male	2-3 weeks	WT = 8 slices/ 6 mice<br>HT = 9 slices/ 5 mice	Hippocampus, 5 Hz, 5 min NMDAR LTD (%)			WT = 152.3 ± 4.96<br>HT = 132.1 ± 5.02	Student's t-test	p = 0.0134, *	t(15) = 2.8040	Two-tailed
	i	Electrophysiology			3-4 weeks	WT = 9 slices/ 5 mice<br>HT = 7 slices/ 4 mice	Hippocampus, DHPG (50 μ M), 5 min mGluR LTD (%)			WT = 67.6 ± 3.15<br>HT = 70.4 ± 4.11	Student's t-test	p = 0.6005, ns	t(14) = 0.5358	Two-tailed
	a	Western blotting		Male	P14	WT = 8<br>HT = 8	Protein levels normalized to α-tubulin (Fold change)	TANC2	Whole brain	WT = 1.19 ± 0.12 (SD)<br>HT = 0.80 ± 0.11 (SD)	Mann-Whitney U	p = 0.0281, *	U = 11	Two-tailed
						WT = 6<br>HT = 6		PSD-95		WT = 1.00 ± 0.19 (SD)<br>HT = 1.02 ± 0.19 (SD)	Student's t-test	p = 0.8455, ns	t(10) = 0.2	Two-tailed
						WT = 8<br>HT = 8		mTOR		WT = 1.00 ± 0.12 (SD)<br>HT = 1.27 ± 0.16 (SD)	Mann-Whitney U	p = 0.1304, ns	U = 17	Two-tailed
								p-mTOR		WT = 0.99 ± 0.13 (SD)<br>HT = 5.19 ± 1.34 (SD)	Student's t-test	p = 0.0076, **	t(14) = 3.1130	Two-tailed
						WT = 4<br>HT = 4		Akt		WT = 1.00 ± 0.05 (SD)<br>HT = 0.91 ± 0.02 (SD)	Student's t-test	p = 0.1814, ns	t(6) = 1.5120	Two-tailed
								p-Akt		WT = 1.00 ± 0.07 (SD)<br>HT = 2.81 ± 0.54 (SD)	Student's t-test	p = 0.0166, *	t(6) = 3.2890	Two-tailed
								GSK3β		WT = 1.00 ± 0.09 (SD)<br>HT = 1.06 ± 0.04 (SD)	Student's t-test	p = 0.5677, ns	t(6) = 0.6044	Two-tailed
								p-GSK3β		WT = 1.00 ± 0.14 (SD)<br>HT = 1.80 ± 0.16 (SD)	Student's t-test	p = 0.0109, *	t(6) = 0.3636	Two-tailed
						WT = 6<br>HT = 6		PKCa		WT = 1.00 ± 0.2 (SD)<br>HT = 1.06 ± 0.04 (SD)	Student's t-test	p = 0.8332, ns	t(10) = 0.2162	Two-tailed
								p-PKCa		WT = 1.00 ± 0.14 (SD)<br>HT = 1.80 ± 0.16 (SD)	Student's t-test	p = 0.1291, ns	t(10) = 1.654	Two-tailed
						WT = 4<br>HT = 4		S6		WT = 0.99 ± 0.03 (SD)<br>HT = 1.08 ± 0.05 (SD)	Student's t-test	p = 0.2486, ns	t(6) = 1.2780	Two-tailed
								p-S6		WT = 1.00 ± 0.15 (SD)<br>HT = 1.10 ± 0.17 (SD)	Mann-Whitney U	p = 0.6857, ns	U = 6	Two-tailed
								4EBP		WT = 1.00 ± 0.15 (SD)<br>HT = 1.46 ± 0.05 (SD)	Student's t-test	p = 0.0340, *	t(6) = 2.7340	Two-tailed
								p-4EBP		WT = 1.00 ± 0.15 (SD)<br>HT = 1.77 ± 0.06 (SD)	Mann-Whitney U	p = 0.0286, *	U = 0	Two-tailed
	b	Western blotting		Male	P14	WT = 4<br>HT = 4	Protein levels normalized to α-tubulin (Fold change)	ERK1/2	Whole brain	WT = 1.00 ± 0.14 (SD)<br>HT = 0.93 ± 0.14 (SD)	Student's t-test	p = 0.7544, ns	t(6) = 0.3276	Two-tailed
								p-ERK1/2		WT = 0.99 ± 0.10 (SD)<br>HT = 1.26 ± 0.21 (SD)	Mann-Whitney U	p = 0.3429, ns	U = 4	Two-tailed
								p38		WT = 1.06 ± 0.12 (SD)<br>HT = 1.10 ± 0.04 (SD)	Student's t-test	p = 0.7909, ns	t(6) = 0.2772	Two-tailed
								p-p38		WT = 1.00 ± 0.05 (SD)<br>HT = 0.93 ± 0.15 (SD)	Student's t-test	p = 0.7084, ns	t(6) = 0.3923	Two-tailed
								TSC1		WT = 1.00 ± 0.02 (SD)<br>HT = 0.99 ± 0.12 (SD)	Student's t-test	p = 0.9413, ns	t(6) = 0.0767	Two-tailed
								TSC2		WT = 1.00 ± 0.07 (SD)<br>HT = 0.76 ± 0.05 (SD)	Student's t-test	p = 0.0286, *	t(6) = 2.8640	Two-tailed
								PTEN		WT = 1.00 ± 0.13 (SD)<br>HT = 1.43 ± 0.20 (SD)	Student's t-test	p = 0.1276, ns	t(6) = 1.7670	Two-tailed
								p-PTEN		WT = 1.00 ± 0.15 (SD)<br>HT = 1.10 ± 0.16 (SD)	Student's t-test	p = 0.6444, ns	t(6) = 0.4858	Two-tailed
								PI3K		WT = 1.00 ± 0.07 (SD)<br>HT = 1.04 ± 0.05 (SD)	Student's t-test	p = 0.6791, ns	t(6) = 0.4346	Two-tailed
								p-PI3K		WT = 1.31 ± 0.41 (SD)<br>HT = 1.17 ± 0.26 (SD)	Student's t-test	p = 0.9286, ns	t(6) = 0.0933	Two-tailed
	c	Western blotting		Male	P28	WT = 4<br>HT = 4	Protein levels normalized to β-actin (Fold change)	TANC2	Whole brain	WT = 1.00 ± 0 (SD)<br>HT = 0.48 ± 0.13 (SD)	Student's t-test	p = 0.0003, ***	t(6) = 7.351	Two-tailed
								PSD-95		WT = 1.19 ± 0.12 (SD)<br>HT = 0.80 ± 0.11 (SD)		p = 0.0281, *	U = 11	Two-tailed
								mTOR		WT = 1.00 ± 0 (SD)<br>HT = 1.09 ± 0.24 (SD)		p = 0.4522, ns	t(6) = 0.8038	Two-tailed
								p-mTOR		WT = 1.00 ± 0 (SD)<br>HT = 1.08 ± 0.20 (SD)		p = 0.4681, ns	t(6) = 0.7743	Two-tailed
								Akt		WT = 1.00 ± 0 (SD)<br>HT = 0.92 ± 0.17 (SD)		p = 0.4369, ns	t(6) = 0.8328	Two-tailed
								p-Akt		WT = 1.00 ± 0 (SD)<br>HT = 1.01 ± 0.19 (SD)		p = 0.8585, ns	t(6) = 0.186	Two-tailed
								GSK3β		WT = 1.00 ± 0 (SD)<br>HT = 1.01 ± 0.35 (SD)		p = 0.9271, ns	t(6) = 0.0953	Two-tailed
								p-GSK3β		WT = 1.00 ± 0 (SD)<br>HT = 1.11 ± 0.46 (SD)		p = 0.6413, ns	t(6) = 0.4904	Two-tailed
								PKCa		WT = 1.00 ± 0.09 (SD)<br>HT = 1.02 ± 0.19 (SD)		p = 0.5677, ns	t(6) = 0.6044	Two-tailed

2								p-PKCa		WT = 1.00 ± 0.16 (SD)<br>HT = 1.14 ± 0.14 (SD)		p = 0.0109, *	t(6) = 0.3.636	Two-tailed
								S6		WT = 1.00 ± 0 (SD)<br>HT = 1.04 ± 0.12 (SD)		p = 0.4753, ns	t(6) = 0.7614	Two-tailed
								p-S6		WT = 1.00 ± 0 (SD)<br>HT = 0.92 ± 0.15 (SD)		p = 0.3993, ns	t(6) = 0.907	Two-tailed
								4EBP		WT = 1.00 ± 0 (SD)<br>HT = 0.92 ± 0.19 (SD)		p = 0.4761, ns	t(6) = 0.7599	Two-tailed
								p-4EBP		WT = 1.00 ± 0 (SD)<br>HT = 0.97 ± 0.26 (SD)		p = 0.8519, ns	t(6) = 0.195	Two-tailed
	d	Western blotting		Male	P52	WT = 6<br>HT = 6	Protein levels normalized to β-actin (Fold change)	TANC2	Whole brain	WT = 1.00 ± 0 (SD)<br>HT = 0.42 ± 0.08 (SD)	Student's t-test	p < 0.0001, ***	t(10) = 16.87	Two-tailed
								PSD-95		WT = 1.00 ± 0 (SD)<br>HT = 0.97 ± 0.21 (SD)		p = 0.8255, ns	t(10) = 0.2264	Two-tailed
								mTOR		WT = 1.00 ± 0 (SD)<br>HT = 1.04 ± 0.21 (SD)		p = 0.6558, ns	t(10) = 0.4594	Two-tailed
								p-mTOR		WT = 1.00 ± 0 (SD)<br>HT = 0.92 ± 0.17 (SD)		p = 0.3062, ns	t(10) = 1.078	Two-tailed
								Akt		WT = 1.00 ± 0 (SD)<br>HT = 0.13 ± 0.27 (SD)		p = 0.26, ns	t(10) = 1.194	Two-tailed
								p-Akt		WT = 1.00 ± 0 (SD)<br>HT = 0.83 ± 0.38 (SD)		p = 0.3275, ns	t(10) = 1.029	Two-tailed
								GSK3β		WT = 1.00 ± 0 (SD)<br>HT = 0.98 ± 0.11 (SD)		p = 0.7703, ns	t(10) = 0.3001	Two-tailed
								p-GSK3β		WT = 1.00 ± 0 (SD)<br>HT = 0.97 ± 0.05 (SD)		p = 0.2735, ns	t(10) = 1.159	Two-tailed
								PKCa		WT = 1.00 ± 0.09 (SD)<br>HT = 0.95 ± 0.15 (SD)		p = 0.5353, ns	t(10) = 0.6421	Two-tailed
								p-PKCa		WT = 1.00 ± 0.14 (SD)<br>HT = 0.92 ± 0.13 (SD)		p = 0.2121, ns	t(10) = 1.333	Two-tailed
								S6		WT = 1.00 ± 0 (SD)<br>HT = 0.95 ± 0.15 (SD)		p = 0.4457, ns	t(10) = 0.7939	Two-tailed
								p-S6		WT = 1.00 ± 0 (SD)<br>HT = 0.85 ± 0.32 (SD)		p = 0.3054, ns	t(10) = 1.08	Two-tailed
								4EBP		WT = 1.00 ± 0 (SD)<br>HT = 0.93 ± 0.19 (SD)		p = 0.4366, ns	t(10) = 0.8104	Two-tailed
								p-4EBP		WT = 1.00 ± 0 (SD)<br>HT = 0.86 ± 0.07 (SD)		p = 0.0017, **	t(10) = 4.256	Two-tailed
	f	Western blotting	GFP vs GFP-cre	male	P14	WT = 6<br>HT = 6	Protein levels normalized to β-actin (Fold change)	TANC2		GFP = 1.00 ± 0 (SD)<br>GFP-Cre = 0.55 ± 0.08 (SD)	Student's t-test	p = 0.0006, ***	t(10) = 4.972	Two-tailed
								mTOR		GFP = 1.00 ± 0 (SD)<br>GFP-Cre = 1.06 ± 0.06 (SD)		p = 0.3207, ns	t(10) = 1.045	Two-tailed
								p-mTOR		GFP = 1.00 ± 0 (SD)<br>GFP-Cre = 1.53 ± 0.20 (SD)		p = 0.0242, *	t(10) = 2.652	Two-tailed
								Akt		GFP = 1.00 ± 0 (SD)<br>GFP-Cre = 0.96 ± 0.07 (SD)		p = 0.6930, ns	t(10) = 0.4064	Two-tailed
								p-Akt		GFP = 1.00 ± 0 (SD)<br>GFP-Cre = 1.58 ± 0.12 (SD)		p = 0.0007, ***	t(10) = 4.837	Two-tailed
								GSK3β		GFP = 1.00 ± 0 (SD)<br>GFP-Cre = 1.01 ± 0.04 (SD)		p = 0.8213, ns	t(10) = 0.2319	Two-tailed
								p-GSK3β		GFP = 1.00 ± 0 (SD)<br>GFP-Cre = 1.67 ± 0.14 (SD)		p = 0.0008, ***	t(10) = 4.725	Two-tailed
								S6		GFP = 1.00 ± 0 (SD)<br>GFP-Cre = 0.78 ± 0.07 (SD)		p = 0.0215, *	t(10) = 2.72	Two-tailed
								p-S6		GFP = 1.00 ± 0 (SD)<br>GFP-Cre = 1.68 ± 0.28 (SD)		p = 0.0364, *	t(10) = 2.414	Two-tailed
								4EBP		GFP = 1.00 ± 0 (SD)<br>GFP-Cre = 1.40 ± 0.14 (SD)		p = 0.0191, *	t(10) = 2.791	Two-tailed
								p-4EBP		GFP = 1.00 ± 0 (SD)<br>GFP-Cre = 2.21 ± 0.40 (SD)		p = 0.0133, *	t(10) = 3.004	Two-tailed
	h	Western blotting	GFP vs GFP-cre	male	P28	WT = 6<br>HT = 6	Protein levels normalized to β-actin (Fold change)	TANC2		GFP = 1.00 ± 0 (SD)<br>GFP-Cre = 0.48 ± 0.05 (SD)	Student's t-test	p = 0.0006, ***	t(10) = 23.95	Two-tailed
								mTOR		GFP = 1.00 ± 0 (SD)<br>GFP-Cre = 1.1 ± 0.23 (SD)		p = 0.2796, ns	t(10) = 1.143	Two-tailed
								p-mTOR		GFP = 1.00 ± 0 (SD)<br>GFP-Cre = 1.06 ± 0.17 (SD)		p = 0.3743, ns	t(10) = 0.93	Two-tailed
								Akt		GFP = 1.00 ± 0 (SD)<br>GFP-Cre = 0.99 ± 0.13 (SD)		p = 0.9727, ns	t(10) = 0.0351	Two-tailed
								p-Akt		GFP = 1.00 ± 0 (SD)<br>GFP-Cre = 1.13 ± 0.32 (SD)		p = 0.3343, ns	t(10) = 1.014	Two-tailed
								GSK3β		GFP = 1.00 ± 0 (SD)<br>GFP-Cre = 1.04 ± 0.15 (SD)		p = 0.548, ns	t(10) = 0.6218	Two-tailed
								p-GSK3β		GFP = 1.00 ± 0 (SD)<br>GFP-Cre = 0.97 ± 0.34 (SD)		p = 0.8376, ns	t(10) = 0.2104	Two-tailed
								S6		GFP = 1.00 ± 0 (SD)<br>GFP-Cre = 1.29 ± 0.13 (SD)		p = 0.0003, ***	t(10) = 5.379	Two-tailed
								p-S6		GFP = 1.00 ± 0 (SD)<br>GFP-Cre = 1.54 ± 0.78 (SD)		p = 0.1198, ns	t(10) = 1.701	Two-tailed
								4EBP		GFP = 1.00 ± 0 (SD)<br>GFP-Cre = 0.69 ± 0.12 (SD)		p = 0.0001, ***	t(10) = 5.947	Two-tailed
								p-4EBP		GFP = 1.00 ± 0 (SD)<br>GFP-Cre = 0.64 ± 0.13 (SD)		p < 0/0001, ***	t(10) = 6.432	Two-tailed
3	b	Electorphysiology (Local field current clamp)	WT vehicle vs HT vehicle vs WT rapamycin vs HT rapamycin [5 mg/kg]	male	7-8 weeks	WT vehicle = 10 slices/ 5 mice<br>WT rapamycin = 14 slices/ 6 mice<br>HT vehicle = 12 slices/ 5 mice<br>HT rapamycin = 15 slices/ 7 mice	Hippocampus, 100 Hz, 5min HFS LTP (%)			WT vehicle = 158 ± 6.28<br>WT rapamycin = 159 ± 5.79<br>HT vehicle = 130 ± 3.33<br>HT rapamycin = 151 ± 3.76	Two-way ANOVA<br>Bonferroni's multiple comparison test	WT vehicle vs HT vehicle, p = 0.0025; WT vehicle vs WT rapamycin, ns; HT vehicle vs HT rapamycin, p = 0.0171; WT vehicle vs HT rapamycin, ns	Interaction F(1, 47) = 4.242, p = 0.0450 Tanc2 F(1, 47) = 13.49, p = 0.0006<br>Treatment F(1, 47) = 5.028, p = 0.0297	
	c	Morris water maze			2-4 months	WT vehicle = 16 mice<br>WT rapamycin = 15 mice<br>HT vehicle = 11 mice<br>HT rapamycin = 15 mice	Latency to escape (s)	Training	Day 1	WT vehicle = 51.56 ± 2.27<br>WT rapamycin = 50.73 ± 2.13<br>HT vehicle = 54.18 ± 2.28<br>HT rapamycin = 52.16 ± 2.36	Two-way ANOVA repeated measures<br>Bonferroni's multiple comparison test	WT vehicle vs HT vehicle, ns; WT vehicle vs WT rapamycin, ns; HT vehicle vs HT rapamycin, ns; WT vehicle vs HT rapamycin, ns	Interaction F(12, 212) = 1.995, p = 0.0260<br>Treatment F(3, 53) = 3.274, p < 0.0281<br>Time F(4, 212) = 50.47, p <0.0001	
									Day 2	WT vehicle = 35.27 ± 2.95<br>WT rapamycin = 38.67 ± 14.34<br>HT vehicle = 40.55 ± 5.43<br>HT rapamycin = 44.64 ± 3.08		WT vehicle vs HT vehicle, ns; WT vehicle vs WT rapamycin, ns; HT vehicle vs HT rapamycin, ns; WT vehicle vs HT rapamycin, ns		
									Day 3	WT vehicle = 28.27 ± 3.86<br>WT rapamycin = 31.64 ± 4.31<br>HT vehicle = 49.15 ± 2.73<br>HT rapamycin = 31.49 ± 2.91		WT vehicle vs HT vehicle, p = 0.0005; WT vehicle vs WT rapamycin, ns; HT vehicle vs HT rapamycin, p = 0.0030; WT vehicle vs HT rapamycin, ns		
									Day 4	WT vehicle = 24.98 ± 3.66<br>WT rapamycin = 28.16 ± 3.19<br>HT vehicle = 33.10 ± 4.45<br>HT rapamycin = 27.31 ± 3.93		WT vehicle vs HT vehicle, ns; WT vehicle vs WT rapamycin, ns; HT vehicle vs HT rapamycin, ns; WT vehicle vs HT rapamycin, ns		
									Day 5	WT vehicle = 22.96 ± 3.41<br>WT rapamycin = 23.07 ± 2.01<br>HT vehicle = 38.7 ± 4.23<br>HT rapamycin = 23.38 ± 2.93		WT vehicle vs HT vehicle, p = 0.0148; WT vehicle vs WT rapamycin, ns; HT vehicle vs HT rapamycin, p = 0.0116; WT vehicle vs HT rapamycin, ns		
							Number of crossing (count)	Probe test	Day 5	WT vehicle = 3.13 ± 0.47<br>WT rapamycin = 2.20 ± 0.40<br>HT vehicle = 1.10 ± 0.41<br>HT rapamycin = 2.73 ± 0.36	One-way ANOVA<br>Bonferroni's multiple comparison test	WT vehicle vs HT vehicle, p = 0.0269; WT vehicle vs WT rapamycin, ns; HT vehicle vs HT rapamycin, ns; WT vehicle vs HT rapamycin, ns	Interaction F(3, 53) = 3.210, p = 0.0302	
							Swimming speed (cm/s)			WT vehicle = 18.91 ± 0.72<br>WT rapamycin = 19.76 ± 1.11<br>HT vehicle = 16.32 ± 0.70<br>HT rapamycin = 18.30 ± 0.69	One-way ANOVA<br>Bonferroni's multiple comparison test	WT vehicle vs HT vehicle, ns; WT vehicle vs WT rapamycin, ns; HT vehicle vs HT rapamycin, ns; WT vehicle vs HT rapamycin, ns	Interaction F(3, 53) = 2.604, p = 0.0614	
	d	Open field test	WT vehicle vs HT vehicle vs WT rapamycin vs HT rapamycin [5 mg/kg]	Male	7-8 weeks	WT vehicle = 15 mice<br>WT rapamycin = 16 mice<br>HT vehicle = 12 mice<br>HT rapamycin = 14 mice	Total distance moved (m)		60 minutes	WT vehicle = 159.65 ± 4.41<br>WT rapamycin = 140.70 ± 4.86<br>HT vehicle = 184.37 ± 7.56<br>HT rapamycin = 149.08 ± 6.19	Two-way ANOVA<br>Bonferroni's multiple comparison test	WT vehicle vs HT vehicle, p = 0.0243; WT vehicle vs WT rapamycin, ns; HT vehicle vs HT rapamycin, p = 0.0006; WT rapamycin vs HT rapamycin, ns; WT vehicle vs HT rapamycin, ns	Interaction F(1, 53) = 2.082, p = 0.1549 Tanc2 F(1, 53) = 8.553, p = 0.0051<br>Treatment F(1, 53) = 22.97, p < 0.0001	
	e	EPM				WT vehicle = 16 mice<br>WT rapamycin = 19 mice<br>HT vehicle = 12 mice<br>HT rapamycin = 15 mice	Time spent in arm (s)	Open arm		WT vehicle = 108.08 ± 6.93<br>WT rapamycin = 109.38 ± 6.99<br>HT vehicle = 141.35 ± 11.65<br>HT rapamycin = 106.86 ± 11.71	One-way ANOVA<br>Bonferroni's multiple comparison test	WT vehicle vs HT vehicle, ns; WT vehicle vs WT rapamycin, ns; WT rapamycin vs HT vehicle, ns; HT vehicle vs HT rapamycin, ns; WT rapamycin vs HT rapamycin, ns; WT vehicle vs HT rapamycin, ns	Interaction F(3, 58) = 2.107, p = 0.1092	
								Closed arm		WT vehicle = 355.08 ± 9.76<br>WT rapamycin = 367.09 ± 10.01<br>HT vehicle = 308.74 ± 12.5<br>HT rapamycin = 365.45 ± 18.76		WT vehicle vs HT vehicle, ns; WT vehicle vs WT rapamycin, ns; WT rapamycin vs HT vehicle, p = 0.0339; HT vehicle vs HT rapamycin, ns; WT rapamycin vs HT rapamycin, ns; WT vehicle vs HT rapamycin, ns	Interaction F(3, 58) = 3.259, p = 0.0278	
								Closed arm		WT vehicle = 355.08 ± 9.76<br>WT rapamycin = 367.09 ± 10.01<br>HT vehicle = 308.74 ± 12.5<br>HT rapamycin = 365.45 ± 18.76	One-way ANOVA Tukey's multiple comparison test	WT vehicle vs HT vehicle, ns; WT vehicle vs WT rapamycin, ns; WT rapamycin vs HT vehicle, p = 0.0339; HT vehicle vs HT rapamycin, p = 0483; WT rapamycin vs HT rapamycin, ns; WT vehicle vs HT rapamycin, ns	Interaction F(3, 58) = 3.259, p = 0.0278	
5	a	Western blotting				n=3	p-mTOR/mTOR levels in HEK293 cells normalized to untreated p-mTOR/mTOR levels	p-mTOR/mTOR	Untreat	1.01 ± 0.07 (SD)	One-way ANOVA<br>Bonferroni's multiple comparison test		F(7, 16) = 22.88, p < 0.0001	
									Flag-Empty	0.85 ± 0.09 (SD)		p = 0.7274, ns		
									Flag-hTanc2a	0.53 ± 0.05 (SD)		p = 0.0005, ***		
									Flag-hTanc2b	0.74 ± 0.06 (SD)		p = 0.0624, ns		
									Flag-hTanc2c	0.94 ± 0.04 (SD)		p > 0.9999, ns		
									Rapamycin 1 nM	0.71 ± 0.08 (SD)		p = 0.0342, *		
									Rapamycin 10 nM	0.31 ± 0.03 (SD)		p < 0.0001, ***		
									Rapamycin 100 nM	0.14 ± 0.01 (SD)		p < 0.0001, ***		
							p-S6/S6 levels in HEK293 cells normalized to untreated p-S6/S6 levels	p-S6/S6	Untreat	0.96 ± 0.02 (SD)			F(7, 16) = 27.35, p < 0.0001	
									Flag-Empty	1.20 ± 0.12 (SD)		p = 0.6487, ns		
									Flag-hTanc2a	0.41 ± 0.09 (SD)		p = 0.0054, **		
									Flag-hTanc2b	0.94 ± 0.18 (SD)		p > 0.9999, ns		
									Flag-hTanc2c	0.89 ± 0.11 (SD)		p > 0.9999, ns		
									Rapamycin 1 nM	0.08 ± 0.03 (SD)		p < 0.0001, ***		
									Rapamycin 10 nM	0.01 ± 0.004 (SD)		p < 0.0001, ***		
									Rapamycin 100 nM	0.003 ± 0.003 (SD)		p < 0.0001, ***		
	b	In vitro kinase assay (Western blotting)				n=3	Protein levels normalized to mTORC only (Fold change)		mTORC	1.00 ± 0 (SD)	One-way ANOVA<br>Bonferroni's multiple comparison test	mTORC S6K ATP vs mTORC S6K ATP Tanc2, p > 0.9999, ns	F(4, 10) = 1.352, p = 0.3172	
									mTORC ATP	0.99 ± 0.16 (SD)				
									mTORC S6K	0.76 ± 0.12 (SD)				
									mTORC S6K ATP	1.89 ± 0.74 (SD)				
									mTORC S6K ATP Tanc2	1.03 ± 0.31 (SD)				
						n=3	Protein levels normalized to mTORC, S6K, and ATP (Fold change)		mTORC S6K	0.01 ± 0.004 (SD)	One-way ANOVA<br>Bonferroni's multiple comparison test	mTORC S6K vs mTORC S6K ATP, p=0.0001; mTORC S6K ATP vs mTORC S6K ATP Tanc2, p = 0.0473, *	F(2, 6) = 51.47, p = 0.0002	
									mTORC S6K ATP	1.00 ± 0.00 (SD)				
									mTORC S6K ATP Tanc2	0.66 ± 0.21 (SD)				
	c	In vitro kinase assay				n=3	Protein levels normalized to mTORC only (Fold change)		mTORC	1.00 ± 0 (SD)	One-way ANOVA<br>Bonferroni's multiple comparison test	mTORC Akt ATP vs mTORC Akt ATP Tanc2, p > 0.9999, ns	F(4, 10) = 0.6426, p = 0.6444	
									mTORC ATP	1.21 ± 0.38 (SD)				
									mTORC Akt	1.19 ± 0.19 (SD)				
									mTORC Akt ATP	1.27 ± 0.47 (SD)				

	[illegible]	(Western blotting)							mTORC Akt ATP Tanc2	1.39 ± 0.26 (SD)				
						n=3	Protein levels normalized to mTORC, S6K, and ATP (Fold change)		mTORC Akt	0.41 ± 0.05 (SD)	One-way ANOVA Bonferroni's multiple comparison test	mTORC Akt vs mTORC Akt ATP, p<0.0001; mTORC Akt ATP vs mTORC Akt ATP Tanc2, p = 0.0006, ***	F(2, 6) = 204.6, p < 0.0001	
									mTORC Akt ATP	1.00 ± 0.00 (SD)				
									mTORC Akt ATP Tanc2	0.76 ± 0.02 (SD)				
6	a	Confocal microscopy				n=24 cells	Pearson's correlation coefficient between CFP-Tanc2 and YFP-mTOR		+serum	0.27 ± 0.02	One-way ANOVA Bonferroni's multiple comparison test	+serum vs -serum 4 hr, p < 0.0001;+serum vs +serum 4hr, p < 0.0001; +serum vs +serum 24hr, p > 0.9999; -serum 4 hr vs +serum 4 hr, p = 0.8761; -serum 4 hr vs +serum 24 hr, p < 0.0001; +serum 4hr vs +serum 24hr, p < 0.0001	F(3, 92) = 60.12, p < 0.0001	
									-serum 4 hr	0.66 ± 0.02				
									+serum 4 hr	0.61 ± 0.02				
									+serum 24 hr	0.32 ± 0.02				
	b	Confocal microscopy				n=24 cells	Pearson's correlation coefficient between CFP-Tanc2 and YFP-mTOR	Vehicle	+serum	0.23 ± 0.02	Student's t-test	p < 0.0001, ***	t(46) = 11.17	Two-tailed
									-serum 4 hr	0.65 ± 0.02				
								Rapamycin	+serum	0.29 ± 0.02	Student's t-test	p = 0.5988, ns	t(46) = 0.5299	Two-tailed
									-serum 4 hr	0.31 ± 0.02				
	c	Coimmunoprecipitation (Western blotting)				n=4	mTOR/Tanc2 ratio in HEK293T cells (Fold change)		Serum +	1.00 ± 0.00 (SD)	Student's t-test	p = 0.0158, *	t(6) = 3.329	Two-tailed
									Serum -	2.15 ± 0.34 (SD)				
	d	Coimmunoprecipitation (Western blotting)				n=3	mTOR/Tanc2 ratio in HEK293T cells (Fold change)	Vehicle	+serum	1.00 ± 0.00 (SD)	Student's t-test	p < 0.0001, ***	t(4) = 29.66	Two-tailed
									-serum 4 hr	2.18 ± 0.04 (SD)				
								Rapamycin	+serum	1.35 ± 0.20 (SD)	Student's t-test	p = 0.5988, ns	t(4) = 29.66	Two-tailed
									-serum 4 hr	1.46 ± 0.24 (SD)				
	e	Coimmunoprecipitation using P2 fraction (Western blotting)			P13-14	n=4	Ratio (Fold change)	mTOR/Tanc	0 hr	1.00 ± 0.00 (SD)	One-way ANOVA Bonferroni's multiple comparison test		F(2, 6) = 7.748, p = 0.011	
									0.5 hr	0.73 ± 0.06 (SD)		p = 0.0241, *		
									1 hr	0.69 ± 0.08 (SD)		p = 0.011, *		
								PSD-95/Tanc	0 hr	1.00 ± 0.00 (SD)	One-way ANOVA Bonferroni's multiple comparison test		F(2, 6) = 1.319, p = 0.3144	
									0.5 hr	1.03 ± 0.15 (SD)		p > 0.9999, ns		
									1 hr	1.20 ± 0.07 (SD)		p = 0.3317, ns		
						n=5		Tanc/mTOR	0 hr	1.00 ± 0.00 (SD)	One-way ANOVA Bonferroni's multiple comparison test		F(2, 12) = 5.367, p = 0.0216	
									0.5 hr	0.79 ± 0.25 (SD)		p = 0.1345, ns		
									1 hr	0.67 ± 0.10 (SD)		p = 0.0140, *		
								PSD-95/mTOR	0 hr	1.00 ± 0.00 (SD)	One-way ANOVA Bonferroni's multiple comparison test		F(2, 12) = 0.9637, p = 0.4091	
									0.5 hr	1.04 ± 0.06 (SD)		p > 0.9999, ns		
									1 hr	0.12 ± 0.09 (SD)		p = 0.3092, ns		
7	b	Western blotting			DIV 14	n=4	Ratio (Fold change)	Tanc2 / actin	Control	1.00 ± 0.00 (SD)	One-way ANOVA Bonferroni's multiple comparison test		F(4, 15) = 25.89, p < 0.0001	
									Tanc2 shRNA	0.31 ± 0.13 (SD)		p = 0.0002, ***		
									Deptor shRNA	1.12 ± 0.17 (SD)		p > 0.9999, ns		
									Tanc2 & Deptor shRNA	0.23 ± 0.16 (SD)		p < 0.0001, ***		
									Tanc1 shRNA	1.03 ± 0.25 (SD)		p > 0.9999, ns		
								Deptor / actin	Control	1.00 ± 0.00 (SD)			F(4, 15) = 16.16, p < 0.0001	
									Tanc2 shRNA	0.98 ± 0.17 (SD)		p > 0.9999, ns		
									Deptor shRNA	0.41 ± 0.08 (SD)		p = 0.0033, **		
									Tanc2 & Deptor shRNA	0.33 ± 0.12 (SD)		p = 0.0012, **		
									Tanc1 shRNA	1.25 ± 0.38 (SD)		p = 0.3681, ns		
								Tanc1 / actin	Control	1.00 ± 0.00 (SD)			F(4, 15) = 20.45, p < 0.0001	
									Tanc2 shRNA	1.10 ± 0.08 (SD)		p > 0.9999, ns		
									Deptor shRNA	1.10 ± 0.17 (SD)		p > 0.9999, ns		
									Tanc2 & Deptor shRNA	1.22 ± 0.22 (SD)		p = 0.14, ns		
									Tanc1 shRNA	0.43 ± 0.07 (SD)		p = 0.0001, ***		
								p-S6 / S6	Control	1.00 ± 0.00 (SD)			F(4, 15) = 6,478 p = 0.0031	
									Tanc2 shRNA	2.29 ± 0.57 (SD)		p = 0.0091, **		
									Deptor shRNA	1.31 ± 0.33 (SD)		p = 0.8660, ns		
									Tanc2 & Deptor shRNA	2.28 ± 0.63 (SD)		p = 0.0096, **		
									Tanc1 shRNA	1.95 ± 0.47 (SD)		p = 0.0426, *		
								p-4E-BP / 4E-BP	Control	1.00 ± 0.00 (SD)			F(4, 15) = 5.664, p = 0.0055	
									Tanc2 shRNA	2.39 ± 0.72 (SD)		p = 0.0056, **		
									Deptor shRNA	1.11 ± 0.20 (SD)		p > 0.9999, ns		
									Tanc2 & Deptor shRNA	2.05 ± 0.61 (SD)		p = 0.0398, *		
									Tanc1 shRNA	1.47 ± 0.29 (SD)		p = 0.8059, ns		
								p-mTOR / mTOR	Control	1.00 ± 0.00 (SD)			F(4, 15) = 3.121, p = 0.0469	
									Tanc2 shRNA	1.25 ± 0.20 (SD)		p = 0.4619, ns		
									Deptor shRNA	1.07 ± 0.24 (SD)		p > 0.9999, ns		
									Tanc2 & Deptor shRNA	1.49 ± 0.13 (SD)		p = 0.0228, *		
									Tanc1 shRNA	0.19 ± 0.34 (SD)		p = 0.9187, ns		
								p-Akt (T308) / Akt	Control	1.00 ± 0.00 (SD)			F(4, 15) = 1.976, p = 0.1500	
									Tanc2 shRNA	0.93 ± 0.14 (SD)		p > 0.9999, ns		
									Deptor shRNA	1.23 ± 0.17 (SD)		p = 0.8083, ns		
									Tanc2 & Deptor shRNA	1.16 ± 0.20 (SD)		p > 0.9999, ns		
									Tanc1 shRNA	0.80 ± 0.46 (SD)		p > 0.9999, ns		
								p-Akt (S473) / Akt	Control	1.00 ± 0.00 (SD)			F(4, 15) = 8.58, p = 0.0008	
									Tanc2 shRNA	1.68 ± 0.28 (SD)		p = 0.0192, *		
									Deptor shRNA	1.17 ± 0.15 (SD)		p > 0.9999, ns		
									Tanc2 & Deptor shRNA	1.92 ± 0.51 (SD)		p = 0.0018, **		
									Tanc1 shRNA	0.97 ± 0.24 (SD)		p > 0.9999, ns		
								pGSK3β / GSK3β	Control	1.00 ± 0.00 (SD)			F(4, 15) = 14.53, p < 0.0001	
									Tanc2 shRNA	1.55 ± 0.19 (SD)		p = 0.0002, ***		
									Deptor shRNA	0.93 ± 0.08 (SD)		p > 0.9999, ns		
									Tanc2 & Deptor shRNA	1.41 ± 0.12 (SD)		p = 0.0034, **		
									Tanc1 shRNA	1.11 ± 0.19 (SD)		p > 0.9999, ns		
	d	Western blotting			DIV 28	n=4	Ratio (Fold change)	Tanc2 / actin	Control	1.00 ± 0.00 (SD)	One-way ANOVA Bonferroni's multiple comparison test		F(4, 15) = 34.27, p < 0.0001	
									Tanc2 shRNA	0.37 ± 0.15 (SD)		p < 0.0001, ***		
									Deptor shRNA	0.89 ± 0.02 (SD)		p = 0.8578, ns		
									Tanc2 & Deptor shRNA	0.32 ± 0.15 (SD)		p < 0.0001, ***		
									Tanc1 shRNA	1.00 ± 0.14 (SD)		p > 0.9999, ns		
								Deptor / actin	Control	1.00 ± 0.00 (SD)			F(4, 15) = 16.94, p < 0.0001	
									Tanc2 shRNA	0.99 ± 0.05 (SD)		p > 0.9999, ns		
									Deptor shRNA	0.50 ± 0.05 (SD)		p = 0.004, **		
									Tanc2 & Deptor shRNA	0.62 ± 0.16 (SD)		p = 0.0306, *		
									Tanc1 shRNA	1.40 ± 0.34 (SD)		p = 0.0199, *		
								Tanc1 / actin	Control	1.00 ± 0.00 (SD)			F(4, 15) = 25.82, p < 0.0001	
									Tanc2 shRNA	0.88 ± 0.15 (SD)		p = 0.6206, ns		
									Deptor shRNA	0.94 ± 0.12 (SD)		p > 0.9999, ns		
									Tanc2 & Deptor shRNA	0.78 ± 0.08 (SD)		p = 0.0562, ns		
									Tanc1 shRNA	0.31 ± 0.10 (SD)		p < 0.0001, ***		
								p-S6 / S6	Control	1.00 ± 0.00 (SD)			F(4, 15) = 19.21 p < 0.0001	
									Tanc2 shRNA	1.19 ± 0.13 (SD)		p > 0.9999, ns		
									Deptor shRNA	1.85 ± 0.35 (SD)		p = 0.007, **		
									Tanc2 & Deptor shRNA	1.74 ± 0.21 (SD)		p = 0.0196, *		
									Tanc1 shRNA	2.78 ± 0.55 (SD)		p < 0.0001, ***		
								p-4E-BP / 4E-BP	Control	1.00 ± 0.00 (SD)			F(4, 15) = 4.407, p = 0.0149	
									Tanc2 shRNA	1.42 ± 0.30 (SD)		p = 0.1126, ns		
									Deptor shRNA	1.65 ± 0.15 (SD)		p = 0.0073, **		
									Tanc2 & Deptor shRNA	1.55 ± 0.10 (SD)		p = 0.024, *		
									Tanc1 shRNA	1.54 ± 0.41 (SD)		p = 0.0257, *		
								p-mTOR / mTOR	Control	1.00 ± 0.00 (SD)			F(4, 15) = 4.077, p = 0.0197	
									Tanc2 shRNA	0.82 ± 0.07 (SD)		p = 0.1394, ns		
									Deptor shRNA	0.76 ± 0.18 (SD)		p = 0.0362, *		
									Tanc2 & Deptor shRNA	0.73 ± 0.06 (SD)		p = 0.0148, *		
									Tanc1 shRNA	0.73 ± 0.12 (SD)		p = 0.0164, *		
								p-Akt (T308) / Akt	Control	1.00 ± 0.00 (SD)			F(4, 15) = 4.659, p = 0.0121	
									Tanc2 shRNA	1.02 ± 0.16 (SD)		p > 0.9999, ns		
									Deptor shRNA	1.28 ± 0.15 (SD)		p = 0.0898, ns		
									Tanc2 & Deptor shRNA	1.29 ± 0.22 (SD)		p = 0.0627, ns		
									Tanc1 shRNA	0.94 ± 0.14 (SD)		p > 0.9999, ns		
								p-Akt (S473) / Akt	Control	1.00 ± 0.00 (SD)			F(4, 15) = 39.84, p < 0.0001	
									Tanc2 shRNA	1.06 ± 0.11 (SD)		p > 0.9999, ns		
									Deptor shRNA	1.53 ± 0.03 (SD)		p < 0.0001, ***		
									Tanc2 & Deptor shRNA	1.73 ± 0.04 (SD)		p < 0.0001, ***		
									Tanc1 shRNA	1.27 ± 0.17 (SD)		p = 0.0052, **		
								pGSK3β / GSK3β	Control	1.00 ± 0.00 (SD)			F(4, 15) = 15.56, p < 0.0001	
									Tanc2 shRNA	1.19 ± 0.01 (SD)		p = 0.1376, ns		
									Deptor shRNA	1.35 ± 0.13 (SD)		p = 0.0036, **		
									Tanc2 & Deptor shRNA	1.37 ± 0.15 (SD)		p = 0.002, **		
									Tanc1 shRNA	1.64 ± 0.17 (SD)		p < 0.0001, ***		
8	c	Western blotting			Day 14	n=4	Ratio (Fold change)	Tanc2 / actin	Control	1.00 ± 0.00 (SD)	One-way ANOVA Bonferroni's multiple comparison test		F(2, 9) = 87.04, p < 0.0001	
									Tanc2 shRNA1	0.27 ± 0.05 (SD)		p < 0.0001, ***		
									Tanc2 shRNA2	0.27 ± 0.14 (SD)		p < 0.0001, ***		
								p-S6 / S6	Control	1.00 ± 0.00 (SD)			F(2, 9) = 5.59 p = 0.0263	
									Tanc2 shRNA1	1.51 ± 0.17 (SD)		p = 0.0584, ns		
									Tanc2 shRNA2	1.62 ± 0.45 (SD)		p = 0.0243, *		
								p-4E-BP / 4E-BP	Control	1.00 ± 0.00 (SD)			F(2, 9) = 7.34, p = 0.0129	
									Tanc2 shRNA1	1.65 ± 0.40 (SD)		p = 0.0288, *		
									Tanc2 shRNA2	1.77 ± 0.34 (SD)		p = 0.0124, *		
								p-mTOR / mTOR	Control	1.00 ± 0.00 (SD)			F(2, 9) = 2.62, p = 0.1266	
									Tanc2 shRNA1	1.47 ± 0.39 (SD)		p = 0.3150, ns		
									Tanc2 shRNA2	1.68 ± 0.64 (SD)		p = 0.104, ns		
								p-Akt (S473) / Akt	Control	1.00 ± 0.00 (SD)			F(2, 9) = 11.41, p = 0.0034	
									Tanc2 shRNA1	0.77 ± 0.29 (SD)		p = 0.2209, ns		
									Tanc2 shRNA2	1.37 ± 0.09 (SD)		p = 0.0321, *		
								pGSK3β / GSK3β	Control	1.00 ± 0.00 (SD)			F(2, 9) = 5.83, p = 0.0237	
									Tanc2 shRNA1	1.97 ± 0.64 (SD)		p = 0.03, *		
									Tanc2 shRNA2	1.94 ± 0.46 (SD)		p = 0.0343, *		
	a	Novel object test		Male	2-3 months	WT = 17 HT = 17	Preference index (%)			WT = 61.12 ± 2.23	Student's t-test	p = 0.0529, ns	t(32) = 2.0100	Two-tailed
										HT = 67.60 ± 2.07				
							Distance moved (cm)			WT = 2221.67 ± 69.83	Mann-Whitney U	p = 0.0005, ***	U = 47	Two-tailed
										HT = 2789.45 ± 122.54				
							Speed (cm/s)			WT = 3.70 ± 0.11	Mann-Whitney U	p = 0.0006, ***	U = 48	Two-tailed
										HT = 4.64 ± 0.20				
							Exploration time (s)	Familiar object vs novel object	WT	F = 12.11 ± 1.26	Student's t-test	p = 0.0073, **	t(32) = 2.867	Two-tailed
										N = 18.31 ± 1.60				
									HT	F = 13.08 ± 1.66	Student's t-test	p = 0.0002, ***	t(32) = 4.258	Two-tailed
										N = 27.26 ± 0.15				
							Frequency of object exploration (count)	Familiar object vs novel object	WT	F = 24.17 ± 1.83	Mann-Whitney U	p = 0.9120, ns	U = 141	Two-tailed
										N = 26.00 ± 2.44				
									HT	F = 26.35 ± 4.00	Mann-Whitney U	p = 0.0114, *	U = 72	Two-tailed
										N = 41.47 ± 4.64				
									10 minutes	WT = 58.95 ± 2.63		p =0.0436, *		
										HT = 67.53 ± 2.95				
									20 minutes	WT = 40.13 ± 1.61		p = 0.0212, *		
										HT = 49.48 ± 2.15				

**Supplementary Fig. 1**	**b**	Open field test		Male	7-8 weeks	WT = 13<br>HT = 11	Distance moved (m)	Light off (0 lux)	30 minutes	WT = 36.29 ± 2.22<br>HT = 46.33 ± 1.58	Two-way ANOVA repeated measures Bonferroni's multiple comparison test	p = 0.0107, *	Interaction: F(5, 110) = 0.4458, p = 0.8155<br>Tanc2: F(1, 22) = 12.78, p = 0.0017<br>Time: F(5, 110) = 88.29, p < 0.0001	
									40 minutes	WT = 35.25 ± 2.10<br>HT = 40.99 ± 1.79		p = 0.4236, ns		
									50 minutes	WT = 29.28 ± 1.37<br>HT = 38.34 ± 2.24		p = 0.0280, *		
									60 minutes	WT = 34.54 ± 2.69<br>HT = 39.16 ± 2.71		p = 0.1020, ns		
							Total distance moved (m)		60 minutes	WT = 231.47 ± 10.06<br>HT = 281.86 ± 96.59	Student's t-test	p = 0.0017, **	t(22)= 3.575	Two-tailed
							Time spent in center region (s)		60 minutes	WT = 651.6 ± 76.00<br>HT = 733.7 ± 53.17	Mann-Whitney U	p = 0.1339, ns	U = 45	Two-tailed
	**c**	Laboras		Male	2-3 months	WT = 16<br>HT = 16	Distance moved (m)	Time (hr)	2	WT = 38.54 ± 2.75<br>HT = 49.688 ± 4.14	Two-way ANOVA repeated measures Bonferroni's multiple comparison test	p > 0.0010, **	Interaction: F(34, 510) = 3.07, p < 0.0001<br>Tanc2: F(1, 15) = 16.05, p = 0.0011<br>Time: F(34, 510) = 34.39, p < 0.0001	
									4	WT = 23.04 ± 2.53<br>HT = 37.49 ± 3.59		p > 0.0001, ***		
									6	WT = 21.79 ± 2.01<br>HT = 33.29 ± 3.86		p = 0.0005, ***		
									8	WT = 15.69 ± 2.47<br>HT = 28.11 ± 4.00		p = 0.0001, ***		
									10	WT = 18.37 ± 2.34<br>HT = 28.46 ± 3.75		p = 0.0051, **		
									12	WT = 13.34 ± 2.41<br>HT = 17.88 ± 3.58		p > 0.9999, ns		
									14	WT = 7.98 ± 1.58<br>HT = 9.06 ± 1.41		p > 0.9999, ns		
									16	WT = 3.33 ± 0.67<br>HT = 5.53 ± 1.01		p > 0.9999, ns		
									18	WT = 3.87 ± 0.69<br>HT = 5.39 ± 0.85		p > 0.9999, ns		
									20	WT = 4.36 ± 0.60<br>HT = 4.69 ± 0.42		p > 0.9999, ns		
									22	WT = 4.25 ± 0.52<br>HT = 5.62 ± 0.91		p > 0.9999, ns		
									24	WT = 16.65 ± 3.15<br>HT = 19.47 ± 0.41		p > 0.9999, ns		
									26	WT = 24.66 ± 2.65<br>HT = 29.82 ± 3.88		p > 0.9999, ns		
									28	WT = 23.99 ± 2.40<br>HT = 38.10 ± 3.32		p < 0.0001, ***		
									30	WT = 19.25 ± 2.36<br>HT = 31.87 ± 4.94		p < 0.0001, ***		
									32	WT = 17.84 ± 2.64<br>HT = 25.29 ± 4.34		p = 0.1710, ns		
									34	WT = 14.18 ± 1.69<br>HT = 20.56 ± 2.28		p = 0.5561, ns		
									36	WT = 13.18 ± 2.70<br>HT = 15.56 ± 2.39		p > 0.9999, ns		
									38	WT = 2.51 ± 0.48<br>HT = 6.70 ± 0.92		p > 0.9999, ns		
									40	WT = 3.19 ± 0.71<br>HT = 3.46 ± 0.50		p > 0.9999, ns		
									42	WT = 4.01 ± 0.50<br>HT = 3.86 ± 0.57		p > 0.9999, ns		
									44	WT = 3.26 ± 0.44<br>HT = 4.94 ± 0.74		p > 0.9999, ns		
									46	WT = 4.30 ± 0.46<br>HT = 6.65 ± 1.26		p > 0.9999, ns		
									48	WT = 15.71 ± 2.49<br>HT = 20.73 ± 4.00		p > 0.9999, ns		
									50	WT = 27.52 ± 1.92<br>HT = 35.50 ± 3.51		p = 0.0901, ns		
									52	WT = 24.14 ± 2.48<br>HT = 34.14 ± 4.60		p = 0.0058, **		
									54	WT = 17.46 ± 2.58<br>HT = 27.12 ± 5.41		p = 0.0094, **		
									56	1WT = 6.38 ± 2.46<br>HT = 23.13 ± 3.65		p = 0.3753, ns		
									58	WT = 15.36 ± 1.97<br>HT = 22.96 ± 2.98		p = 0.1438, ns		
									60	WT = 9.58 ± 1.62<br>HT = 12.71 ± 2.77		p > 0.9999, ns		
									62	WT = 3.27 ± 0.69<br>HT = 4.85 ± 0.74		p > 0.9999, ns		
									64	WT = 3.74 ± 0.70<br>HT = 4.13 ± 0.75		p > 0.9999, ns		
									66	WT = 3.82 ± 0.58<br>HT = 3.25 ± 0.57		p > 0.9999, ns		
									68	WT = 4.41 ± 0.47<br>HT = 5.16 ± 0.70		p > 0.9999, ns		
									70	WT = 6.37 ± 0.78<br>HT = 6.25 ± 1.36		p > 0.9999, ns		
	**d**	Elevated pluse maze		Male	2-3 months		Time spent in arm (s)		Open arm	WT = 104.98 ± 10.97<br>HT = 140.98 ± 18.30	Student's t-test	p = 0.0946, ns	t(22) = 1.747	Two-tailed
									Closed arm	WT = 340.70 ± 16.97<br>HT = 279.32 ± 21.05		p = 0.0316, *	t(22) = 2.296	Two-tailed
							Distance moved (m)			WT = 22.00 ± 1.44<br>HT = 24.91 ± 1.18	Student's t-test	p = 0.1417, ns	t(22) = 1.524	Two-tailed
	**e**	Light-dark box test		Male	2-3 months	WT = 13<br>HT = 10	Light zone (s)			WT = 191.19 ± 17.38<br>HT = 192.98 ± 14.30	Student's t-test	p = 0.9836, ns	t(20) = 0.0208	Two-tailed
							Frequency of tranfition (count)			WT = 66.16 ± 4.47<br>HT = 66.30 ± 4.49	Student's t-test	p = 0.9391, ns	t(20) = 0.0773	Two-tailed
**Supplementary Fig. 2**	**a**	3-chamber test		Male	2-3 months	WT = 14<br>HT = 14	Sniffing time (s)	Stranger vs Object	WT	O = 63.60 ± 6.12<br>S = 181.26 ± 10.84	Student's t-test	p < 0.0001, ***	t(26) = 9.45	Two-tailed
									HT	O = 59.46 ± 5.40<br>S = 158.85 ± 14.25	Student's t-test	p < 0.0001, ***	t(26) = 6.539	Two-tailed
							Chamber time (s)		WT	O = 178.33 ± 10.44<br>S = 314.38 ± 10.55	Student's t-test	p < 0.0001, ***	t(26) = 9.161	Two-tailed
									HT	O = 182.72 ± 15.16<br>S = 302.61 ± 17.93	Student's t-test	p < 0.0001, ***	t(26) = 5.104	Two-tailed
							Sniffing time (s)	Stranger 1 vs Stranger 2	WT	S1 = 75.50 ± 6.60<br>S2 = 120.31 ± 10.90	Student's t-test	p = 0.0016, **	t(26) = 3.515	Two-tailed
									HT	S1 = 64.15 ± 7.39<br>S2 = 120.65 ± 7.09	Student's t-test	p < 0.0001, ***	t(26) = 5.512	Two-tailed
							Chamber time (s)		WT	S1 = 217.72 ± 10.62<br>S2 = 257.03 ± 12.87	Student's t-test	p = 0.0263, *	t(26) = 2.355	Two-tailed
									HT	S1 = 193.32 ± 12.91<br>S2 = 271.35 ± 11.56	Student's t-test	p < 0.0001, ***	t(26) = 4.501	Two-tailed
	**b**	Pup ultrasonic vocalization		Pup	P3	WT = 23<br>HT = 23	Call duration (s)			WT = 5.93 ± 0.87<br>HT = 3.58 ± 0.58	Student's t-test	p = 0.0274, *	t(44) = 2.281	Two-tailed
							Call number (count)			WT = 163.39 ± 19.67<br>HT = 117.34 ± 12.5	Student's t-test	p = 0.0501, ns	t(44) = 2.051	Two-tailed
					P5	WT = 23<br>HT = 23	Call duration (s)			WT = 9.55 ± 1.38<br>HT = 5.70 ± 0.89	Mann-Whitney U	p = 0.0554, ns	U = 177	Two-tailed
							Call number (count)			WT = 225.11 ± 26.59<br>HT = 170.04 ± 18.19	Student's t-test	p = 0.0873, ns	t(44) = 1.749	Two-tailed
					P7	WT = 23<br>HT = 23	Call duration (s)			WT = 13.83 ± 1.25<br>HT = 9.02 ± 1.05	Student's t-test	p = 0.0047, **	t(44) = 2.982	Two-tailed
							Call number (count)			WT = 396.52 ± 20.88<br>HT = 261.65 ± 24.31	Student's t-test	p = 0.0017, **	t(42) = 3.358	Two-tailed
					P9	WT = 23<br>HT = 23	Call duration (s)			WT = 9.32 ± 1.59<br>HT = 6.03 ± 0.97	Mann-Whitney U	p = 0.1628, ns	U = 200.5	Two-tailed
							Call number (count)			WT = 266.26 ± 30.23<br>HT = 207.21 ± 23.65	Student's t-test	p = 0.1229, ns	t(44) = 1.573	Two-tailed
	**c**	Forced swimming test		Male	2-3 months	WT = 13<br>HT = 11	Immobility time (s)			WT = 171.07 ± 5.75<br>HT = 149.00 ± 8.89	Student's t-test	p = 0.0431, *	t(22) = 2.147	Two-tailed
	**d**	Tail suspension test		Male	2-3 months		Immobility time (s)			WT = 158.38 ± 6.15<br>HT = 150.27 ± 12.79	Student's t-test	p = 0.4543, ns	t(22) = 0.6005	Two-tailed
**Supplementary Fig. 3**	**a**	Open field test		Female	7-8 weeks	WT = 16<br>HT = 11	Distance moved (m)	Light off (0 lux)	10 minutes	WT = 62.34 ± 2.48<br>HT = 72.11 ± 2.79	Two-way ANOVA repeated measures Bonferroni's multiple comparison test	p =0.0274, *	Interaction: F(5, 125) = 0.8727, p = 0.5020<br>Tanc2: F(1, 25) = 7.9840, p = 0.0091<br>Time: F(5, 125) = 75.43, p < 0.0001	
									20 minutes	WT = 47.07 ± 1.55<br>HT = 53.60 ± 1.81		p = 0.3375, ns		
									30 minutes	WT = 42.46 ± 1.91<br>HT = 49.77 ± 3.11		p = 0.1976, ns		
									40 minutes	WT = 41.61 ± 1.96<br>HT = 46.34 ± 0.36		p = 0.9921, ns		
									50 minutes	WT = 38.50 ± 2.19<br>HT = 45.35 ± 3.08		p = 0.2701, ns		
									60 minutes	WT = 35.45 ± 2.33<br>HT = 45.82 ± 2.23		p = 0.0158, *		
							Speed (cm/s)	Light off (0 lux)	10 minutes	WT = 62.34 ± 2.48<br>HT = 72.11 ± 2.79	Two-way ANOVA repeated measures Bonferroni's multiple comparison test	p =0.0240, *	Interaction: F(5, 125) = 0.8693, p = 0.5039<br>Tanc2: F(1, 25) = 7.6160, p = 0.0107<br>Time: F(5, 125) = 76.91, p < 0.0001	
									20 minutes	WT = 47.07 ± 1.55<br>HT = 53.60 ± 1.81		p = 0.3084, ns		
									30 minutes	WT = 42.46 ± 1.91<br>HT = 49.77 ± 3.11		p = 0.2184, ns		
									40 minutes	WT = 41.61 ± 1.96<br>HT = 46.34 ± 0.36		p > 0.9999, ns		
									50 minutes	WT = 38.50 ± 2.19<br>HT = 45.35 ± 3.08		p = 0.3171, ns		
									60 minutes	WT = 35.45 ± 2.33<br>HT = 45.82 ± 2.23		p = 0.0247, *		
							Total distance moved (m)		60 minutes	WT = 267.46 ± 9.70<br>HT = 313.03 ± 1.34	Student's t-test	p = 0.0091, **	t(25)= 2.8260	Two-tailed
							Time spent in center region (s)		60 minutes	WT = 622.2 ± 61.7<br>HT = 539.4 ± 23.81	Mann-Whitney U	p = 0.4221, ns	U = 71	Two-tailed
	**b**	Elevated pluse maze				WT = 15<br>HT = 11	Time spent in arm (s)		Open arm	WT = 139.83 ± 10.90<br>HT = 201.73 ± 14.41	Two-way ANOVA Bonferroni's multiple comparison test	p = 0.0555, ns	Interaction: F(1, 48) = 18.56, p < 0.0001<br>Tanc2: F(1, 48) = 0.2230, p = 0.6389<br>Arm: F(1, 48) = 20.12, p < 0.0001	
									Closed arm	WT = 281.76 ± 19.77<br>[illegible]		p = 0.0087, **		

									[illegible]	HT = 204.60 ± 16.17		[illegible]		
	c	Light-dark box test			3-4 months	WT = 18<br>HT = 12	Light zone (s)			WT = 138.59 ± 11.30<br>HT = 139.09 ± 14.56	Student's t-test	p = 0.9790, ns	t(28) = 0.0266	Two-tailed
							Frequency of tranfition (count))			WT = 54.38 ± 3.35<br>HT = 55.83 ± 5.56	Student's t-test	p = 0.8151, ns	t(28) = 0.2361	Two-tailed
	d	Forced swimming test				WT = 14<br>HT = 10	Immobility time (s)			WT = 136.00 ± 11.97<br>HT = 107.1 ± 13.5	Student's t-test	p = 0.1276, ns	t(22) = 1.5840	Two-tailed
	e	Tail suspension test				WT = 13<br>HT = 10	Immobility time (s)			WT = 138.69 ± 11.13<br>HT = 139.00 ± 13.12	Student's t-test	p = 0.9858, ns	t(22) = 0.0180	Two-tailed
Supplementary Fig. 5	a	Coimmunoprecipitation (Western blotting)				n = 4	mTOR/Tanc1 ratio in HEK293T cells (Fold change)		Serum +	1.00 ± 0.00 (SD)	Student's t-test	p = 0.0258, *	t(6) = 2.945	Two-tailed
									-serum 4 hr	2.57 ± 0.53 (SD)				
	b					n = 3	mTOR/Tanc1 ratio in HEK293T cells (Fold change)	Vehicle	+serum	1.00 ± 0.00 (SD)	Student's t-test	p = 0.0467, *	t(4) = 2.843	Two-tailed
									-serum 4 hr	3.42 ± 0.85 (SD)				
								Rapamycin	+serum	1.38 ± 0.53 (SD)	Student's t-test	p = 0.9735, ns	t(4) = 0.0353	Two-tailed
									-serum 4 hr	1.36 ± 0.38 (SD)				
	c	Western blotting	Tanc1 overexpression (Empty vs Tanc1)					p-mTOR/mTOR		Empty = 1.00 ± 0.00 (SD)<br>Tanc1 = 0.72 ± 0.05 (SD)	Student's t-test	p = 0.0051, **	t(4) = 5.562	Two-tailed
			Tanc2 overexpression (Empty vs Tanc2)					p-mTOR/mTOR		Empty = 1.00 ± 0.00 (SD)<br>Tanc2 = 0.72 ± 0.04 (SD)	Student's t-test	p = 0.0041, **	t(4) = 5.907	Two-tailed
Supplementary Fig. 6	b	Western blotting (P2 fraction)				n=3	Protein levels normalized to α-tubulin (Fold change)	p-mTOR/mTOR	0 hr	1.00 ± 0.00 (SD)	One-way ANOVA Bonferroni's multiple comparison test		F(2, 6) = 36.97, p = 0.0004	
									0.5 hr	1.68 ± 0.25 (SD)		p = 0.0130, *		
									1 hr	2.02 ± 0.32 (SD)		p = 0.0003, ***		
								mTOR	0 hr	1.00 ± 0.00 (SD)	One-way ANOVA Bonferroni's multiple comparison test		F(2, 6) = 0.3685, p = 0.7064	
									0.5 hr	1.42 ± 0.45 (SD)		p = 0.8546, ns		
									1 hr	1.16 ± 0.40 (SD)		p > 0.9999, ns		
								Tanc1	0 hr	1.00 ± 0.00 (SD)	One-way ANOVA Bonferroni's multiple comparison test		F(2, 6) = 3.735, p = 0.0884	
									0.5 hr	1.04 ± 0.02 (SD)		p = 0.6933, ns		
									1 hr	1.02 ± 0.5 (SD)		p > 0.0820, ns		
								Tanc2	0 hr	1.00 ± 0.00 (SD)	One-way ANOVA Bonferroni's multiple comparison test		F(2, 6) = 9.172, p = 0.0150	
									0.5 hr	0.82 ± 0.02 (SD)		p = 0.0824, ns		
									1 hr	0.71 ± 0.07 (SD)		p = 0.0120, *		
								PSD-95	0 hr	1.00 ± 0.00 (SD)	One-way ANOVA Bonferroni's multiple comparison test		F(2, 6) = 7.057, p = 0.0265	
									0.5 hr	1.19 ± 0.05 (SD)		p = 0.0649, ns		
									1 hr	1.21 ± 0.04 (SD)		p = 0.0434, *		
								p-Raptor/Raptor	0 hr	1.00 ± 0.00 (SD)	One-way ANOVA Bonferroni's multiple comparison test		F(2, 6) = 12.54, p = 0.0072	
									0.5 hr	1.47 ± 0.10 (SD)		p = 0.0087, **		
									1 hr	1.12 ± 0.0.06 (SD)		p = 0.5771, ns		
								Raptor	0 hr	1.00 ± 0.00 (SD)	One-way ANOVA Bonferroni's multiple comparison test		F(2, 6) = 0.9666, p = 0.4326	
									0.5 hr	1.17 ± 0.11 (SD)		p > 0.9999, ns		
									1 hr	1.42 ± 0.17 (SD)		p = 0.6469, ns		
								PRAS40	0 hr	1.00 ± 0.00 (SD)	One-way ANOVA Bonferroni's multiple comparison test		F(2, 6) = 14.92, p = 0.0047	
									0.5 hr	1.30 ± 0.15 (SD)		p = 0.1272, ns		
									1 hr	1.71 ± 0.05 (SD)		p = 0.0038, **		
								Deptor	0 hr	1.00 ± 0.00 (SD)	One-way ANOVA Bonferroni's multiple comparison test		F(2, 6) = 5.93, p = 0.0379	
									0.5 hr	1.00 ± 0.13 (SD)		p > 0.9999, ns		
									1 hr	1.47 ± 0.14 (SD)		p = 0.0488, *		
Supplementary Fig. 7	c	Western blotting				n=3	Protein levels normalized to β-actin (Fold change)	Tanc1_total	E18	1.00 ± 0.00 (SD)				
									P1	0.68 ± 0.09 (SD)				
									P7	1.33 ± 0.43 (SD)				
									P14	6.22 ± 3.34 (SD)				
									P21	11.8 ± 4.05 (SD)				
									P28	15.62 ± 5.54 (SD)				
								Tanc2_total	E18	1.00 ± 0.00 (SD)				
									P1	0.81 ± 0.07 (SD)				
									P7	1.11 ± 0.32 (SD)				
									P14	1.07 ± 0.49 (SD)				
									P21	0.78 ± 0.28 (SD)				
									P28	0.62 ± 0.28 (SD)				
								PSD-95_total	E18	1.00 ± 0.00 (SD)				
									P1	1.1 ± 0.22 (SD)				
									P7	5.10 ± 1.16 (SD)				
									P14	15.2 ± 3.72 (SD)				
									P21	19.83 ± 8.45 (SD)				
									P28	23.25 ± 11.21 (SD)				
								Deptor_total	E18	1.00 ± 0.00 (SD)				
									P1	0.88 ± 0.11 (SD)				
									P7	1.59 ± 0.36 (SD)				
									P14	3.18 ± 1.28 (SD)				
									P21	4.90 ± 3.23 (SD)				
									P28	4.82 ± 3.39 (SD)				
								PRAS40_total	E18	1.00 ± 0.00 (SD)				
									P1	0.92 ± 0.08 (SD)				
									P7	1.25 ± 0.33 (SD)				
									P14	1.39 ± 0.45 (SD)				
									P21	1.78 ± 0.98 (SD)				
									P28	1.68 ± 1.01 (SD)				
								Tanc1_P2	P7	1.00 ± 0.00 (SD)				
									P14	3.60 ± 1.22 (SD)				
									P21	5.01 ± 2.50 (SD)				
									P28	6.75 ± 3.59 (SD)				
								Tanc2_P2	P7	1.00 ± 0.00 (SD)				
									P14	0.71 ± 0.15 (SD)				
									P21	0.46 ± 0.19 (SD)				
									P28	0.33 ± 0.13 (SD)				
								PSD-95_P2	P7	1.00 ± 0.00 (SD)				
									P14	3.38 ± 0.53 (SD)				
									P21	5.08 ± 2.17 (SD)				
									P28	6.51 ± 2.84 (SD)				
								Deptor_P2	P7	1.00 ± 0.00 (SD)				
									P14	2.75 ± 1.07 (SD)				
									P21	3.03 ± 1.00 (SD)				
									P28	3.48 ± 1.52 (SD)				
								PRAS40_P2	P7	1.00 ± 0.00 (SD)				
									P14	1.25 ± 0.33 (SD)				
									P21	1.09 ± 0.47 (SD)				
									P28	1.04 ± 0.47 (SD)				
Supplementary Fig. 8	a	Western blotting	Control vs Tanc2 shRNA		DIV14	n=3	Protein levels normalized to b-actin (Fold change)	Tanc2/actin	Control<br>Tanc2 shRNA	1.00 ± 0.00 (SD)<br>0.41 ± 0.09 (SD)	Student's t-test	p = 0.0004, ***	t(4) = 10.66	
								p-S6/S6	Control<br>Tanc2 shRNA	1.00 ± 0.00 (SD)<br>1.73 ± 0.17 (SD)	Student's t-test	p = 0.0019, **	t(4) = 7.27	
								p-4E-BP/4E-BP	Control<br>Tanc2 shRNA	1.00 ± 0.00 (SD)<br>1.57 ± 0.13 (SD)	Student's t-test	p = 0.0018, **	t(4) = 7.4	
								p-Akt/Akt	Control<br>Tanc2 shRNA	1.00 ± 0.00 (SD)<br>1.30 ± 0.09 (SD)	Student's t-test	p = 0.0045, **	t(4) = 5.761	
								p-GSK3β/GSK3β	Control<br>Tanc2 shRNA	1.00 ± 0.00 (SD)<br>1.39 ± 0.09 (SD)	Student's t-test	p = 0.0017, **	t(4) = 7.456	
	b							p-S6/S6	Control<br>Tanc2 shRNA	1.00 ± 0.00 (SD)<br>1.10 ± 0.05 (SD)	Student's t-test	p = 0.0273, *	t(4) = 3.397	
								p-4E-BP/4E-BP	Control<br>Tanc2 shRNA	1.00 ± 0.00 (SD)<br>1.01 ± 0.05 (SD)	Student's t-test	p = 0.6689, ns	t(4) = 0.4607	
								p-Akt/Akt	Control<br>Tanc2 shRNA	1.00 ± 0.00 (SD)<br>1.07 ± 0.08 (SD)	Student's t-test	p = 0.2364, ns	t(4) = 1.392	
								p-GSK3β/GSK3β	Control<br>Tanc2 shRNA	1.00 ± 0.00 (SD)<br>1.02 ± 0.13 (SD)	Student's t-test	p = 0.7421, ns	t(4) = 0.3528	
	c	Immunocytochemistry	NeuN vs GFAP			n=13	Marker / DAPI (%)	Neuron culture	NeuN<br>GFAP	86.98 ± 5.09 (SD)<br>1.03 ± 2.6 (SD)	Student's t-test	p < 0.0001, ***	t(24) = 54.13	
								Glia culture	NeuN<br>GFAP	4.01 ± 2.4 (SD)<br>80.77 ± 10.34 (SD)	Student's t-test	p < 0.0001, ***	t(24) = 26.06	

Supplementary table 2. Primers used in this study

Mouse line	Primer name	Sequence (5'-)
Tanc2 $^{+/-}$	Tanc2$^{+/-}$-F	GGTTAGATAGGAAATAACCATCA
	WT-B	ATGTGGCATATAAAGTTATACCTC
	Tanc2$^{+/-}$-B	TCCCCACAACGGGTTCTTCTG
Tanc2 $^{fl/fl}$	Tanc2-fl-F	CTGTGCATGAAAGCTTCTGG
	Tanc2-fl-B	TCTCCACCTCCCTGGTACTG
