# Supplementary material for: Tanc2-mediated mTOR inhibition balances mTORC1/2 signaling in the developing mouse brain and human neurons
Source: Nat Commun. 2021 May 11;12:2695. doi: 10.1038/s41467-021-22908-4 (PMC8113471; doi:10.1038/s41467-021-22908-4)
Supplement: Supplementary file 3 — Reporting Summary [file 41467_2021_22908_MOESM3_ESM.pdf]

nature research

Corresponding author(s): Eunjoon Kim

Last updated by author(s): Mar 31, 2021

Reporting Summary

Nature Research wishes to improve the reproducibility of the work that we publish. This form provides structure for consistency and transparency in reporting. For further information on Nature Research policies, see Authors & Referees and the Editorial Policy Checklist .

Statistics

For all statistical analyses, confirm that the following items are present in the figure legend, table legend, main text, or Methods section.

n/a	Confirmed	
☐	☒	The exact sample size (*n*) for each experimental group/condition, given as a discrete number and unit of measurement
☐	☒	A statement on whether measurements were taken from distinct samples or whether the same sample was measured repeatedly
☐	☒	The statistical test(s) used AND whether they are one- or two-sided<br>*Only common tests should be described solely by name; describe more complex techniques in the Methods section.*
☒	☐	A description of all covariates tested
☒	☐	A description of any assumptions or corrections, such as tests of normality and adjustment for multiple comparisons
☐	☒	A full description of the statistical parameters including central tendency (e.g. means) or other basic estimates (e.g. regression coefficient) AND variation (e.g. standard deviation) or associated estimates of uncertainty (e.g. confidence intervals)
☐	☒	For null hypothesis testing, the test statistic (e.g. *F*, *t*, *r*) with confidence intervals, effect sizes, degrees of freedom and *P* value noted<br>*Give P values as exact values whenever suitable.*
☒	☐	For Bayesian analysis, information on the choice of priors and Markov chain Monte Carlo settings
☒	☐	For hierarchical and complex designs, identification of the appropriate level for tests and full reporting of outcomes
☐	☒	Estimates of effect sizes (e.g. Cohen's *d*, Pearson's *r*), indicating how they were calculated

Our web collection on statistics for biologists contains articles on many of the points above.

Software and code

Policy information about availability of computer code

Data collection	LI-COR Odyssey (version) Fc<br>pClamp 10.1 (Molecular Devices)<br>Multiclamp Commander 700B (Molecular Devices)
Data analysis	Ethovision XT 10.1 (Noldus)<br>Clampfit 10 (Molecular Devices)<br>Image Studio Lite Ver. 5.2.5<br>GraphPad Prism 8.0<br>ImageJ (NIH)<br>Abobe Illustrator CS5

For manuscripts utilizing custom algorithms or software that are central to the research but not yet described in published literature, software must be made available to editors/reviewers. We strongly encourage code deposition in a community repository (e.g. GitHub). See the Nature Research guidelines for submitting code & software for further information.

Data

Policy information about availability of data

All manuscripts must include a data availability statement. This statement should provide the following information, where applicable:

- Accession codes, unique identifiers, or web links for publicly available datasets
- A list of figures that have associated raw data
- A description of any restrictions on data availability

The data supporting the findings of this study are available within the Supplementary Table 1. Other source data related to the study are available from the corresponding author upon reasonable request.

Field-specific reporting

Please select the one below that is the best fit for your research. If you are not sure, read the appropriate sections before making your selection.

☒ Life sciences ☐ Behavioural & social sciences ☐ Ecological, evolutionary & environmental sciences

For a reference copy of the document with all sections, see nature.com/documents/nr-reporting-summary-flat.pdf

Life sciences study design

All studies must disclose on these points even when the disclosure is negative.

Sample size	No statistical methods were used to predetermine sample size. Estimates were made based on our previous experience, experimental approach, availability and feasibility required to obtain statistically significant results. For detail sample size please see figure legends.
Data exclusions	Outliers were excluded based on the results of the ROUT test (Q = 1%).
Replication	All experiments were replicated through multiple cohort/mice analysis, where applicable. All replication attempts were successful. In the case of single experiments (multiple samples in a single experiment), such experimental designs were described in the legends
Randomization	Mice were allocated into specific cohorts at random, except genotype per cage post-weaning was set at a 1:1 ratio for WT vs HT. Male cohorts were caged separately from female cohorts if weaned. Pup and juvenile mice were not weaned and not separated, with experiments being performed with whole cages 'as is'.
Blinding	All experimenters were blind to the genotype of the mice (sex could not be occluded from the experimenter, due to obviousness of the features). All analyses were performed in a blind manner. Cohorts were grouped at random at time of weaning.

Reporting for specific materials, systems and methods

We require information from authors about some types of materials, experimental systems and methods used in many studies. Here, indicate whether each material, system or method listed is relevant to your study. If you are not sure if a list item applies to your research, read the appropriate section before selecting a response.

Materials & experimental systems

n/a	Involved in the study	
☐	☒	Antibodies
☐	☒	Eukaryotic cell lines
☒	☐	Palaeontology
☐	☒	Animals and other organisms
☒	☐	Human research participants
☒	☐	Clinical data

Methods

n/a	Involved in the study	
☒	☐	ChIP-seq
☒	☐	Flow cytometry
☒	☐	MRI-based neuroimaging

Antibodies

Antibodies used

Rb polyclonal anti-pan-Tanc (made in-house #1609, 1:1000)
Gp polyclonal anti-Tanc2 (made in-house #2183, 1:1000)
Rb polyclonal anti-Akt (Cell Signaling, Cat # 9272, lot # 28, 1:1000)
Rb polyclonal anti-p-Akt S473 (Cell Signaling, Cat # 9271, lot # 14, 1:1000)
Rb polyclonal anti-p-Akt S473 (Cell Signaling, Cat # 4060, lot # 25, 1:1000)
Rb monoclonal anti-p-Akt T308 (Cell Signaling, Cat #13038, lot # 5, clone # D25E6, 1:500)
Rb monoclonal anti-GSK3beta (Cell Signaling, Cat # 9315, lot # 14, clone # 27C10, 1:2000)
Rb polyclonal anti-pGSK3beta S9 (Cell Signaling, Cat # 9336, lot # 13, 1:2000)

Ms monoclonal anti-PKCalpha (BD, Cat # 610108, lot # 4261632, clone # 3/PKCa, 1:1000)
Rb polyclonal anti-pPKCalpha S657/T658 (EMD Millipore, Cat # 07-790, lot # 3284319, 1:1000)
Rb monoclonal anti-4E-BP (Cell Signaling, Cat # 9644, lot # 10, clone # 53H11, 1:1000)
Rb polyclonal anti-p-4E-BP T37/46 (Cell Signaling, Cat # 2855, lot # 26, 1:500)
Rb monoclonal anti-S6 (Cell Signaling, Cat # 2217, lot # 7, clone # 5G10, 1:1000)
Rb monoclonal anti-pS6 S235/236 (Cell Signaling, Cat # 4858, lot # 16, clone # D57.2.2E, 1:2000)
Ms monoclonal anti-mTOR (Invitrogen, Cat # AHO1232, lot # QD218356, clone # 215Q18, 1:1000)
Rb polyclonal anti-pmTOR S2448 (Cell Signaling, Cat # 2971, lot # unkown, 1:1000)
Rb monoclonal anti-S6K (Cell Signaling, Cat # 2708, lot # unknown, clone # 49D7, 1:1000)
Rb polyclonal anti-p-S6K T389 (Cell Signaling, Cat # 9205, lot # unknown, 1:1000)
Ms monoclonal anti-PSD-95 (NeuroMab, Cat # 75-028, lot # K28/43, 1:5000)
Rb monoclonal anti-PRAS40 (Cell Signaling, Cat # 2691, lot # 8, clone # D23C7, 1:1000)
Rb polyclonal anti-Deptor (Merck, Cat # ABS222, lot # unknown, 1:1000)
Rb polyclonal anti-Raptor (Merck, Cat # 09-217, lot # 3090003, 1:1000)
Rb polyclonal anti-p-Raptor S877 (Merck, Cat # 09-107, lot # unknown, 1:1000)
Rb polyclonal anti-Rictor (Thermo, Cat # A300-459A, lot # 4, 1:1000)
Ms monoclonal anti-Flag (MBL, Cat # M185-3L, lot # unknown, 1:1000)
Ms monoclonal anti-Myc (Cell Signaling, Cat # 2276, lot # 24, clone # 9B11, 1:1000)
Rb monoclonal anti-TSC1 (Cell Signaling, Cat # 6935, lot # 3, clone # D43E2, 1:1000)
Rb monoclonal anti-TSC2 (Cell Signaling, Cat # 4308, lot # 5, clone # D93F12, 1:1000)
Rb monoclonal anti-p-TSC2 T1462 (Cell Signaling, Cat # 3617, lot # , clone # 5B12, 1:1000)
Rb monoclonal anti-PTEN (Cell Signaling, Cat # 9559, lot # 17, clone # 138G6, 1:1000)
Rb monoclonal anti-p-PTEN S380/T382/383(Cell Signaling, Cat # 9549, lot # 5, clone # 44A7, 1:1000)
Rb monoclonal anti-p-PTEN T1462 (Cell Signaling, Cat # 3617, lot # 7, clone # 5B12, 1:1000)
Rb monoclonal anti-PI3K (Cell Signaling, Cat # 4249, lot # unknown, clone # C73F8, 1:1000)
Rb polyclonal anti-p-PI3K T458 (Cell Signaling, Cat # 4228, lot # 8, 1:1000)
Ms monoclonal anti-alpha-tubulin (DSHB, Cat # 12G100, lot # unknown, 1:1000)
Ms monoclonal anti-beta-actin (Sigma, Cat # A5316, lot # 048M4843V, clone # AC-74, 1:5000)
Gp polyclonal anti-NeuN (EMD Millipore, Cat # ABN90, lot # unknown, 1:1000)
Ms monoclonal anti-GFAP(Sigma, Cat # G3893, lot # unknown, 1:1000)
HRP conjugated anti-Rb IgG (Kackson, Cat # 711-035-152, 1:10000)
HRP conjugated anti-Ms IgG (Kackson, Cat # 715-035-150, 1:10000)
HRP conjugated anti-Gp IgG (Kackson, Cat # 706-035-148, 1:10000)
IRDye 800CW conjugated anti-Ms IgG (Li-Cor, Cat # 926-32212, 1:5000)
IRDye 800CW conjugated anti-Rb IgG (Li-Cor, Cat # 926-32213, 1:5000)
IRDye 680RD conjugated anti-Ms IgG (Li-Cor, Cat # 926-68072, 1:5000)
IRDye 680RD conjugated anti-Rb IgG (Li-Cor, Cat # 926-68073, 1:5000)
Alexa Fluor 488 AffiniPure $F(ab')_2$ Fragment Donkey Anti-MS IgG (H+L) (Jackson, Cat # 715-546-150, 1:1000)
Alexa Fluor 594 AffiniPure $F(ab')_2$ Fragment Donkey Anti-Gp IgG (H+L) (Jackson, Cat # 706-586-148, 1:1000)

Validation

Rb anti-Pan-Tanc and Gp anti-Tanc2 antibodies made in-house have been validated with the respective Tanc2 KO mice by immunoblotting.
All commercial antibodies have been validated and published, with relevant information existing in the pertaining website.

Eukaryotic cell lines

Policy information about cell lines

Cell line source(s)

293T (ATCC CRL-3216)
HuES6 (ATCC SCRC-1040)

Authentication

None as directly purchased from ATCC

Mycoplasma contamination

Cells were tested negative for mycoplasma.

Commonly misidentified lines
(See ICLAC register)

None of the cell-lines used are listed in the ICLAC database

Animals and other organisms

Policy information about studies involving animals; ARRIVE guidelines recommended for reporting animal research

Laboratory animals

C56BL/6J strain were used as background of all wildtype/mutant mouse used in the study. Only male mice were used for production of data. Behavioral studies involved adult mice (> postnatal day 70), while all other data involved juvenile (postnatal day 20~28) mice.

Wild animals

Study did not involve wild animals

Field-collected samples	Study did not involve samples collected from the field
Ethics oversight	All animals used in this study were maintained and procedures performed in accord with the Requirements of Animal Research at KAIST. Experimental procedures were approved by the Committee on Animal Research at KAIST (KA2012-19).

Note that full information on the approval of the study protocol must also be provided in the manuscript.
