# Supplementary material for: Tanc2-mediated mTOR inhibition balances mTORC1/2 signaling in the developing mouse brain and human neurons
Source: Nat Commun. 2021 May 11;12:2695. doi: 10.1038/s41467-021-22908-4 (PMC8113471; doi:10.1038/s41467-021-22908-4)

Fig. 2 a uncropped images

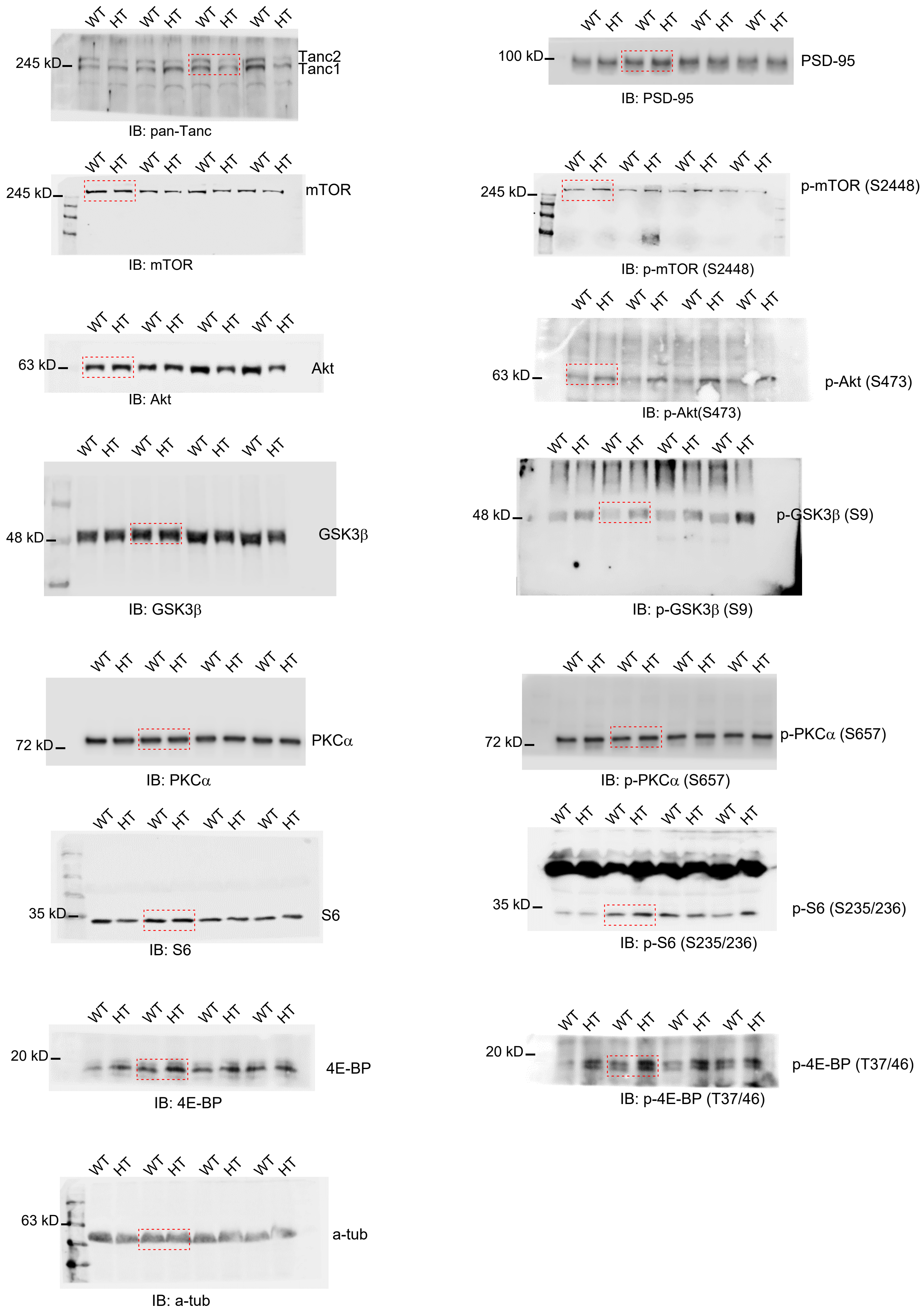

Fig. 2 b uncropped images

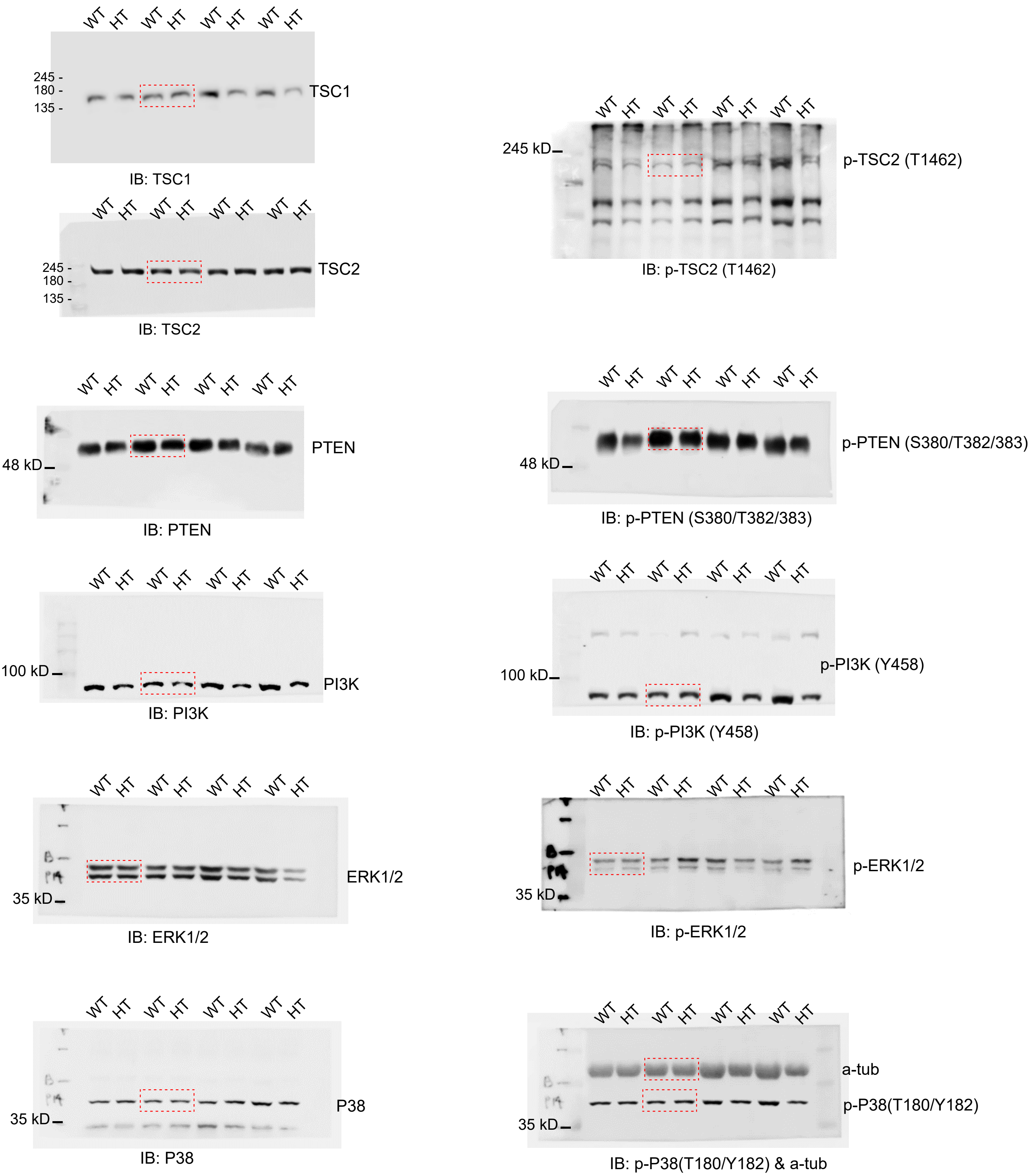

Fig. 2 c uncropped images

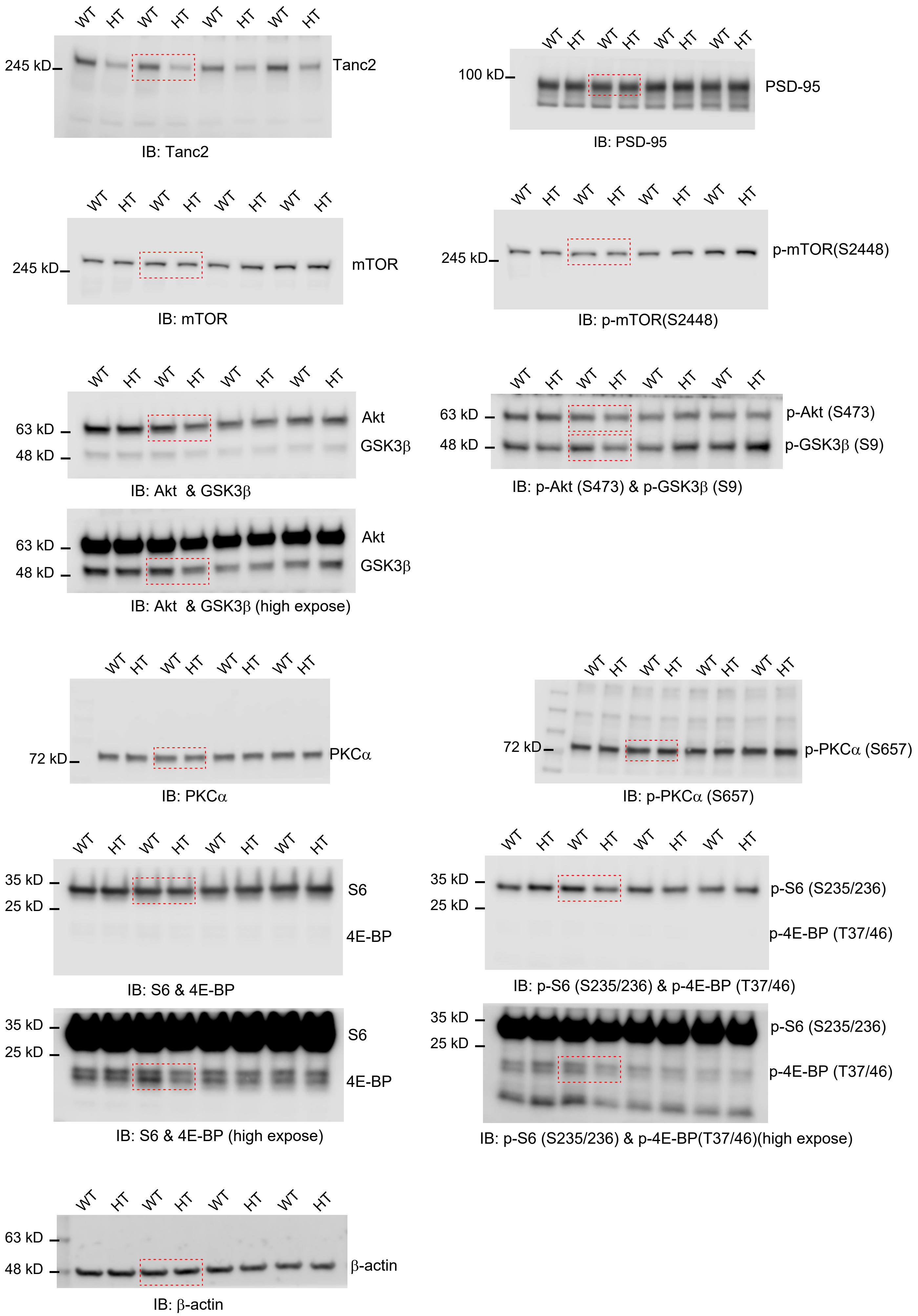

Fig. 2 d uncropped images

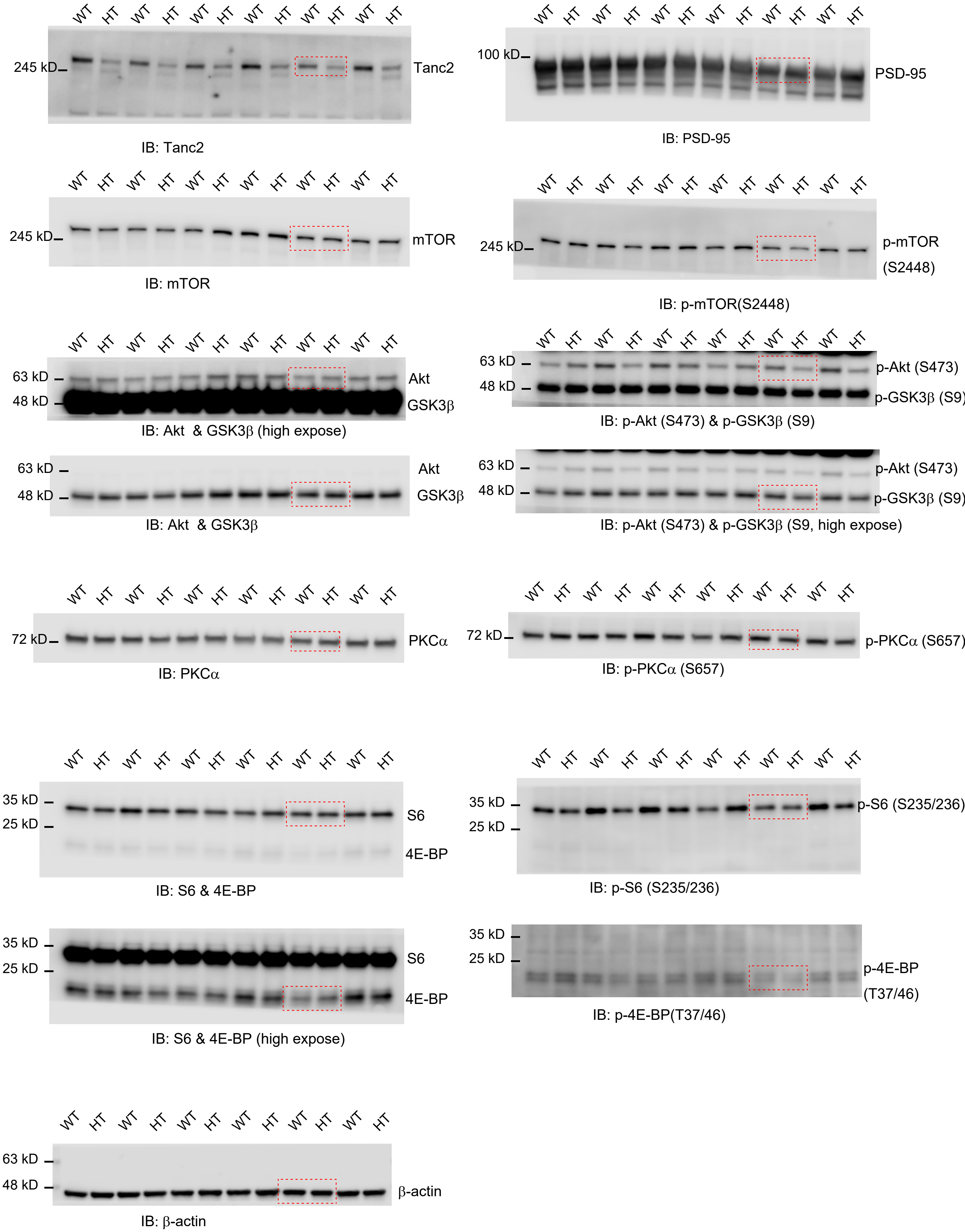

Fig. 2 h uncropped images

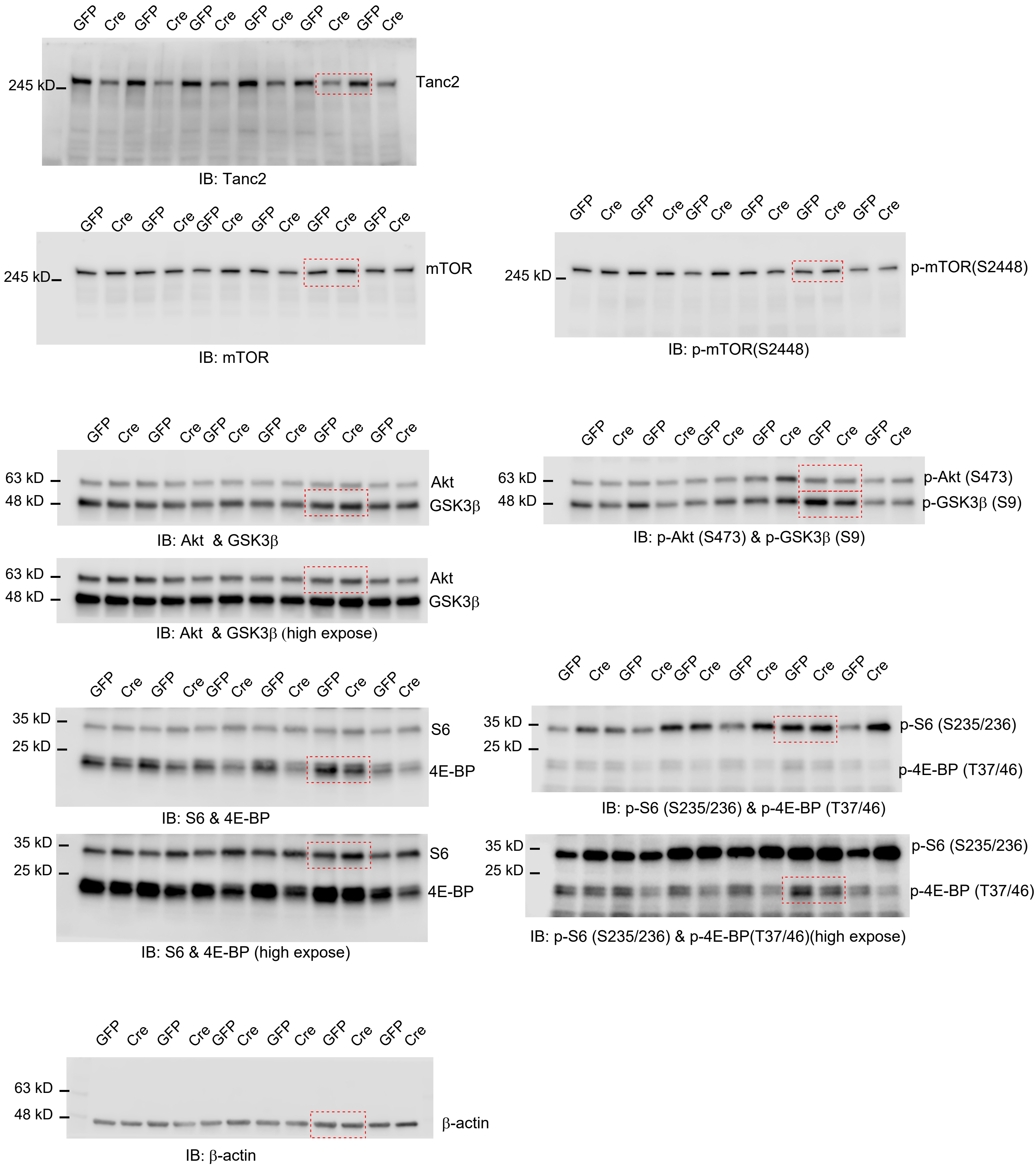

Fig. 2 f uncropped images

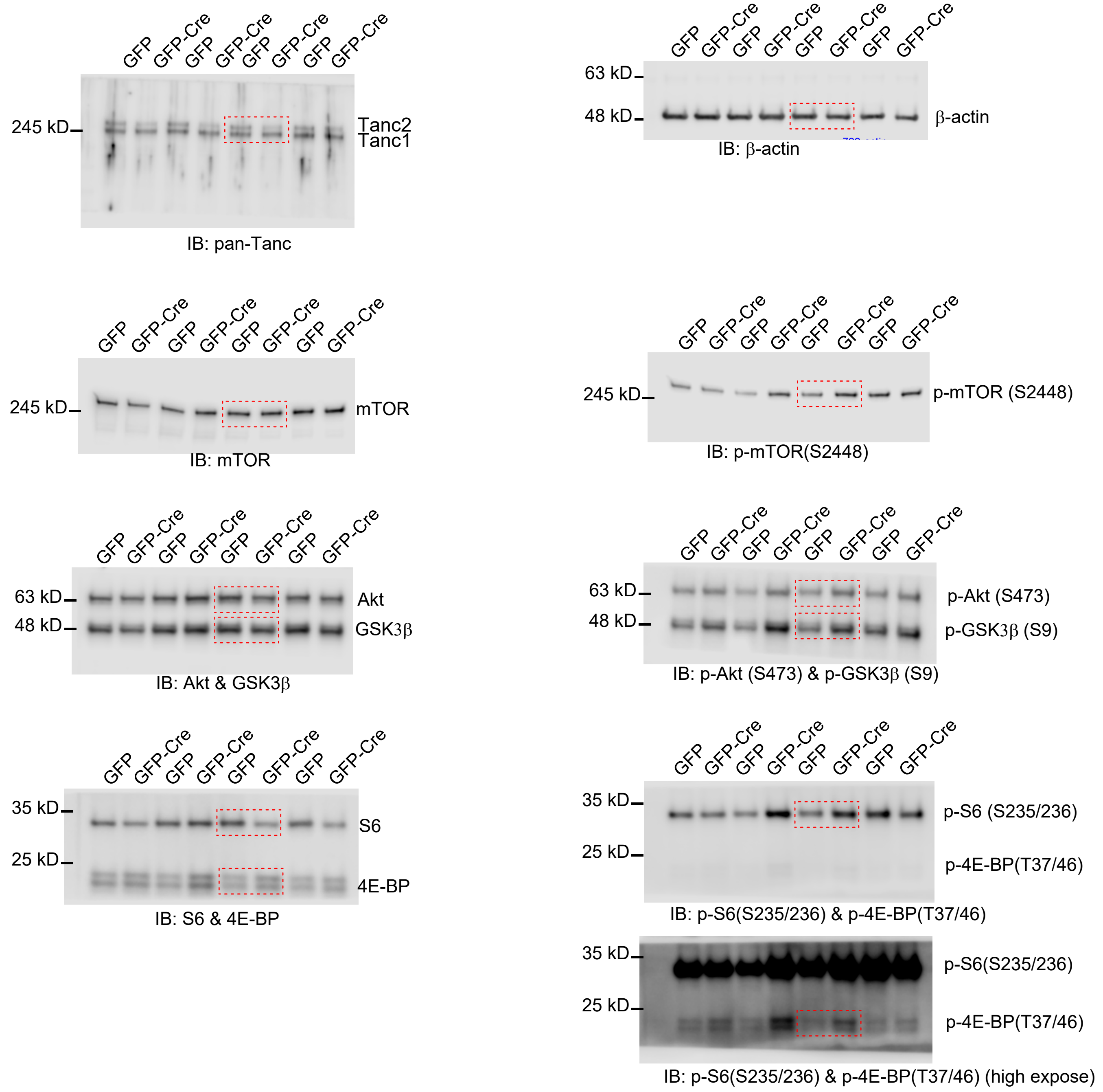

Fig. 4 a uncropped images

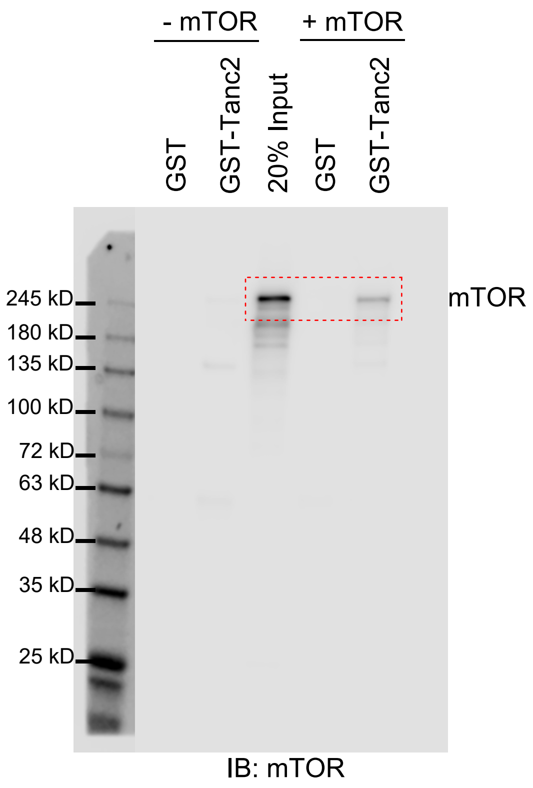

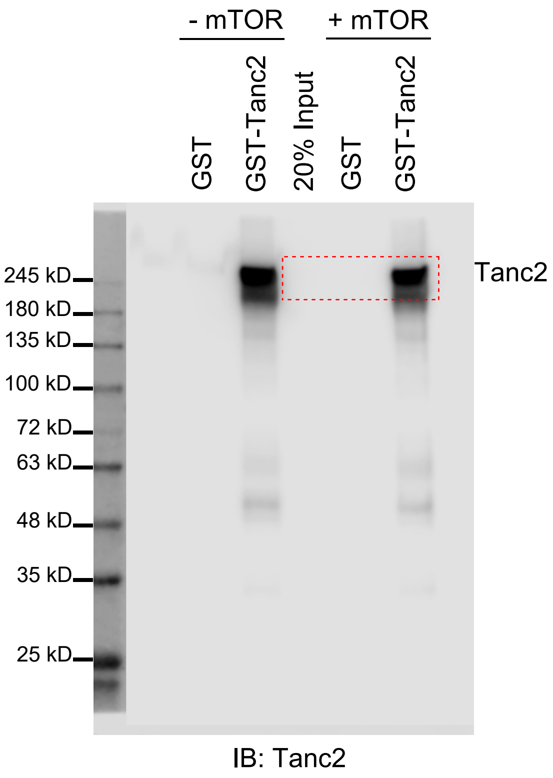

Fig. 4 b uncropped images

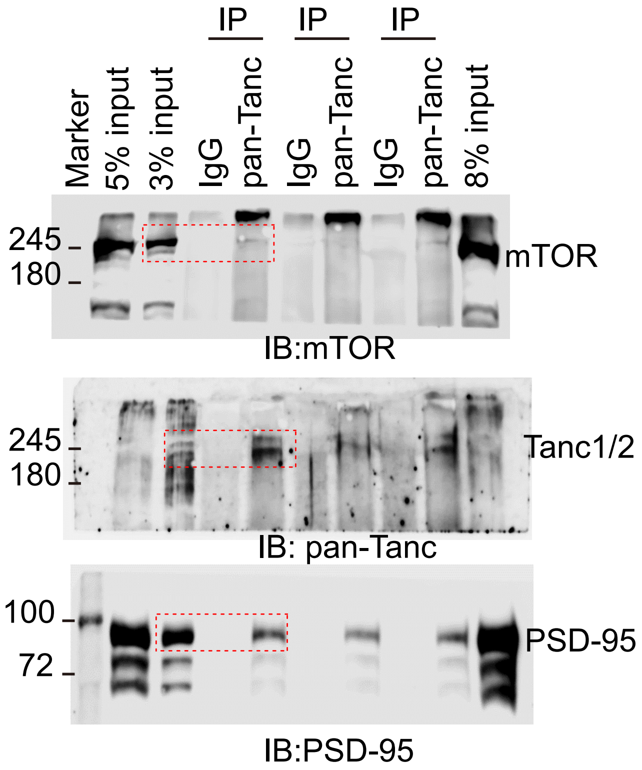

Fig. 4 c uncropped images

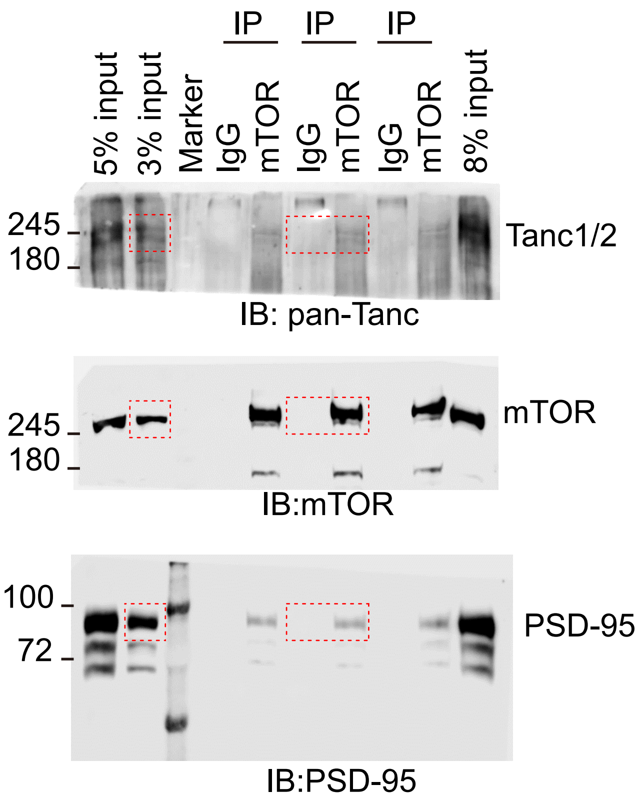

Fig. 4 d uncropped images

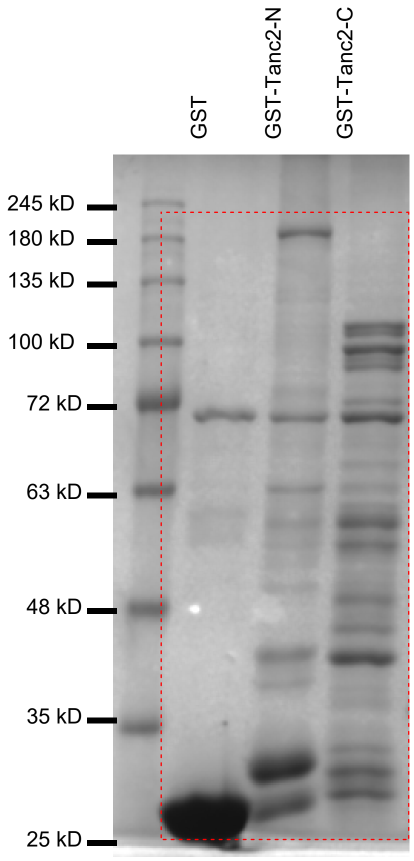

Coomassie Blue staining

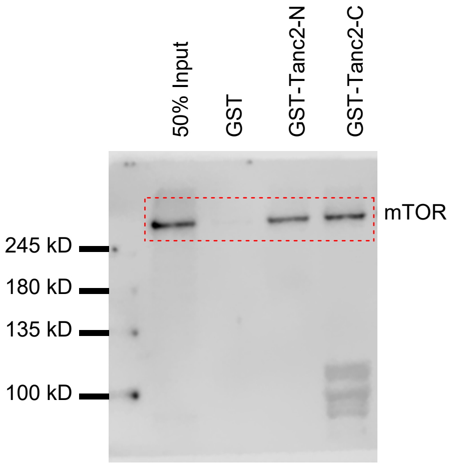

IB: mTOR

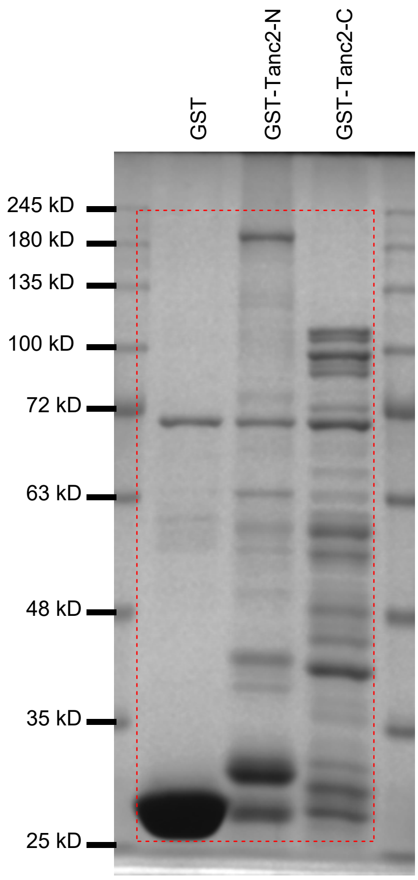

Coomassie Blue staining

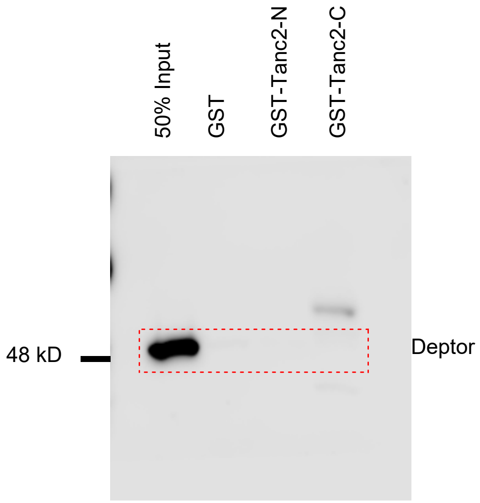

IB: Deptor

Fig. 4 e uncropped images

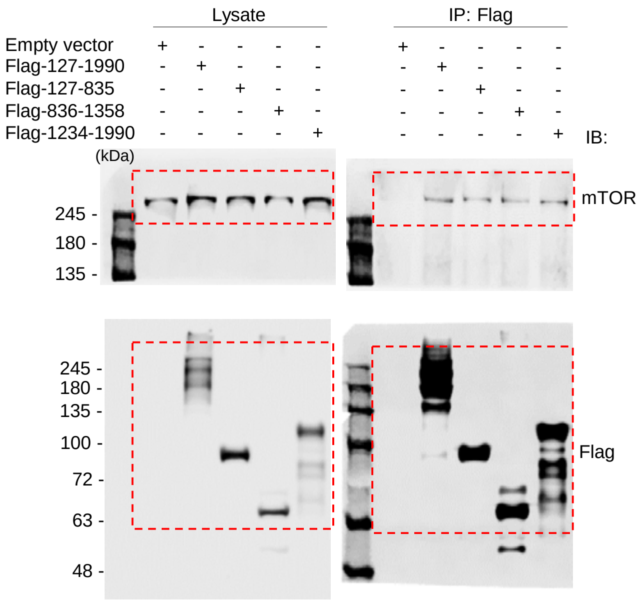

Fig. 4 f uncropped images

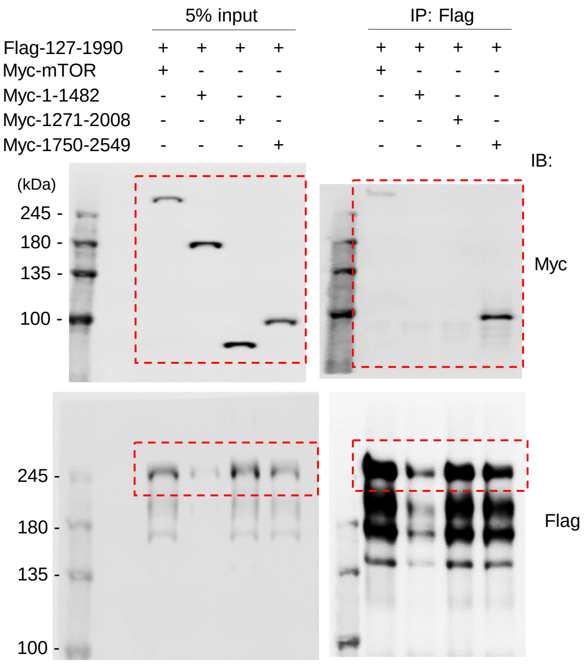

Fig. 5 a uncropped images

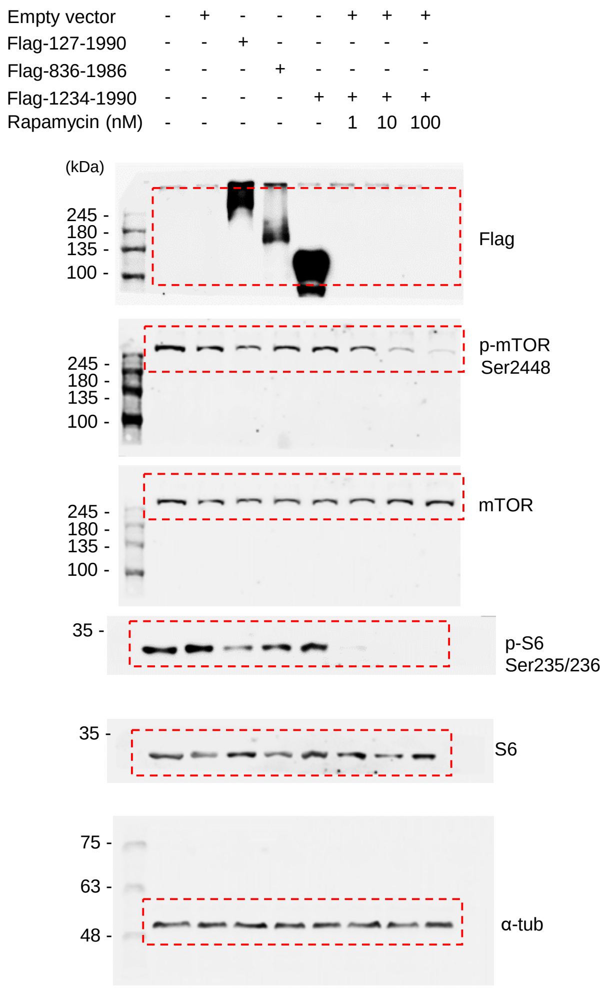

Fig. 5 b uncropped images

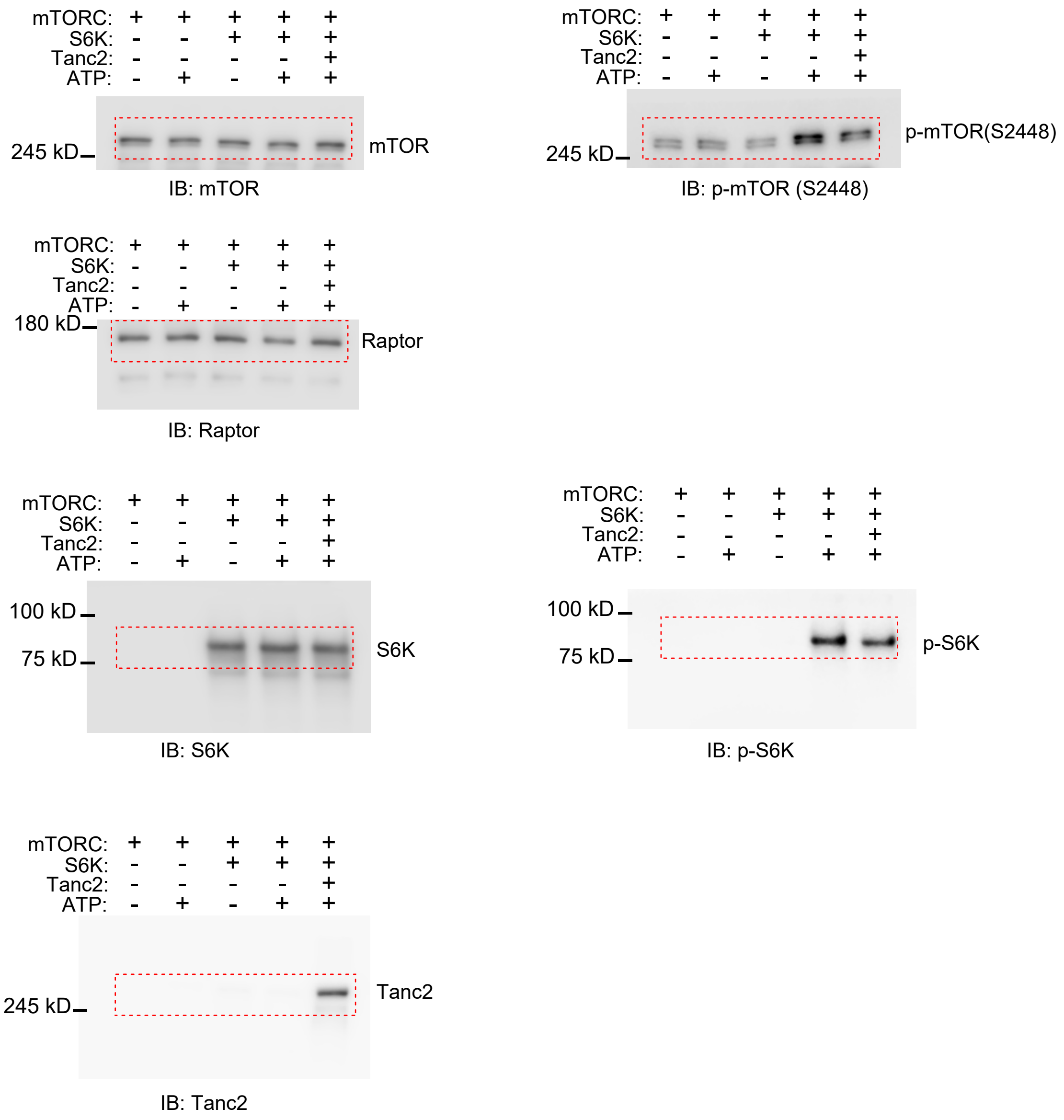

Fig. 5 c uncropped images

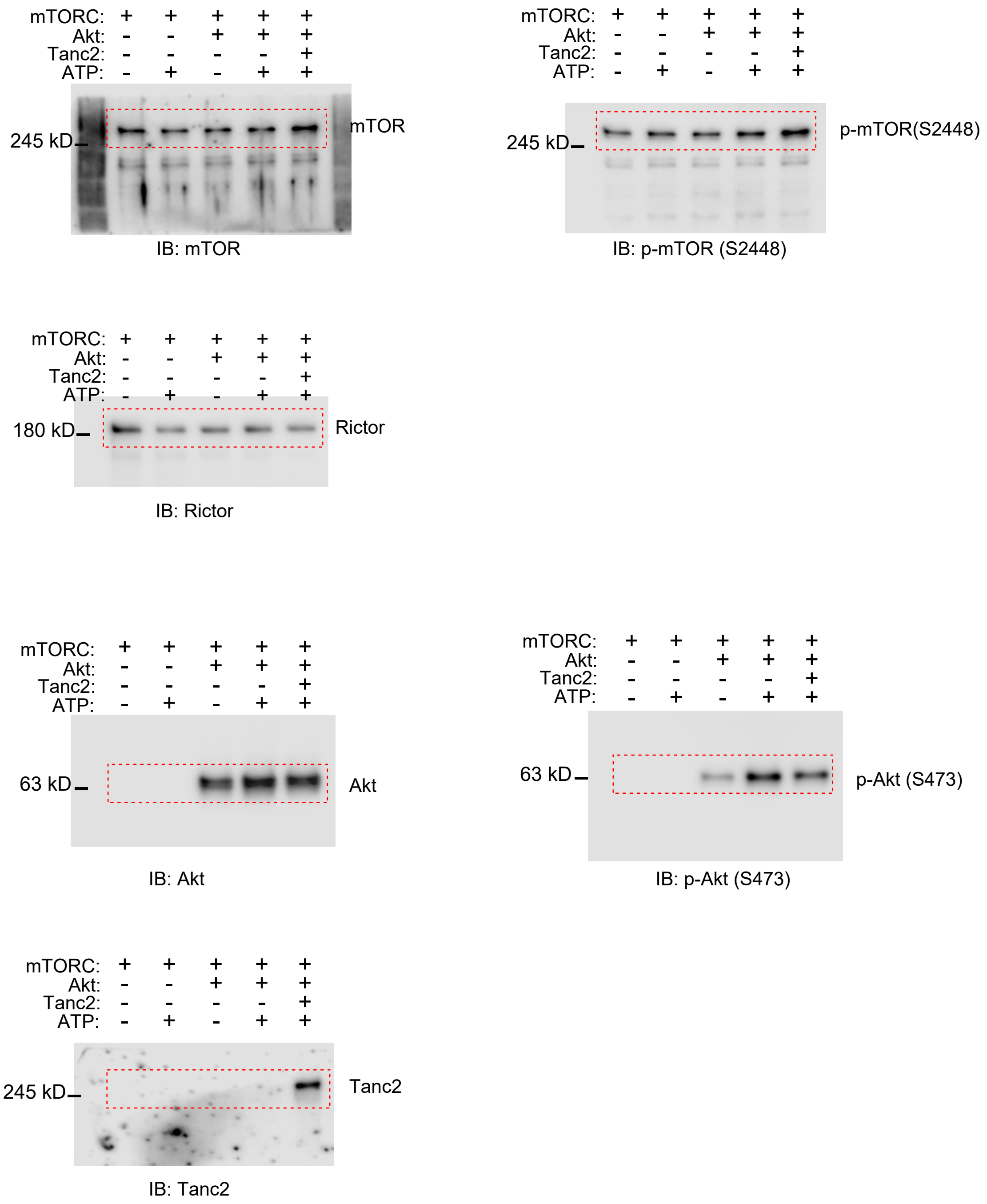

Fig. 6 c uncropped images

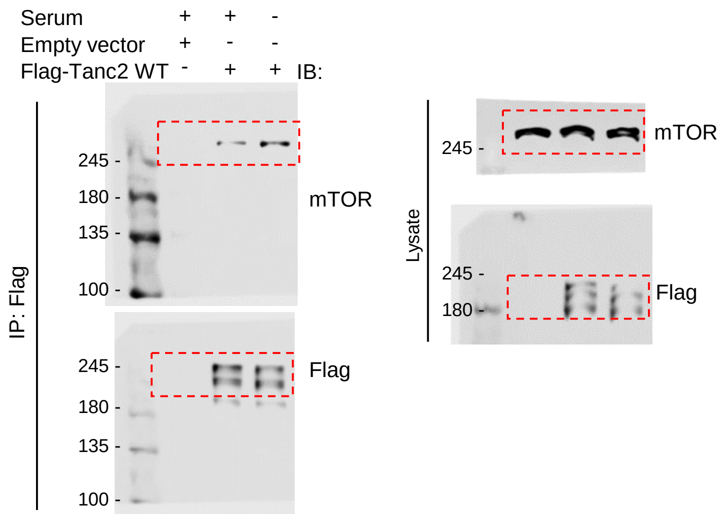

Fig. 6 d uncropped images

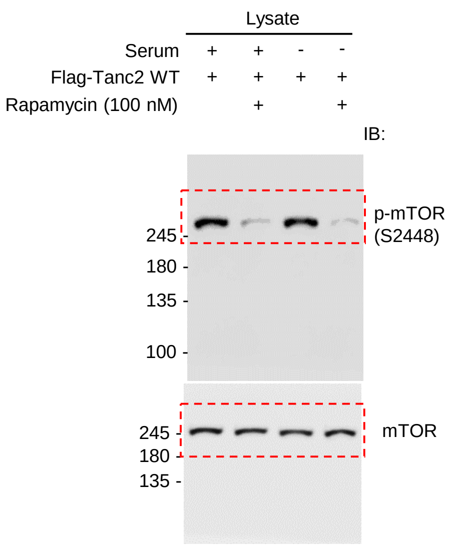

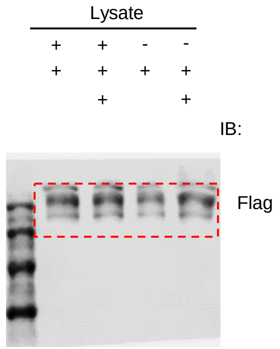

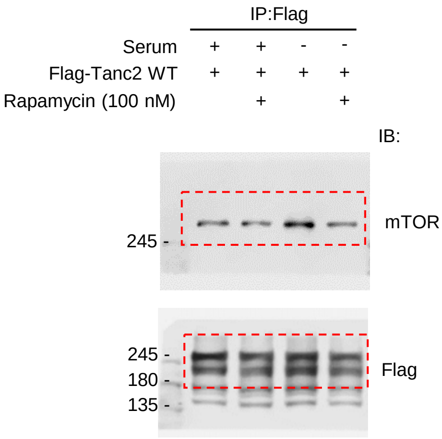

Fig. 6 e uncropped images

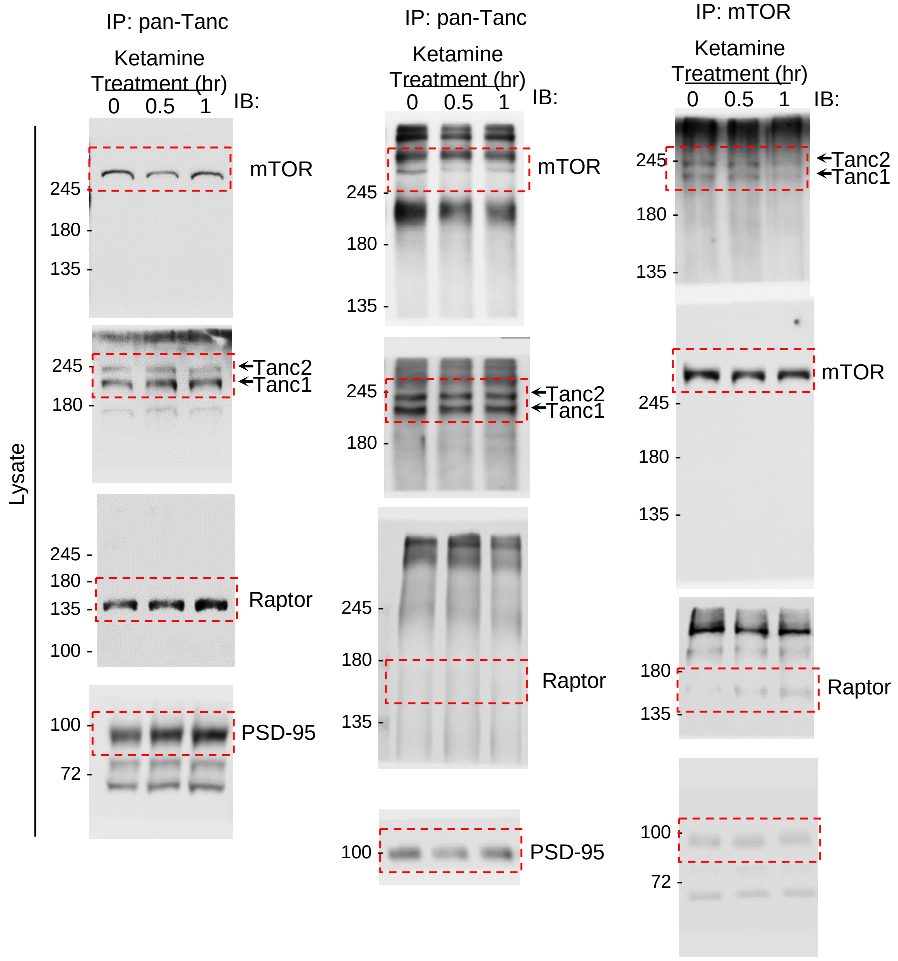

Fig. 7 b uncropped images

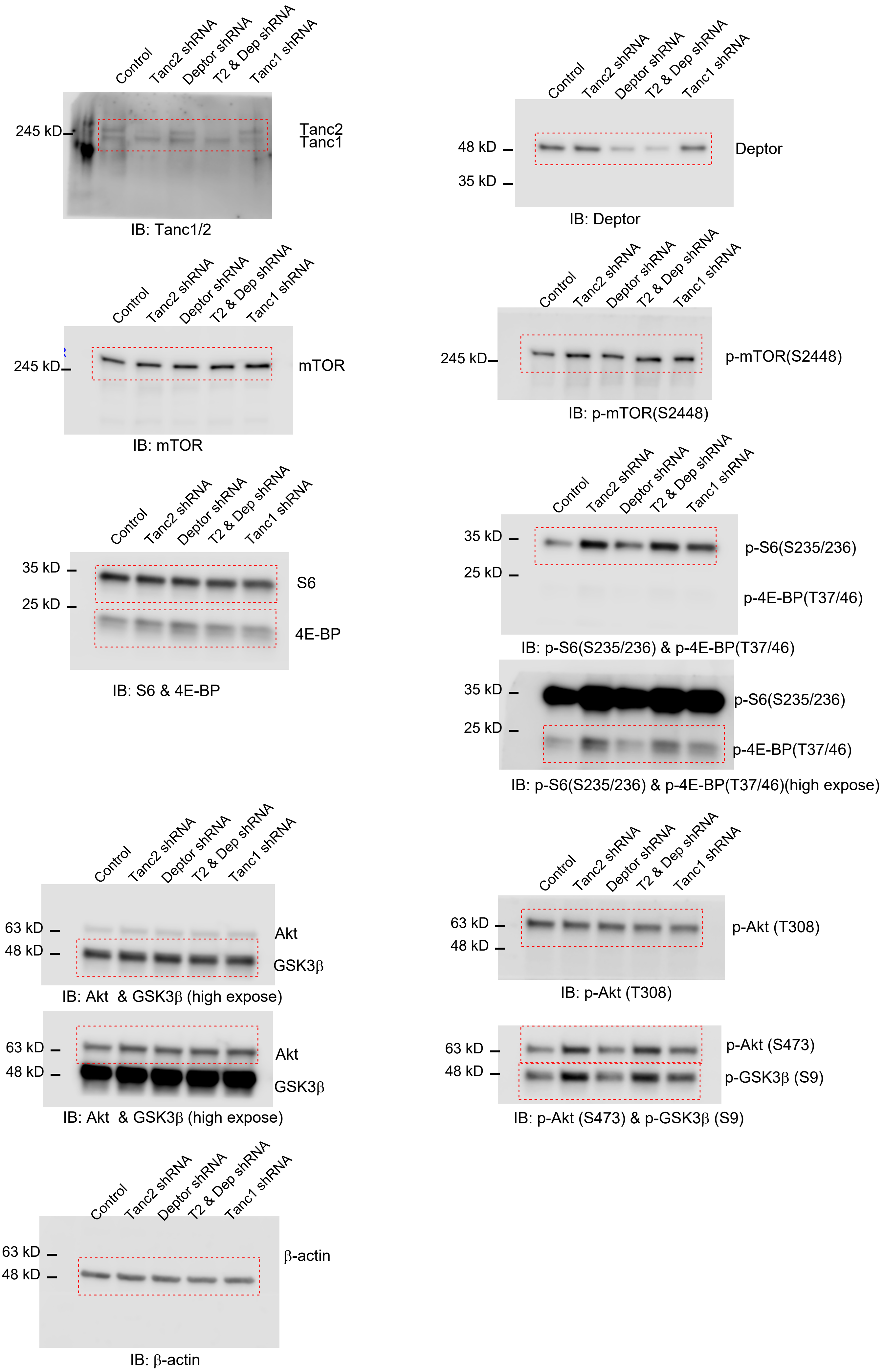

Fig. 7 d uncropped images

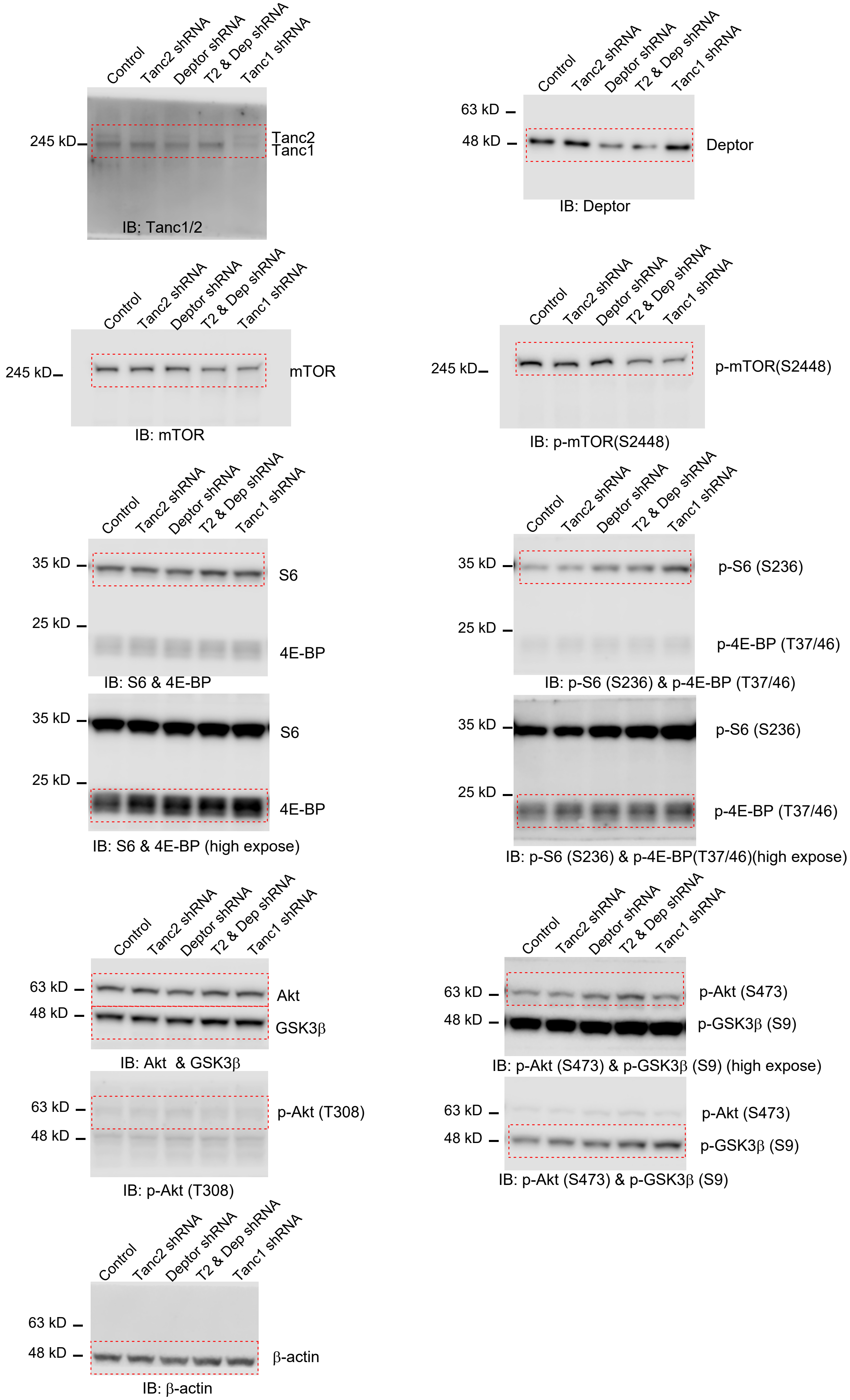

Fig. 8 b uncropped images

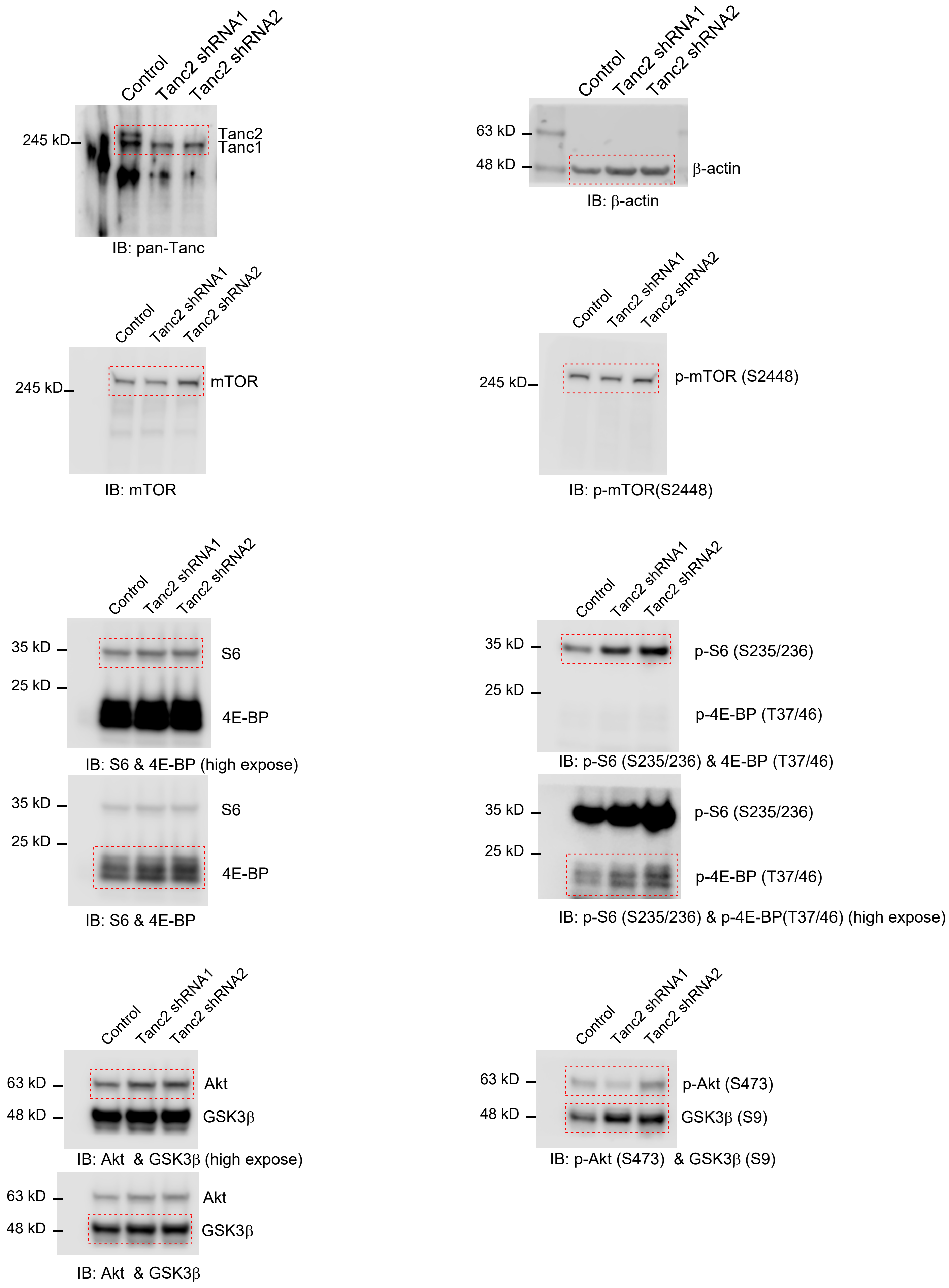

Fig. S5 a uncropped images

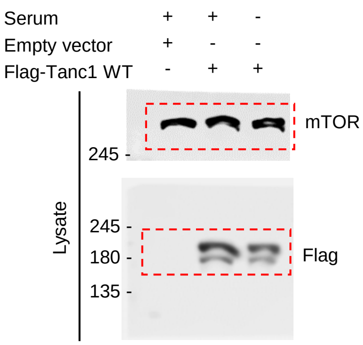

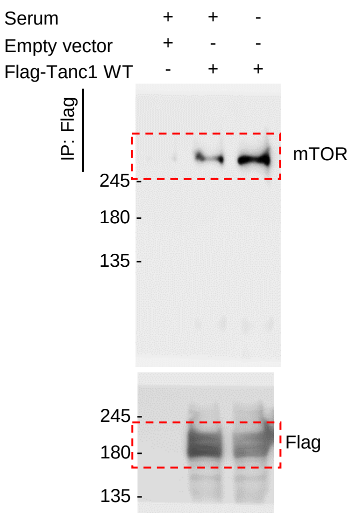

Fig. S5 b uncropped images

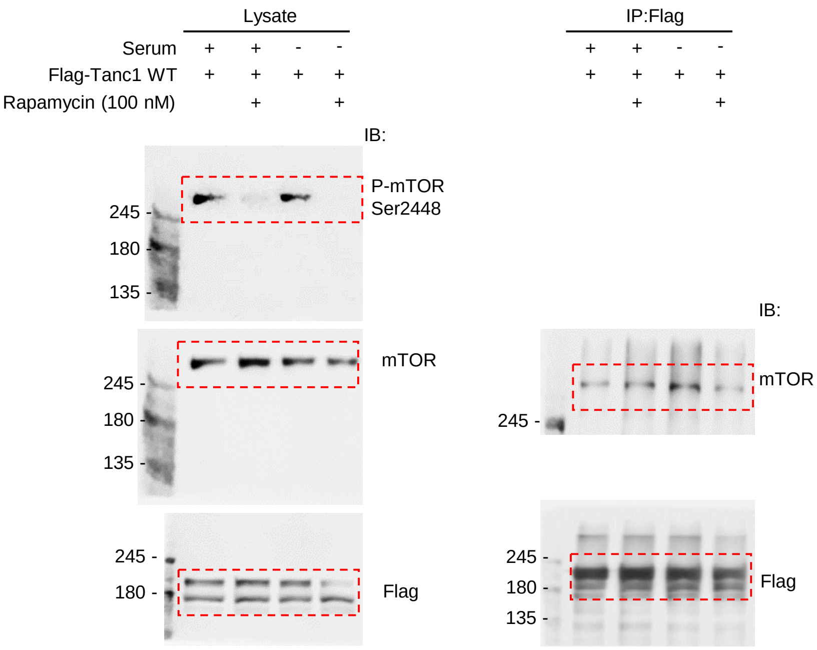

Fig. S5 c uncropped images

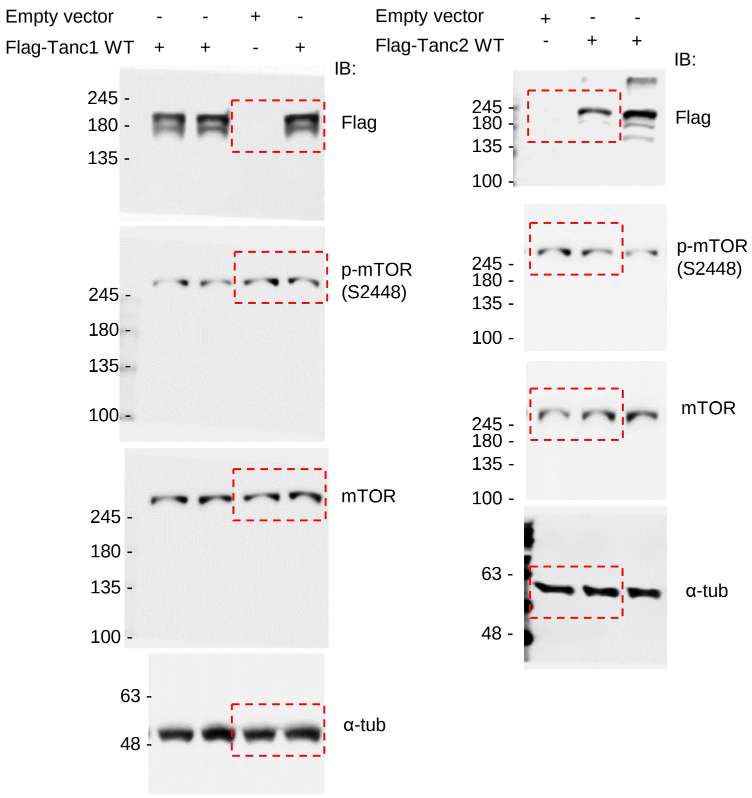

Fig. S6 a uncropped images

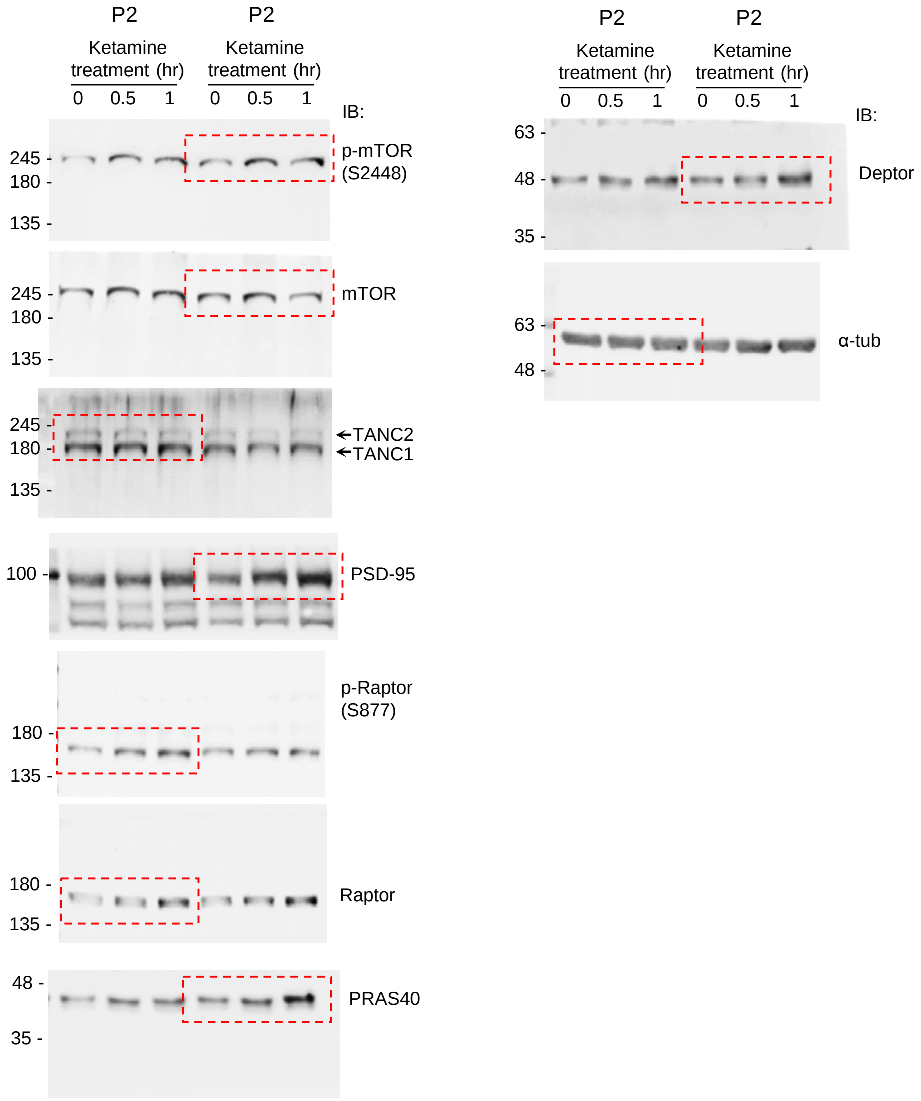

Fig. S7 a uncropped images

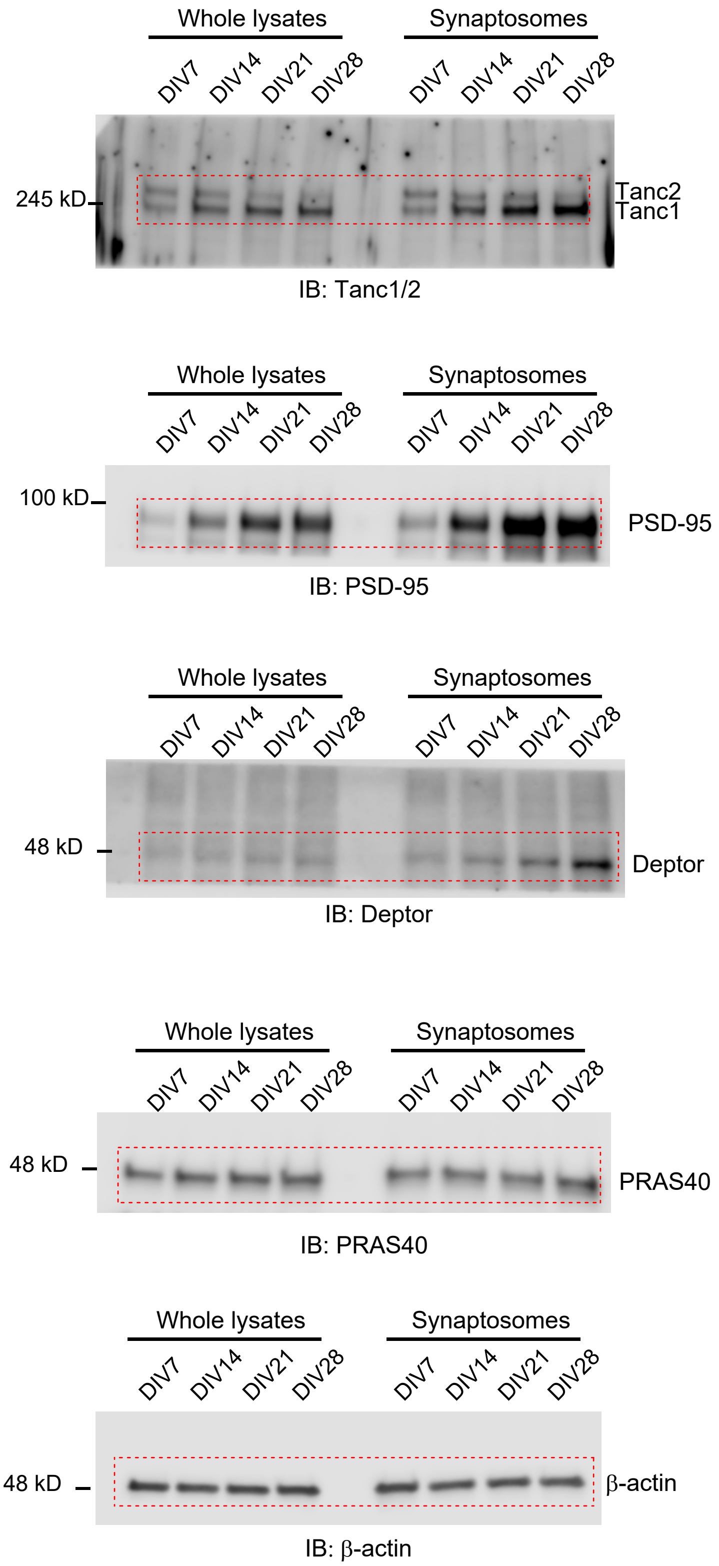

Fig. S7 b uncropped images

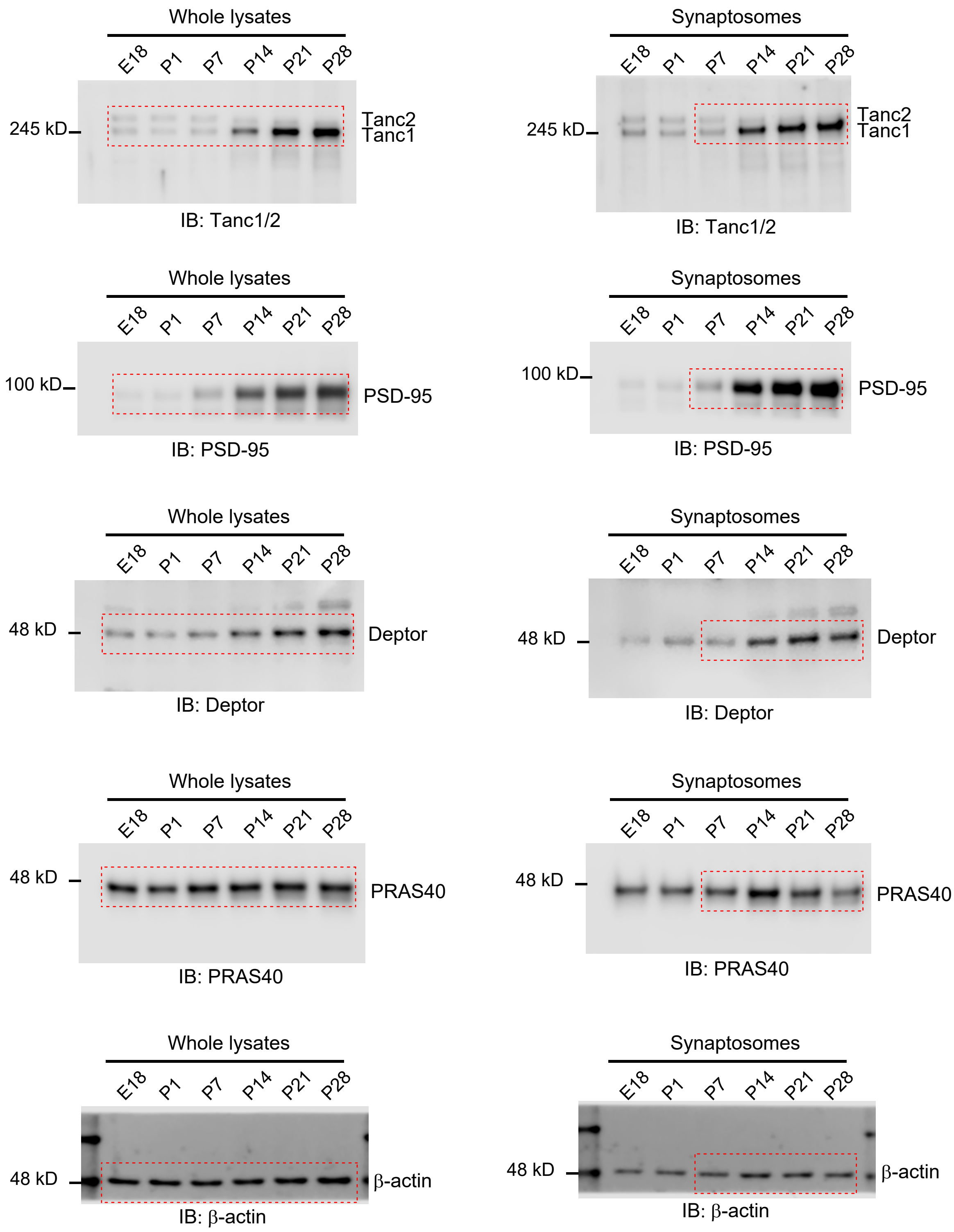

Fig. S8 a uncropped images

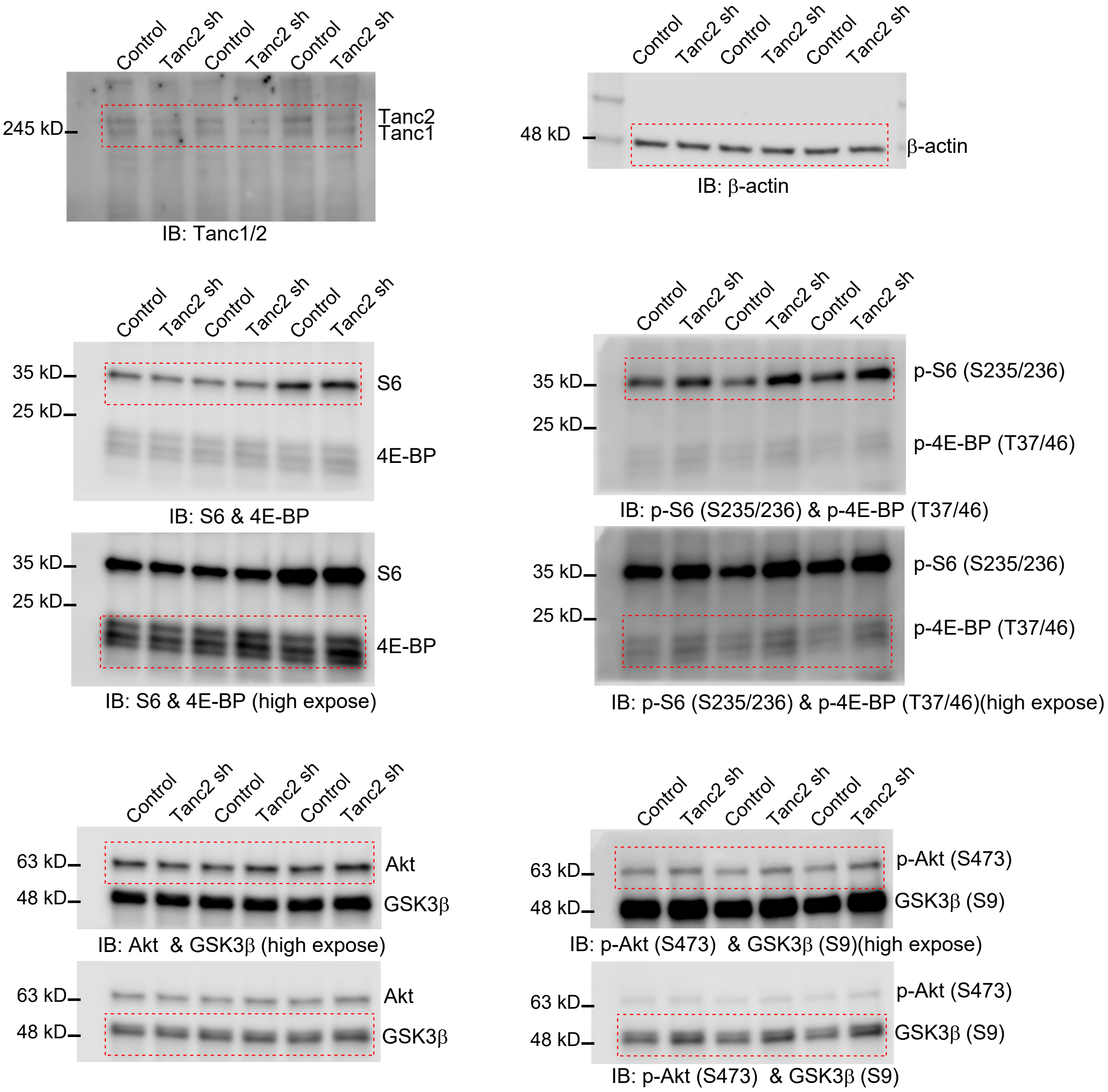

Fig. S8 b uncropped images

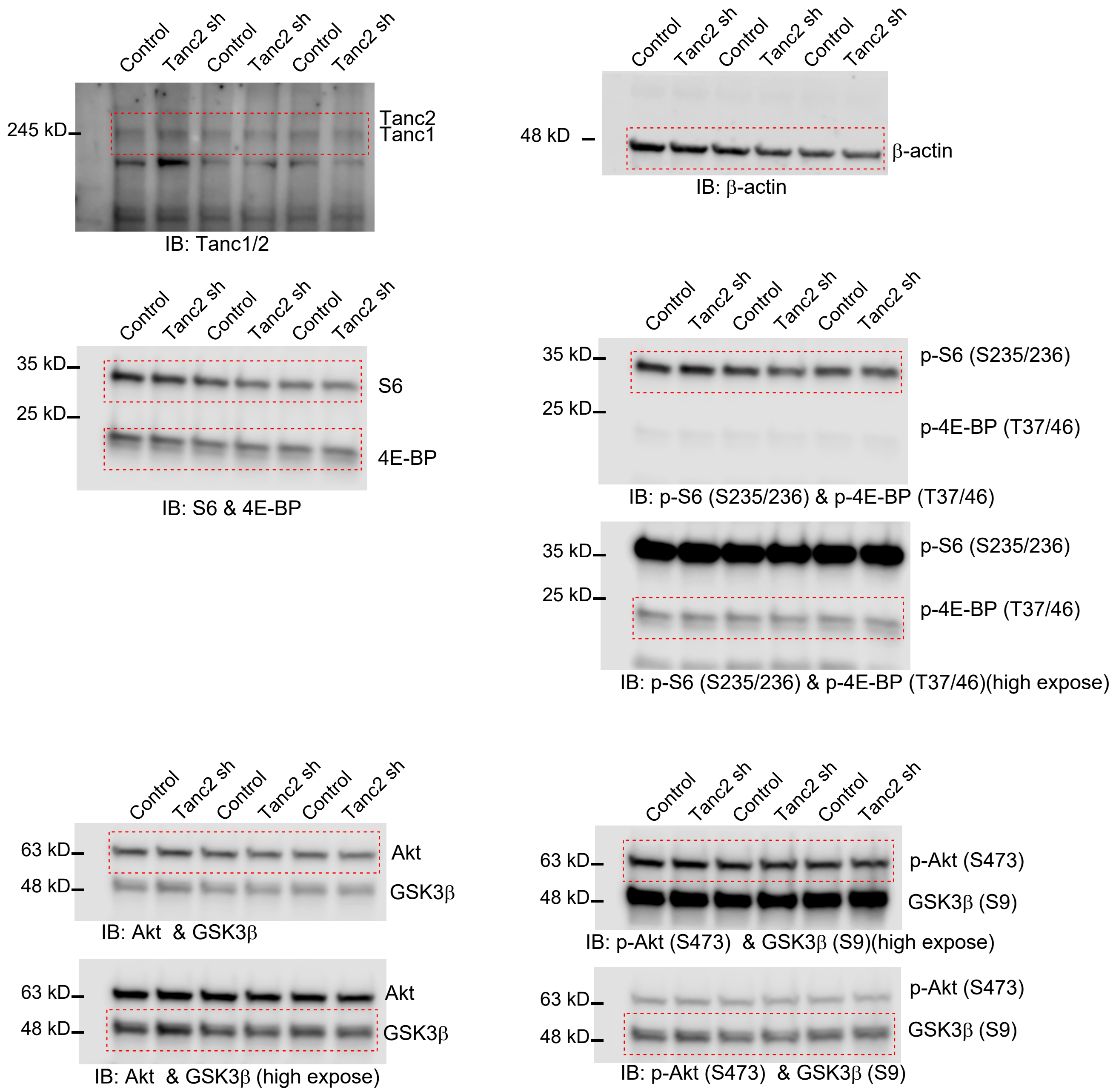

Supplement: Supplementary file 4 — Source Data [file 41467_2021_22908_MOESM4_ESM.zip › Source data/Source data 2.pdf]
